# Fast index based algorithms and software for matching position specific scoring matrices

**DOI:** 10.1186/1471-2105-7-389

**Published:** 2006-08-24

**Authors:** Michael Beckstette, Robert Homann, Robert Giegerich, Stefan Kurtz

**Affiliations:** 1 International NRW Graduate School in Bioinformatics and Genome Research, Center for Biotechnology (CeBITec), Bielefeld University, D-33594 Bielefeld, Germany; 2 Technische Fakultät, Universität Bielefeld, Postfach 100 131, D-33501 Bielefeld, Germany; 3 Zentrum für Bioinformatik, Universität Hamburg, 20146 Hamburg, Germany

## Abstract

**Background:**

In biological sequence analysis, position specific scoring matrices (PSSMs) are widely used to represent sequence motifs in nucleotide as well as amino acid sequences. Searching with PSSMs in complete genomes or large sequence databases is a common, but computationally expensive task.

**Results:**

We present a new non-heuristic algorithm, called *ESAsearch*, to efficiently find matches of PSSMs in large databases. Our approach preprocesses the search space, e.g., a complete genome or a set of protein sequences, and builds an enhanced suffix array that is stored on file. This allows the searching of a database with a PSSM in sublinear expected time. Since *ESAsearch *benefits from small alphabets, we present a variant operating on sequences recoded according to a reduced alphabet. We also address the problem of non-comparable PSSM-scores by developing a method which allows the efficient computation of a matrix similarity threshold for a PSSM, given an E-value or a p-value. Our method is based on dynamic programming and, in contrast to other methods, it employs lazy evaluation of the dynamic programming matrix. We evaluated algorithm *ESAsearch *with nucleotide PSSMs and with amino acid PSSMs. Compared to the best previous methods, *ESAsearch *shows speedups of a factor between 17 and 275 for nucleotide PSSMs, and speedups up to factor 1.8 for amino acid PSSMs. Comparisons with the most widely used programs even show speedups by a factor of at least 3.8. Alphabet reduction yields an additional speedup factor of 2 on amino acid sequences compared to results achieved with the 20 symbol standard alphabet. The lazy evaluation method is also much faster than previous methods, with speedups of a factor between 3 and 330.

**Conclusion:**

Our analysis of *ESAsearch *reveals sublinear runtime in the expected case, and linear runtime in the worst case for sequences not shorter than |A
 MathType@MTEF@5@5@+=feaafiart1ev1aaatCvAUfKttLearuWrP9MDH5MBPbIqV92AaeXatLxBI9gBamrtHrhAL1wy0L2yHvtyaeHbnfgDOvwBHrxAJfwnaebbnrfifHhDYfgasaacH8akY=wiFfYdH8Gipec8Eeeu0xXdbba9frFj0=OqFfea0dXdd9vqai=hGuQ8kuc9pgc9s8qqaq=dirpe0xb9q8qiLsFr0=vr0=vr0dc8meaabaqaciaacaGaaeqabaWaaeGaeaaakeaaimaacqWFaeFqaaa@3821@|^*m *^+ *m *- 1, where *m *is the length of the PSSM and A
 MathType@MTEF@5@5@+=feaafiart1ev1aaatCvAUfKttLearuWrP9MDH5MBPbIqV92AaeXatLxBI9gBamrtHrhAL1wy0L2yHvtyaeHbnfgDOvwBHrxAJfwnaebbnrfifHhDYfgasaacH8akY=wiFfYdH8Gipec8Eeeu0xXdbba9frFj0=OqFfea0dXdd9vqai=hGuQ8kuc9pgc9s8qqaq=dirpe0xb9q8qiLsFr0=vr0=vr0dc8meaabaqaciaacaGaaeqabaWaaeGaeaaakeaaimaacqWFaeFqaaa@3821@ a finite alphabet. In practice, *ESAsearch *shows superior performance over the most widely used programs, especially for DNA sequences. The new algorithm for accurate on-the-fly calculations of thresholds has the potential to replace formerly used approximation approaches. Beyond the algorithmic contributions, we provide a robust, well documented, and easy to use software package, implementing the ideas and algorithms presented in this manuscript.

## Background

Position specific scoring matrices (PSSMs) have a long history in sequence analysis (see [1]). A high PSSM-score in some region of a sequence often indicates a possible biological relationship of this sequence to the family or motif characterized by the PSSM. There are several databases utilizing PSSMs for function assignment and annotation, e.g., PROSITE [2], PRINTS [3], BLOCKS [4], EMATRIX [5], JASPAR [6], or TRANSFAC [7]. While these databases are constantly improved, there are only few improvements in the programs searching with PSSMs. E.g., the programs *FingerPrintScan *[8], *BLIMPS *[4], and *MatInspector*[9] still use a straightforward O
 MathType@MTEF@5@5@+=feaafiart1ev1aaatCvAUfKttLearuWrP9MDH5MBPbIqV92AaeXatLxBI9gBamrtHrhAL1wy0L2yHvtyaeHbnfgDOvwBHrxAJfwnaebbnrfifHhDYfgasaacH8akY=wiFfYdH8Gipec8Eeeu0xXdbba9frFj0=OqFfea0dXdd9vqai=hGuQ8kuc9pgc9s8qqaq=dirpe0xb9q8qiLsFr0=vr0=vr0dc8meaabaqaciaacaGaaeqabaWaaeGaeaaakeaaimaacqWFoe=taaa@383D@(*mn*)-time algorithm to search a PSSM of length *m *in a sequence of length *n*. In [10] the authors presented a method based on Fourier transformation. A different method introduced in [11] employs data compression. To the best of our knowledge there is no software available implementing these two methods. The most advanced program in the field of searching with PSSMs is *EMATRIX *[12], which incorporates a technique called lookahead scoring. The lookahead scoring technique is also employed in the suffix tree based method of [13]. This method performs a limited depth first traversal of the suffix tree of the set of target sequences. This search updates PSSM-scores along the edges of the suffix tree. Lookahead scoring allows to skip subtrees of the suffix tree that do not contain any matches to the PSSM. Unfortunately, the method of [13] has not found its way into a widely available and robust software system. In [14], the development of new, more efficient algorithms for searching with PSSMs is considered an important problem, which still needs better solutions.

In this paper, we present a new, non-heuristic algorithm for searching with PSSMs. With any non-heuristic PSSM searching algorithm, the performance in terms of sensitivity and specificity solely depends on the used PSSM and threshold, i.e. given a PSSM and threshold, all these algorithms give exactly the same results. For the generation of PSSMs from aligned sequences, numerous different methods, were described in literature over the last decades [1,5,15-17]. Rather than improving PSSMs, we focus on improvements in terms of time and space efficiency when searching with PSSMs, independently of their underlying generation method. The overall structure of our proposed search algorithm is similar to the method of [13]. However, instead of suffix trees we use enhanced suffix arrays, a data structure which is as powerful as suffix trees (cf. [18]). Enhanced suffix arrays provide several advantages over suffix trees, which make them more suitable for searching with PSSMs:

• While suffix trees require about 12*n *bytes in the best available implementation (cf. [19]), the enhanced suffix array used for searching with PSSMs only needs 9*n *bytes of space.

• While the suffix tree is usually only computed in main memory, the enhanced suffix array is computed once and stored on file. Whenever a PSSM is to be searched, the enhanced suffix array is mapped into main memory which requires no extra time.

• While the depth first traversal of the suffix tree suffers from the poor locality behavior of the data structure (cf. [20]), the enhanced suffix array provides optimal locality, because when searching with PSSMs it is sequentially scanned from left to right.

One of the algorithmic contributions of this paper is a new technique that allows to skip parts of the enhanced suffix array containing no matches to the PSSM. Due to the skipping, our algorithm achieves an expected running time that is sublinear in the size of the search space (i.e., the size of the nucleotide or protein database). As a consequence, our algorithm scales very well for large data sizes.

Since the running time of our algorithm increases with the size of the underlying alphabet, we developed a filtering technique, utilizing alphabet reduction, that achieves better performance especially on sequences/PSSMs over the amino acid alphabet.

When searching with a PSSM, it is important to determine a suitable threshold for a PSSM-match. Usually, the user prefers to specify a significance threshold (i.e., an E-value or a p-value) which has to be transformed into an absolute score threshold for the PSSM under consideration. This can be done by computing the score distribution of the PSSM, using well-known dynamic programming (DP, for short) methods, e.g., [12,21-23]. Unfortunately, these methods are not fast enough for large PSSMs. For this reason, we have developed a new, lazy evaluation algorithm that only computes a small fraction of the complete score distribution. Our algorithm speeds up the computation of the threshold by factor of at least 3, compared to standard DP methods. This makes our algorithm applicable for on-the-fly computations of the score thresholds.

The new algorithms described in this paper are implemented as part of the *PoSSuM *software distribution. This is available free of charge for non-commercial research institutions. For details, see [24]. Parts of this contribution appeared as [25] in proceedings of GCB2004.

## Results

### PSSMs and lookahead scoring: LAsearch

**Figure 1:**
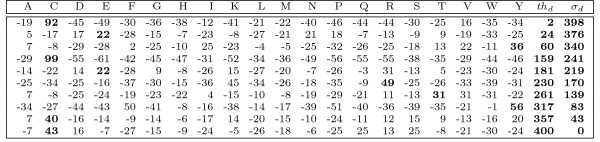
**Amino acid PSSM**. Amino acid PSSM of length *m *= 10 of a zinc-finger motif. If the score threshold is *th *= 400, then only substrings beginning with *C *or *V *can match the PSSM, because all other amino acids score below the intermediate threshold *th*_0 _= *th *- *σ*_0 _= 400 - 398 = 2. That is, lookahead scoring will skip over all substrings which begin with amino acids different from *C *and *V*. Here *σ*_*d*_, *d *∈ [0, *m *- 1] denotes the maximal score that can be achieved in the last *m *- *d *- 1 positions of the PSSM as defined in the text.

A PSSM is an abstraction of a multiple alignment of related sequences. We define it as a function *M *: [0, *m *- 1] × A
 MathType@MTEF@5@5@+=feaafiart1ev1aaatCvAUfKttLearuWrP9MDH5MBPbIqV92AaeXatLxBI9gBamrtHrhAL1wy0L2yHvtyaeHbnfgDOvwBHrxAJfwnaebbnrfifHhDYfgasaacH8akY=wiFfYdH8Gipec8Eeeu0xXdbba9frFj0=OqFfea0dXdd9vqai=hGuQ8kuc9pgc9s8qqaq=dirpe0xb9q8qiLsFr0=vr0=vr0dc8meaabaqaciaacaGaaeqabaWaaeGaeaaakeaaimaacqWFaeFqaaa@3821@ → ℝ, where *m *is the length of *M *and A
 MathType@MTEF@5@5@+=feaafiart1ev1aaatCvAUfKttLearuWrP9MDH5MBPbIqV92AaeXatLxBI9gBamrtHrhAL1wy0L2yHvtyaeHbnfgDOvwBHrxAJfwnaebbnrfifHhDYfgasaacH8akY=wiFfYdH8Gipec8Eeeu0xXdbba9frFj0=OqFfea0dXdd9vqai=hGuQ8kuc9pgc9s8qqaq=dirpe0xb9q8qiLsFr0=vr0=vr0dc8meaabaqaciaacaGaaeqabaWaaeGaeaaakeaaimaacqWFaeFqaaa@3821@ is a finite alphabet. Usually *M *is represented by an *m *× |A
 MathType@MTEF@5@5@+=feaafiart1ev1aaatCvAUfKttLearuWrP9MDH5MBPbIqV92AaeXatLxBI9gBamrtHrhAL1wy0L2yHvtyaeHbnfgDOvwBHrxAJfwnaebbnrfifHhDYfgasaacH8akY=wiFfYdH8Gipec8Eeeu0xXdbba9frFj0=OqFfea0dXdd9vqai=hGuQ8kuc9pgc9s8qqaq=dirpe0xb9q8qiLsFr0=vr0=vr0dc8meaabaqaciaacaGaaeqabaWaaeGaeaaakeaaimaacqWFaeFqaaa@3821@| matrix, see Figure 1 for an example. Each row of the matrix reflects the frequency of occurrence of each amino acid or nucleotide at the corresponding position of the alignment. From now on, let M be a PSSM of length *m *and let *w*[*i*] denote the character of *w *at position *i *for 0 ≤ *i < m*. Further on, *w*[*i*..*j*] denotes the string starting at position *i *and ending at position *j*. We define sc(w,M):=∑i=0m−1M(i,w[i])
 MathType@MTEF@5@5@+=feaafiart1ev1aaatCvAUfKttLearuWrP9MDH5MBPbIqV92AaeXatLxBI9gBaebbnrfifHhDYfgasaacH8akY=wiFfYdH8Gipec8Eeeu0xXdbba9frFj0=OqFfea0dXdd9vqai=hGuQ8kuc9pgc9s8qqaq=dirpe0xb9q8qiLsFr0=vr0=vr0dc8meaabaqaciaacaGaaeqabaqabeGadaaakeaacqWGZbWCcqWGJbWydaqadiqaaiabdEha3jabcYcaSiabd2eanbGaayjkaiaawMcaaiabcQda6iabg2da9maaqadabaGaemyta00aaeWaceaacqWGPbqAcqGGSaalcqWG3bWDcqGGBbWwcqWGPbqAcqGGDbqxaiaawIcacaGLPaaaaSqaaiabdMgaPjabg2da9iabicdaWaqaaiabd2gaTjabgkHiTiabigdaXaqdcqGHris5aaaa@493D@ for a sequence *w *∈ A
 MathType@MTEF@5@5@+=feaafiart1ev1aaatCvAUfKttLearuWrP9MDH5MBPbIqV92AaeXatLxBI9gBamrtHrhAL1wy0L2yHvtyaeHbnfgDOvwBHrxAJfwnaebbnrfifHhDYfgasaacH8akY=wiFfYdH8Gipec8Eeeu0xXdbba9frFj0=OqFfea0dXdd9vqai=hGuQ8kuc9pgc9s8qqaq=dirpe0xb9q8qiLsFr0=vr0=vr0dc8meaabaqaciaacaGaaeqabaWaaeGaeaaakeaaimaacqWFaeFqaaa@3821@^*m *^of length *m*. *sc *(*w*, *M*) is the *match score of w *w.r.t. *M*. The *score range *of a PSSM is the interval [*sc*_min_(*M*), *sc*_max_(*M*)] with scmin⁡(M):=∑i=0m−1min⁡{M(i,a)|a∈A}
 MathType@MTEF@5@5@+=feaafiart1ev1aaatCvAUfKttLearuWrP9MDH5MBPbIqV92AaeXatLxBI9gBamrtHrhAL1wy0L2yHvtyaeHbnfgDOvwBHrxAJfwnaebbnrfifHhDYfgasaacH8akY=wiFfYdH8Gipec8Eeeu0xXdbba9frFj0=OqFfea0dXdd9vqai=hGuQ8kuc9pgc9s8qqaq=dirpe0xb9q8qiLsFr0=vr0=vr0dc8meaabaqaciaacaGaaeqabaWaaeGaeaaakeaacqWGZbWCcqWGJbWydaWgaaWcbaGagiyBa0MaeiyAaKMaeiOBa4gabeaakiabcIcaOiabd2eanjabcMcaPiabcQda6iabg2da9maaqadabaGagiyBa0MaeiyAaKMaeiOBa4galeaacqWGPbqAcqGH9aqpcqaIWaamaeaacqWGTbqBcqGHsislcqaIXaqma0GaeyyeIuoakiabcUha7jabd2eanjabcIcaOiabdMgaPjabcYcaSiabdggaHjabcMcaPiabcYha8jabdggaHjabgIGioJWaaiab=bq8bjabc2ha9baa@5E75@ and scmax⁡(M):=∑i=0m−1max⁡{M(i,a)|a∈A}
 MathType@MTEF@5@5@+=feaafiart1ev1aaatCvAUfKttLearuWrP9MDH5MBPbIqV92AaeXatLxBI9gBamrtHrhAL1wy0L2yHvtyaeHbnfgDOvwBHrxAJfwnaebbnrfifHhDYfgasaacH8akY=wiFfYdH8Gipec8Eeeu0xXdbba9frFj0=OqFfea0dXdd9vqai=hGuQ8kuc9pgc9s8qqaq=dirpe0xb9q8qiLsFr0=vr0=vr0dc8meaabaqaciaacaGaaeqabaWaaeGaeaaakeaacqWGZbWCcqWGJbWydaWgaaWcbaGagiyBa0MaeiyyaeMaeiiEaGhabeaakiabcIcaOiabd2eanjabcMcaPiabcQda6iabg2da9maaqadabaGagiyBa0MaeiyyaeMaeiiEaGhaleaacqWGPbqAcqGH9aqpcqaIWaamaeaacqWGTbqBcqGHsislcqaIXaqma0GaeyyeIuoakiabcUha7jabd2eanjabcIcaOiabdMgaPjabcYcaSiabdggaHjabcMcaPiabcYha8jabdggaHjabgIGioJWaaiab=bq8bjabc2ha9baa@5E7D@. Given a sequence *S *of length *n *over alphabet A
 MathType@MTEF@5@5@+=feaafiart1ev1aaatCvAUfKttLearuWrP9MDH5MBPbIqV92AaeXatLxBI9gBamrtHrhAL1wy0L2yHvtyaeHbnfgDOvwBHrxAJfwnaebbnrfifHhDYfgasaacH8akY=wiFfYdH8Gipec8Eeeu0xXdbba9frFj0=OqFfea0dXdd9vqai=hGuQ8kuc9pgc9s8qqaq=dirpe0xb9q8qiLsFr0=vr0=vr0dc8meaabaqaciaacaGaaeqabaWaaeGaeaaakeaaimaacqWFaeFqaaa@3821@ and a score threshold *th*, the *PSSM matching problem *is to find all positions *j *∈ [0, *n *- *m*] in *S *and their assigned match scores, such that *sc *(*S*[*j..j *+ *m *- 1], *M*) ≥ *th*.

A simple algorithm for the PSSM matching problem slides along the sequence and computes *sc *(*w*, *M*) for each *w *= *S *[*j..j *+ *m *- 1], *j *∈ [0, *n *- *m*]. The running time of this algorithm is O
 MathType@MTEF@5@5@+=feaafiart1ev1aaatCvAUfKttLearuWrP9MDH5MBPbIqV92AaeXatLxBI9gBamrtHrhAL1wy0L2yHvtyaeHbnfgDOvwBHrxAJfwnaebbnrfifHhDYfgasaacH8akY=wiFfYdH8Gipec8Eeeu0xXdbba9frFj0=OqFfea0dXdd9vqai=hGuQ8kuc9pgc9s8qqaq=dirpe0xb9q8qiLsFr0=vr0=vr0dc8meaabaqaciaacaGaaeqabaWaaeGaeaaakeaaimaacqWFoe=taaa@383D@(*mn*). It is used e.g., in the programs *FingerPrintScan *[8], *BLIMPS *[4], *MatInspector*[9], and *MATCH *[17].

In [12], lookahead scoring is introduced to improve the simple algorithm. Lookahead scoring allows to stop the calculation of *sc *(*w*, *M*) when it is clear that the given overall score threshold *th *cannot be achieved. To be more precise, we define pfxscd(w,M):=∑h=0dM(h,w[h])
 MathType@MTEF@5@5@+=feaafiart1ev1aaatCvAUfKttLearuWrP9MDH5MBPbIqV92AaeXatLxBI9gBaebbnrfifHhDYfgasaacH8akY=wiFfYdH8Gipec8Eeeu0xXdbba9frFj0=OqFfea0dXdd9vqai=hGuQ8kuc9pgc9s8qqaq=dirpe0xb9q8qiLsFr0=vr0=vr0dc8meaabaqaciaacaGaaeqabaqabeGadaaakeaacqWGWbaCcqWGMbGzcqWG4baEcqWGZbWCcqWGJbWydaWgaaWcbaGaemizaqgabeaakmaabmGabaGaem4DaCNaeiilaWIaemyta0eacaGLOaGaayzkaaGaeiOoaOJaeyypa0ZaaabmaeaacqWGnbqtdaqadiqaaiabdIgaOjabcYcaSiabdEha3jabcUfaBjabdIgaOjabc2faDbGaayjkaiaawMcaaaWcbaGaemiAaGMaeyypa0JaeGimaadabaGaemizaqganiabggHiLdaaaa@4D06@, *max*_*d *_:= max{*M*(*d*, *a*) | *a *∈ A
 MathType@MTEF@5@5@+=feaafiart1ev1aaatCvAUfKttLearuWrP9MDH5MBPbIqV92AaeXatLxBI9gBamrtHrhAL1wy0L2yHvtyaeHbnfgDOvwBHrxAJfwnaebbnrfifHhDYfgasaacH8akY=wiFfYdH8Gipec8Eeeu0xXdbba9frFj0=OqFfea0dXdd9vqai=hGuQ8kuc9pgc9s8qqaq=dirpe0xb9q8qiLsFr0=vr0=vr0dc8meaabaqaciaacaGaaeqabaWaaeGaeaaakeaaimaacqWFaeFqaaa@3821@}, and σd:=∑h=d+1m−1maxh
 MathType@MTEF@5@5@+=feaafiart1ev1aaatCvAUfKttLearuWrP9MDH5MBPbIqV92AaeXatLxBI9gBaebbnrfifHhDYfgasaacH8akY=wiFfYdH8Gipec8Eeeu0xXdbba9frFj0=OqFfea0dXdd9vqai=hGuQ8kuc9pgc9s8qqaq=dirpe0xb9q8qiLsFr0=vr0=vr0dc8meaabaqaciaacaGaaeqabaqabeGadaaakeaaiiGacqWFdpWCdaWgaaWcbaGaemizaqgabeaakiabcQda6iabg2da9maaqadabaacbiGae4xBa0Mae4xyaeMae4hEaG3aaSbaaSqaaiabdIgaObqabaaabaGaemiAaGMaeyypa0JaemizaqMaey4kaSIaeGymaedabaGaemyBa0MaeyOeI0IaeGymaedaniabggHiLdaaaa@4260@ for any *d *∈ [0, *m *- 1]. *pfxsc*_*d*_(*w, M*) is the *prefix score of depth d*. *σ*_*d *_is the maximal score that can be achieved in the last *m *- *d *- 1 positions of the PSSM. Let *th*_*d *_:= *th *- *σ*_*d *_be the *intermediate threshold *at position *d*. The correctness of lookahead scoring, not shown in [12], is implied by the following Lemma:

**Lemma 1 **The following statements are equivalent:

(1) *pfxsc*_*d *_(*w*, *M*) ≥ *th*_*d *_for all *d *∈ [0, *m *- 1],

(2) *sc*(*w*, *M*) ≥ *th*.

**Proof**: (1)⇒(2): Suppose that (1) holds. Then σm−1=∑h=mm−1maxh=0
 MathType@MTEF@5@5@+=feaafiart1ev1aaatCvAUfKttLearuWrP9MDH5MBPbIqV92AaeXatLxBI9gBaebbnrfifHhDYfgasaacH8akY=wiFfYdH8Gipec8Eeeu0xXdbba9frFj0=OqFfea0dXdd9vqai=hGuQ8kuc9pgc9s8qqaq=dirpe0xb9q8qiLsFr0=vr0=vr0dc8meaabaqaciaacaGaaeqabaqabeGadaaakeaaiiGacqWFdpWCdaWgaaWcbaGaemyBa0MaeyOeI0IaeGymaedabeaakiabg2da9maaqadabaacbiGae4xBa0Mae4xyaeMae4hEaG3aaSbaaSqaaiabdIgaObqabaGccqGH9aqpcqaIWaamaSqaaiabdIgaOjabg2da9iabd2gaTbqaaiabd2gaTjabgkHiTiabigdaXaqdcqGHris5aaaa@439C@ and

sc(w,M)=∑h=0m−1M(h,w[h])=  pfxscm−1(w,M)≥thm−1=th−σm−1=th.
 MathType@MTEF@5@5@+=feaafiart1ev1aaatCvAUfKttLearuWrP9MDH5MBPbIqV92AaeXatLxBI9gBaebbnrfifHhDYfgasaacH8akY=wiFfYdH8Gipec8Eeeu0xXdbba9frFj0=OqFfea0dXdd9vqai=hGuQ8kuc9pgc9s8qqaq=dirpe0xb9q8qiLsFr0=vr0=vr0dc8meaabaqaciaacaGaaeqabaqabeGadaaakeaacqWGZbWCcqWGJbWydaqadiqaaiabdEha3jabcYcaSiabd2eanbGaayjkaiaawMcaaiabg2da9maaqahabaGaemyta00aaeWaceaacqWGObaAcqGGSaalcqWG3bWDcqGGBbWwcqWGObaAcqGGDbqxaiaawIcacaGLPaaacqGH9aqpaSqaaiabdIgaOjabg2da9iabicdaWaqaaiabd2gaTjabgkHiTiabigdaXaqdcqGHris5aOGaaGPaVlaaykW7cqWGWbaCcqWGMbGzcqWG4baEcqWGZbWCcqWGJbWydaWgaaWcbaGaemyBa0MaeyOeI0IaeGymaedabeaakmaabmGabaGaem4DaCNaeiilaWIaemyta0eacaGLOaGaayzkaaGaeyyzImRaemiDaqNaemiAaG2aaSbaaSqaaiabd2gaTjabgkHiTiabigdaXaqabaGccqGH9aqpcqWG0baDcqWGObaAcqGHsisliiGacqWFdpWCdaWgaaWcbaGaemyBa0MaeyOeI0IaeGymaedabeaakiabg2da9iabdsha0jabdIgaOjabc6caUaaa@72C8@

(2)⇒(1): Suppose that (2) holds. Let *d *∈ [0, *m *- 1]. Then

sc(w,M)=∑h=0m−1M(h,w[h])=∑h=0dM(h,w[h])+∑h=d+1m−1M(h,w[h])  =pfxscd(w,M)+∑h=d+1m−1M(h,w[h])
 MathType@MTEF@5@5@+=feaafiart1ev1aaatCvAUfKttLearuWrP9MDH5MBPbIqV92AaeXatLxBI9gBaebbnrfifHhDYfgasaacH8akY=wiFfYdH8Gipec8Eeeu0xXdbba9frFj0=OqFfea0dXdd9vqai=hGuQ8kuc9pgc9s8qqaq=dirpe0xb9q8qiLsFr0=vr0=vr0dc8meaabaqaciaacaGaaeqabaqabeGadaaakqaabeqaaiabdohaZjabdogaJnaabmGabaGaem4DaCNaeiilaWIaemyta0eacaGLOaGaayzkaaGaeyypa0ZaaabCaeaacqWGnbqtdaqadiqaaiabdIgaOjabcYcaSiabdEha3jabcUfaBjabdIgaOjabc2faDbGaayjkaiaawMcaaaWcbaGaemiAaGMaeyypa0JaeGimaadabaGaemyBa0MaeyOeI0IaeGymaedaniabggHiLdGccqGH9aqpdaaeWbqaaiabd2eannaabmGabaGaemiAaGMaeiilaWIaem4DaCNaei4waSLaemiAaGMaeiyxa0facaGLOaGaayzkaaaaleaacqWGObaAcqGH9aqpcqaIWaamaeaacqWGKbaza0GaeyyeIuoakiabgUcaRmaaqahabaGaemyta00aaeWaceaacqWGObaAcqGGSaalcqWG3bWDcqGGBbWwcqWGObaAcqGGDbqxaiaawIcacaGLPaaaaSqaaiabdIgaOjabg2da9iabdsgaKjabgUcaRiabigdaXaqaaiabd2gaTjabgkHiTiabigdaXaqdcqGHris5aaGcbaGaaCzcaiaaykW7caaMc8Uaeyypa0JaemiCaaNaemOzayMaemiEaGNaem4CamNaem4yam2aaSbaaSqaaiabdsgaKbqabaGcdaqadiqaaiabdEha3jabcYcaSiabd2eanbGaayjkaiaawMcaaiabgUcaRmaaqahabaGaemyta00aaeWaceaacqWGObaAcqGGSaalcqWG3bWDcqGGBbWwcqWGObaAcqGGDbqxaiaawIcacaGLPaaaaSqaaiabdIgaOjabg2da9iabdsgaKjabgUcaRiabigdaXaqaaiabd2gaTjabgkHiTiabigdaXaqdcqGHris5aaaaaa@9936@

Hence *sc*(*w*, *M*) ≥ *th *implies pfxscd(w,M)+∑h=d+1m−1M(h,w[h])≥th
 MathType@MTEF@5@5@+=feaafiart1ev1aaatCvAUfKttLearuWrP9MDH5MBPbIqV92AaeXatLxBI9gBaebbnrfifHhDYfgasaacH8akY=wiFfYdH8Gipec8Eeeu0xXdbba9frFj0=OqFfea0dXdd9vqai=hGuQ8kuc9pgc9s8qqaq=dirpe0xb9q8qiLsFr0=vr0=vr0dc8meaabaqaciaacaGaaeqabaqabeGadaaakeaacqWGWbaCcqWGMbGzcqWG4baEcqWGZbWCcqWGJbWydaWgaaWcbaGaemizaqgabeaakmaabmGabaGaem4DaCNaeiilaWIaemyta0eacaGLOaGaayzkaaGaey4kaSYaaabmaeaacqWGnbqtdaqadiqaaiabdIgaOjabcYcaSiabdEha3jabcUfaBjabdIgaOjabc2faDbGaayjkaiaawMcaaaWcbaGaemiAaGMaeyypa0JaemizaqMaey4kaSIaeGymaedabaGaemyBa0MaeyOeI0IaeGymaedaniabggHiLdGccqGHLjYScqWG0baDcqWGObaAaaa@54A4@. Since *M*(*h*, *w*[*h*]) ≤ *max*_*h *_for *h *∈ [0, *m *- 1], we conclude

∑h=d+1m−1M(h,w[h])  ≤  ∑h=d+1m−1maxh=σd
 MathType@MTEF@5@5@+=feaafiart1ev1aaatCvAUfKttLearuWrP9MDH5MBPbIqV92AaeXatLxBI9gBaebbnrfifHhDYfgasaacH8akY=wiFfYdH8Gipec8Eeeu0xXdbba9frFj0=OqFfea0dXdd9vqai=hGuQ8kuc9pgc9s8qqaq=dirpe0xb9q8qiLsFr0=vr0=vr0dc8meaabaqaciaacaGaaeqabaqabeGadaaakeaadaaeWbqaaiabd2eannaabmGabaGaemiAaGMaeiilaWIaem4DaCNaei4waSLaemiAaGMaeiyxa0facaGLOaGaayzkaaaaleaacqWGObaAcqGH9aqpcqWGKbazcqGHRaWkcqaIXaqmaeaacqWGTbqBcqGHsislcqaIXaqma0GaeyyeIuoakiaaykW7caaMc8UaeyizImQaaGPaVlaaykW7daaeWbqaaGqaciab=1gaTjab=fgaHjab=Hha4naaBaaaleaacqWGObaAaeqaaaqaaiabdIgaOjabg2da9iabdsgaKjabgUcaRiabigdaXaqaaiabd2gaTjabgkHiTiabigdaXaqdcqGHris5aOGaeyypa0dcciGae43Wdm3aaSbaaSqaaiabdsgaKbqabaaaaa@5ECC@

and hence

pfxscd(w,M)≥th−∑h=d+1m−1M(h,w[h])≥th−σd=thd.
 MathType@MTEF@5@5@+=feaafiart1ev1aaatCvAUfKttLearuWrP9MDH5MBPbIqV92AaeXatLxBI9gBaebbnrfifHhDYfgasaacH8akY=wiFfYdH8Gipec8Eeeu0xXdbba9frFj0=OqFfea0dXdd9vqai=hGuQ8kuc9pgc9s8qqaq=dirpe0xb9q8qiLsFr0=vr0=vr0dc8meaabaqaciaacaGaaeqabaqabeGadaaakeaacqWGWbaCcqWGMbGzcqWG4baEcqWGZbWCcqWGJbWydaWgaaWcbaGaemizaqgabeaakmaabmGabaGaem4DaCNaeiilaWIaemyta0eacaGLOaGaayzkaaGaeyyzImRaemiDaqNaemiAaGMaeyOeI0YaaabCaeaacqWGnbqtdaqadiqaaiabdIgaOjabcYcaSiabdEha3jabcUfaBjabdIgaOjabc2faDbGaayjkaiaawMcaaaWcbaGaemiAaGMaeyypa0JaemizaqMaey4kaSIaeGymaedabaGaemyBa0MaeyOeI0IaeGymaedaniabggHiLdGccqGHLjYScqWG0baDcqWGObaAcqGHsisliiGacqWFdpWCdaWgaaWcbaGaemizaqgabeaakiabg2da9iabdsha0jabdIgaOnaaBaaaleaacqWGKbazaeqaaOGaeiOla4caaa@63F8@

The Lemma suggests a necessary condition for a PSSM-match which can easily be exploited: When computing *sc*(*w*, *M*) by scanning *w *from left to right, one checks for *d *= 0,1,..., *m *- 1, if the intermediate threshold *th*_*d *_is achieved. If not, the computation can be stopped. See Figure 1 for an example of intermediate thresholds and their implications.

The lookahead scoring algorithm (herein after called *LAsearch*) runs in O
 MathType@MTEF@5@5@+=feaafiart1ev1aaatCvAUfKttLearuWrP9MDH5MBPbIqV92AaeXatLxBI9gBamrtHrhAL1wy0L2yHvtyaeHbnfgDOvwBHrxAJfwnaebbnrfifHhDYfgasaacH8akY=wiFfYdH8Gipec8Eeeu0xXdbba9frFj0=OqFfea0dXdd9vqai=hGuQ8kuc9pgc9s8qqaq=dirpe0xb9q8qiLsFr0=vr0=vr0dc8meaabaqaciaacaGaaeqabaWaaeGaeaaakeaaimaacqWFoe=taaa@383D@(*kn*) time, where *k *is the average number of PSSM-positions per sequence position actually evaluated. In the worst case, *k *∈ O
 MathType@MTEF@5@5@+=feaafiart1ev1aaatCvAUfKttLearuWrP9MDH5MBPbIqV92AaeXatLxBI9gBamrtHrhAL1wy0L2yHvtyaeHbnfgDOvwBHrxAJfwnaebbnrfifHhDYfgasaacH8akY=wiFfYdH8Gipec8Eeeu0xXdbba9frFj0=OqFfea0dXdd9vqai=hGuQ8kuc9pgc9s8qqaq=dirpe0xb9q8qiLsFr0=vr0=vr0dc8meaabaqaciaacaGaaeqabaWaaeGaeaaakeaaimaacqWFoe=taaa@383D@(*m*), which leads to the worst case running time of O
 MathType@MTEF@5@5@+=feaafiart1ev1aaatCvAUfKttLearuWrP9MDH5MBPbIqV92AaeXatLxBI9gBamrtHrhAL1wy0L2yHvtyaeHbnfgDOvwBHrxAJfwnaebbnrfifHhDYfgasaacH8akY=wiFfYdH8Gipec8Eeeu0xXdbba9frFj0=OqFfea0dXdd9vqai=hGuQ8kuc9pgc9s8qqaq=dirpe0xb9q8qiLsFr0=vr0=vr0dc8meaabaqaciaacaGaaeqabaWaaeGaeaaakeaaimaacqWFoe=taaa@383D@(*mn*), not better than the simple algorithm. However, *k *is expected to be much smaller than *m*, leading to considerable speedups in practice.

Our reformulation of lookahead scoring and its implementation is the basis for improvements and evaluation in the subsequent sections.

### PSSM searching using enhanced suffix arrays: ESAsearch

**Figure 2:**
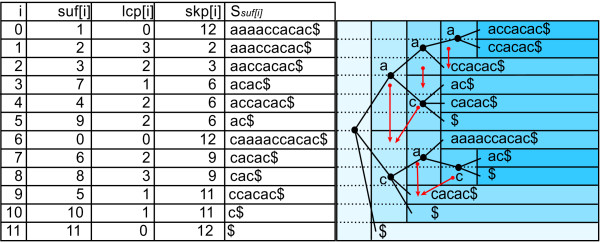
**Relationship between enhanced suffix array and suffix tree**. The enhanced suffix array consisting of tables suf, lcp, skp (left) and the suffix tree (right) for sequence *S *= caaaaccacac. Some skp entries are shown in the tree as red arrows: If skp[*i*] = *j*, then an arrow points from row *i *to row *j*. For clarity, suffixes corresponding to suf[*i*] are given in table *S*_suf[*i*]_.

The enhanced suffix array for a given sequence *S *of length *n *consists of three tables suf, lcp, and skp. Let $ be a symbol in A
 MathType@MTEF@5@5@+=feaafiart1ev1aaatCvAUfKttLearuWrP9MDH5MBPbIqV92AaeXatLxBI9gBamrtHrhAL1wy0L2yHvtyaeHbnfgDOvwBHrxAJfwnaebbnrfifHhDYfgasaacH8akY=wiFfYdH8Gipec8Eeeu0xXdbba9frFj0=OqFfea0dXdd9vqai=hGuQ8kuc9pgc9s8qqaq=dirpe0xb9q8qiLsFr0=vr0=vr0dc8meaabaqaciaacaGaaeqabaWaaeGaeaaakeaaimaacqWFaeFqaaa@3821@, larger than all other symbols, which does not occur in *S*. suf is a table of integers in the range 0 to *n*, specifying the lexicographic ordering of the *n *+ 1 suffixes of the string *S*$. That is, *S*_suf[0]_, *S*_suf[1]_, ... ,*S*_*suf*[*n*] _is the sequence of suffixes of *S*$ in ascending lexicographic order, where *S*_*i *_= *S*[*i..n *- 1]$ denotes the *i*-th nonempty suffix of the string *S*$, for *i *∈ [0, *n*]. See Figure 2 for an example. suf can be constructed in O
 MathType@MTEF@5@5@+=feaafiart1ev1aaatCvAUfKttLearuWrP9MDH5MBPbIqV92AaeXatLxBI9gBamrtHrhAL1wy0L2yHvtyaeHbnfgDOvwBHrxAJfwnaebbnrfifHhDYfgasaacH8akY=wiFfYdH8Gipec8Eeeu0xXdbba9frFj0=OqFfea0dXdd9vqai=hGuQ8kuc9pgc9s8qqaq=dirpe0xb9q8qiLsFr0=vr0=vr0dc8meaabaqaciaacaGaaeqabaWaaeGaeaaakeaaimaacqWFoe=taaa@383D@(*n*) time [26] and requires 4*n *bytes.

lcp is a table in the range 0 to *n *such that lcp[0] := 0 and lcp[*i*] is the length of the longest common prefix of *S*_suf[*i *- 1] _and *S*_suf[*i*]_, for *i *∈ [1, *n*]. See Figure 2 for an example. Table lcp can be computed in linear time given table suf [27]. In practice PSSMs are used to model relatively short, local motifs and hence do not exceed length 255. For searching with PSSMs we therefore do not access values in table lcp larger than 255, and hence we can store lcp in *n *bytes.

skp is a table in the range 0 to *n *such that skp[*i*] := min({*n *+ 1} ∪ {*j *∈ [*i *+ 1, *n*] | lcp[*i*] > lcp[*j*]}). In terms of suffix trees, skp[*i*] denotes the lexicographically next leaf that does not occur in the subtree below the branching node corresponding to the longest common prefix of *S*_suf[*i *- 1] _and *S*_suf[*i*]_. Figure 2 shows this relation. Table skp can be computed in O
 MathType@MTEF@5@5@+=feaafiart1ev1aaatCvAUfKttLearuWrP9MDH5MBPbIqV92AaeXatLxBI9gBamrtHrhAL1wy0L2yHvtyaeHbnfgDOvwBHrxAJfwnaebbnrfifHhDYfgasaacH8akY=wiFfYdH8Gipec8Eeeu0xXdbba9frFj0=OqFfea0dXdd9vqai=hGuQ8kuc9pgc9s8qqaq=dirpe0xb9q8qiLsFr0=vr0=vr0dc8meaabaqaciaacaGaaeqabaWaaeGaeaaakeaaimaacqWFoe=taaa@383D@(*n*) time given suf and lcp. For the algorithm to be described we assume that the enhanced suffix array for *S *has been precomputed.

In a suffix tree, all substrings of *S *of a fixed length *m *can be scored with a PSSM by a depth first traversal of the tree. Using lookahead scoring, one can skip certain subtrees that do not contain matches to the PSSM. Since suffix trees have several disadvantages (see the introduction), we use enhanced suffix arrays to search PSSMs. Like in other algorithms on enhanced suffix arrays (cf. [18]), one simulates a depth first traversal of the suffix tree by processing the arrays suf and lcp from left to right. To incorporate lookahead scoring while searching we must be able to skip certain ranges of suffixes in suf. To facilitate this, we use table skp. We will now make this more precise.

For *i *∈ [0, *n*], let *v*_*i *_= *S*_suf[*i*]_, *l*_*i *_= min{*m*, |*v*_*i*_|} - 1, and *d*_*i *_= max({-1} ∪ {*d *∈ [0, *l*_*i*_] |*pfxsc*_*d *_(*v*_*i*_, *M*) ≥ *th*_*d*_}). Now observe that *d*_*i *_= *m *- 1 ⇔ *pfxsc*_*m*-1 _(*v*_*i*_, *M*) ≥ *th*_*m*-1 _⇔ *sc *(*v*_*i*_, *M*) ≥ *th*. Hence, *M *matches at position *j *= suf[*i*] if and only if *d*_*i *_= *m *- 1. Thus, to solve the PSSM searching problem, it suffices to compute all *i *∈ [0, *n*] satisfying *d*_*i *_= *m *- 1. We compute *d*_*i *_along with *C*_*i*_[*d*] = *pfxsc*_*d *_(*v*_*i*_, *M*) for any *d *∈ [0, *d*_*i*_]. *d*_0 _and *C*_0 _are easily determined in O
 MathType@MTEF@5@5@+=feaafiart1ev1aaatCvAUfKttLearuWrP9MDH5MBPbIqV92AaeXatLxBI9gBamrtHrhAL1wy0L2yHvtyaeHbnfgDOvwBHrxAJfwnaebbnrfifHhDYfgasaacH8akY=wiFfYdH8Gipec8Eeeu0xXdbba9frFj0=OqFfea0dXdd9vqai=hGuQ8kuc9pgc9s8qqaq=dirpe0xb9q8qiLsFr0=vr0=vr0dc8meaabaqaciaacaGaaeqabaWaaeGaeaaakeaaimaacqWFoe=taaa@383D@(*m*) time. Now let *i *∈ [1, *n*] and suppose that *d*_*i*-1 _and *C*_*i*-1_[*d*] are determined for *d *∈ [0,*d*_*i*-1_]. Since *v*_*i*-1 _and *v*_*i *_have a common prefix of length lcp[*i*], we have *C*_*i*_[*d*] = *C*_*i*-1_[*d*] for all *d *∈ [0, lcp[*i*] - 1]. Consider the following cases:

• If *d*_*i*-1 _+ 1 ≥ lcp[*i*], then compute *C*_*i*_[*d*] for *d *≥ lcp[*i*] while *d *≤ *l*_*i *_and *C*_*i*_[*d*] ≥ *th*_*d*_. We obtain *d*_*i *_= *d*.

• If *d*_*i*-1 _+ 1 < lcp[*i*], then let *j *be the minimum value in the range [*i *+ 1, *n *+ 1] such that all suffixes *v*_*i*_, *v*_*i*+1_,...,*v*_*j*-1 _have a common prefix of length *d*_*i*-1_+ 1 with *v*_*i*-1_. Due to the common prefix we have *pfxsc*_*d*_(*v*_*i*-1_, *M*) = *pfxsc*_*d*_(*v*_*r*_, *M*) for all *d *∈ [0, *d*_*i*-1 _+ 1] and r ∈ [*i*, *j *- 1]. Hence *d*_*i*-1 _= *d*_*r *_for r ∈ [*i*, *j *- 1]. If *d*_*i*-1 _= *m *- 1, then there are PSSM matches at all positions suf[*r*] for *r *∈ [*i*, *j *- 1]. If *d*_*i*-1 _<*m *- 1, then there are no PSSM matches at any of these positions. That is, we can directly proceed with index *j*. We obtain *j *by following a chain of entries in table skp: compute a sequence of values *j*_0 _= *i*, *j*_1 _= skp[*j*_0_],...,*j*_*k *_= skp[*j*_*k*-1_] such that *d*_*i*-1 _+ 1 < lcp[*j*_1_],...,*d*_*i*-1 _+ 1 < lcp[*j*_*k*-1_], and *d*_*i*-1 _+ 1 ≥ lcp[*j*_*k*_]. Then *j *= *j*_*k*_.

**Figure 3:**
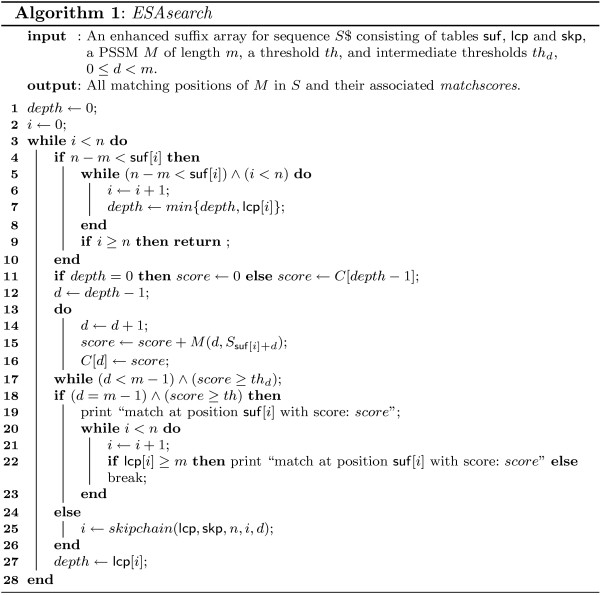
**Algorithm ESAsearch**. The algorithm *ESAsearch *formulated in pseudocode. See text for detailed explanations of the used notions.

**Figure 4:**
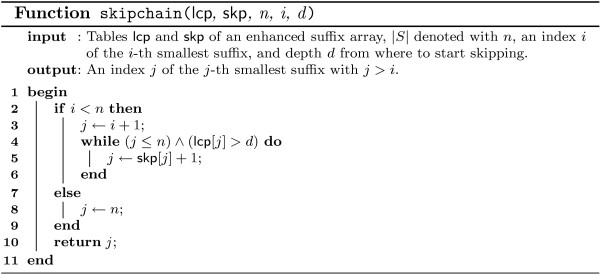
**Function skipchain of the ESAsearch algorithm**. Function skipchain computes a chain of entries in table skp to skip certain ranges of suffixes in table suf.

These case distinctions lead to the program *ESAsearch *(see Figures 3, 4).

We illustrate the ideas of algorithm *ESAsearch*, formally described above, with the following example. Let *M *be a PSSM of length *m *= 2 over alphabet A
 MathType@MTEF@5@5@+=feaafiart1ev1aaatCvAUfKttLearuWrP9MDH5MBPbIqV92AaeXatLxBI9gBamrtHrhAL1wy0L2yHvtyaeHbnfgDOvwBHrxAJfwnaebbnrfifHhDYfgasaacH8akY=wiFfYdH8Gipec8Eeeu0xXdbba9frFj0=OqFfea0dXdd9vqai=hGuQ8kuc9pgc9s8qqaq=dirpe0xb9q8qiLsFr0=vr0=vr0dc8meaabaqaciaacaGaaeqabaWaaeGaeaaakeaaimaacqWFaeFqaaa@3821@ = {*a, c*} with *M*(0, *a*) = 1, *M*(0, *c*) = 3, *M*(1, *a*) = 3, and *M*(1, *c*) = 2. For a given threshold of *th *= 6 we obtain intermediate thresholds *th*_0 _= 3 and *th*_1 _= 6. To search with *M *in the enhanced suffix array for sequence *S *= caaaaccacac as given in Figure 2, we start processing the enhanced suffix array suf top down by scoring the first suffix *S*_suf[0] _= aaaaccacac$ with *M *from left to right. For the first character of *S*_suf[0] _we obtain a score of *pfxsc*_0_(*S*_suf[0]_,*M*)= *M*(0, *a*) = 1 which is below the first intermediate threshold *th*_0_= 3. Hence we set *d*_0 _= -1 and notice that we can skip all suffixes of *S *that start with character 'a'. Further on, with a lookup in lcp[1] = 3 we find that *S*_suf[1] _and *S*_suf[0] _share a common prefix of length 3 and *d*_0 _+ 1 = -1 + 1 < lcp[1] = 3 (second case described above). The next suffix that may match *M *with *th *= 6 is *S*_suf[6] _= caaaaccacac$. Suffixes *S*_suf[1]_, *S*_suf[2]_,... *S*_suf[5] _can be skipped since they all share a common prefix with *S*_suf[0] _of at least length 1. That is, they begin all with character 'a' and would also miss the first intermediate threshold *th*_0 _= 3 when scored. We find *S*_suf[6] _by following a chain of entries in table skp: skp[1] = 2, skp[2] = 3, and skp[3] = 6. When scoring *S*_suf[6] _we compute *pfxsc*_0_(*S*_suf[6]_, *M*) = *M*(0,*c*) = 3 and *pfxsc*_1_(*S*_suf[6]_, *M*) = *M*(0, *c*) + *M*(1, *a*) = 6 and store them for reuse in *C*[0] and *C*[1]. Since *d*_6 _= 1 = *m *- 1 = 1 holds, we report suf[6] = 0 with score *sc *(*S*_suf[6]_, *M*) = *pfxsc*_1_(*S*_suf[6]_,*M*) = 6 as a matching position. With lookups in lcp[7] = 2 and lcp[8] = 3 we notice that *S*_suf[7] _and *S*_suf[8] _share a common prefix of at least two characters with *S*_suf[6]_. Hence we report suf[7] = 6 and suf[8] = 8 with score *C*[1] = 6 as further matching positions. We proceed with the scoring of *S*_suf[9]_. Since lcp[9] = 1 holds, we obtain the score for the first character 'c' from array *C *with *pfxsc*_0_(*S*_suf[9]_, *M*) = *C*[0]. After scoring the second character of *S*_suf[9]_, *pfxsc*_1_(*S*_suf[9]_, *M*) = 5 <*th*_1 _= 6 holds and we miss the second intermediate threshold and continue with the next suffix. The last two suffixes *S*_suf[10] _and *S*_suf[11] _in suf do not have to be considered since their lengths are smaller than to *m *= 2 (not counting the sentinel character $) and therefore they cannot match *M*. We end up with matching positions 0, 6, and 8 of *M *in *S *with match score 6. To find these matches, we processed the enhanced suffix array suf top down and scored suffixes from left to right, facilitating the additional information given in tables lcp and skp to avoid rescoring of characters of common prefixes of suffixes and to skip suffixes that cannot match *M *for the given threshold.

#### Analysis

The *C*_*i *_arrays can be stored in a single O
 MathType@MTEF@5@5@+=feaafiart1ev1aaatCvAUfKttLearuWrP9MDH5MBPbIqV92AaeXatLxBI9gBamrtHrhAL1wy0L2yHvtyaeHbnfgDOvwBHrxAJfwnaebbnrfifHhDYfgasaacH8akY=wiFfYdH8Gipec8Eeeu0xXdbba9frFj0=OqFfea0dXdd9vqai=hGuQ8kuc9pgc9s8qqaq=dirpe0xb9q8qiLsFr0=vr0=vr0dc8meaabaqaciaacaGaaeqabaWaaeGaeaaakeaaimaacqWFoe=taaa@383D@(*m*) space array *C *as any step *i *only needs the *C*_*i *_specific to that step. *C*_*i *_solely depends on *C*_*i*-1_, and *C*_*i*_[0..*d *- 1] = *C*_*i*-1_[0..*d *- 1] holds for a certain *d*<*m*, i.e., the first *d *entries in *C*_*i *_are known from the previous step, and thus *C *can be organized as a stack. No other space (apart from the space for the enhanced suffix array) depending on input size is required for *ESAsearch*, leading to an O
 MathType@MTEF@5@5@+=feaafiart1ev1aaatCvAUfKttLearuWrP9MDH5MBPbIqV92AaeXatLxBI9gBamrtHrhAL1wy0L2yHvtyaeHbnfgDOvwBHrxAJfwnaebbnrfifHhDYfgasaacH8akY=wiFfYdH8Gipec8Eeeu0xXdbba9frFj0=OqFfea0dXdd9vqai=hGuQ8kuc9pgc9s8qqaq=dirpe0xb9q8qiLsFr0=vr0=vr0dc8meaabaqaciaacaGaaeqabaWaaeGaeaaakeaaimaacqWFoe=taaa@383D@(*m*) space complexity.

The worst case for *ESAsearch *occurs, if *th *≤ *sc*_min_(*M*) (*M *matches at each position in *S*), and no suffix of *S *shares a common prefix with any other suffix. In this case lookahead scoring does not give any speedup and every suffix must be read up to depth to *m*, leading to an O
 MathType@MTEF@5@5@+=feaafiart1ev1aaatCvAUfKttLearuWrP9MDH5MBPbIqV92AaeXatLxBI9gBamrtHrhAL1wy0L2yHvtyaeHbnfgDOvwBHrxAJfwnaebbnrfifHhDYfgasaacH8akY=wiFfYdH8Gipec8Eeeu0xXdbba9frFj0=OqFfea0dXdd9vqai=hGuQ8kuc9pgc9s8qqaq=dirpe0xb9q8qiLsFr0=vr0=vr0dc8meaabaqaciaacaGaaeqabaWaaeGaeaaakeaaimaacqWFoe=taaa@383D@(*nm*) worst case time complexity. This is not worse but also not better than the complexity for *LAsearch*. Next we show that, *independent *of the chosen threshold *th*, the overall worst case running time boundary for *ESAsearch *drops to O
 MathType@MTEF@5@5@+=feaafiart1ev1aaatCvAUfKttLearuWrP9MDH5MBPbIqV92AaeXatLxBI9gBamrtHrhAL1wy0L2yHvtyaeHbnfgDOvwBHrxAJfwnaebbnrfifHhDYfgasaacH8akY=wiFfYdH8Gipec8Eeeu0xXdbba9frFj0=OqFfea0dXdd9vqai=hGuQ8kuc9pgc9s8qqaq=dirpe0xb9q8qiLsFr0=vr0=vr0dc8meaabaqaciaacaGaaeqabaWaaeGaeaaakeaaimaacqWFoe=taaa@383D@(*n *+ *m*) under the assumption that

*n *≥ |A
 MathType@MTEF@5@5@+=feaafiart1ev1aaatCvAUfKttLearuWrP9MDH5MBPbIqV92AaeXatLxBI9gBamrtHrhAL1wy0L2yHvtyaeHbnfgDOvwBHrxAJfwnaebbnrfifHhDYfgasaacH8akY=wiFfYdH8Gipec8Eeeu0xXdbba9frFj0=OqFfea0dXdd9vqai=hGuQ8kuc9pgc9s8qqaq=dirpe0xb9q8qiLsFr0=vr0=vr0dc8meaabaqaciaacaGaaeqabaWaaeGaeaaakeaaimaacqWFaeFqaaa@3821@|^*m *^+ *m *- 1     (1)

holds.

The shorter the common prefixes of the neighboring suffixes, the slower *ESAsearch *runs. Thus to analyze the worst case, we have to consider sequences containing as many different substrings of some length *q *as possible. Observe that a sequence can contain at most |A
 MathType@MTEF@5@5@+=feaafiart1ev1aaatCvAUfKttLearuWrP9MDH5MBPbIqV92AaeXatLxBI9gBamrtHrhAL1wy0L2yHvtyaeHbnfgDOvwBHrxAJfwnaebbnrfifHhDYfgasaacH8akY=wiFfYdH8Gipec8Eeeu0xXdbba9frFj0=OqFfea0dXdd9vqai=hGuQ8kuc9pgc9s8qqaq=dirpe0xb9q8qiLsFr0=vr0=vr0dc8meaabaqaciaacaGaaeqabaWaaeGaeaaakeaaimaacqWFaeFqaaa@3821@|^*q *^different substrings of length *q *> 0, independent of its length. To analyze the behavior of *ESAsearch *on such a sequence, we introduce the concept of suffix-intervals on enhanced suffix arrays, similar to lcp-intervals as used in [18].

**Definition 1 **An interval [*i*, *j*], 0 ≤ *i *≤ *j *≤ *n*, is a *suffix-interval *with offset ℓ ∈ {0,..., *n*}, or ℓ-*suffix-interval*, denoted ℓ-[*i*, *j*], if the following three conditions hold:

1. lcp[*i*] < ℓ

2. lcp[*j *+ 1] < ℓ

3. lcp[*k*] ≥ ℓ for all *k *∈ {*x *| *i *+ 1 ≤ *x *≤ *j*}

An *lcp-interval*, or ℓ-interval, with lcp-value ℓ ∈ {0,..., *n*} is a suffix-interval ℓ - [*i*, *j*] with *i *<*j *and lcp[*k*] = ℓ for at least one *k *∈ {*i *+ 1,..., *j*}.

Every lcp-interval ℓ - [*i*, *j*] of an enhanced suffix array for text *S *corresponds to an internal node *v *in a suffix tree for *S*, and the length of the string spelled out by the edge labels on the path from the root node to *v *is equal to ℓ. Leaves are represented as singleton intervals, ℓ - [*i*, *j*] with *i *= *j*. We say that suffix-interval ℓ - [*i*, *j*] embeds suffix-interval ℓ^+ ^- [*k*, *l*], if and only if ℓ^+ ^> ℓ, *i *≤ *k *<*l *≤ *j*, and if there is no suffix-interval ℓ' - [*r*, *s*] with ℓ < ℓ' < ℓ^+ ^and *i *≤ *r *≤ *k *<*l *≤ *s *≤ *j*. As an example for ℓ-suffix-intervals, consider the enhanced suffix array given in Figure 2. [0, 5] is a 1-suffix-interval, because lcp[0] = 0 < 1, lcp[5 + 1] = 0 < 1, and lcp[*k*] ≥ 1, for all *k*, 1 ≤ *k *≤ 5. Suffix-interval 2-[3,5] is embedded in 1-[0,5], but 3-[0,1] is not. Consider an enhanced suffix array of a sequence which contains all possible substrings of length *q*. There are |A
 MathType@MTEF@5@5@+=feaafiart1ev1aaatCvAUfKttLearuWrP9MDH5MBPbIqV92AaeXatLxBI9gBamrtHrhAL1wy0L2yHvtyaeHbnfgDOvwBHrxAJfwnaebbnrfifHhDYfgasaacH8akY=wiFfYdH8Gipec8Eeeu0xXdbba9frFj0=OqFfea0dXdd9vqai=hGuQ8kuc9pgc9s8qqaq=dirpe0xb9q8qiLsFr0=vr0=vr0dc8meaabaqaciaacaGaaeqabaWaaeGaeaaakeaaimaacqWFaeFqaaa@3821@| 1-suffix-intervals, |A
 MathType@MTEF@5@5@+=feaafiart1ev1aaatCvAUfKttLearuWrP9MDH5MBPbIqV92AaeXatLxBI9gBamrtHrhAL1wy0L2yHvtyaeHbnfgDOvwBHrxAJfwnaebbnrfifHhDYfgasaacH8akY=wiFfYdH8Gipec8Eeeu0xXdbba9frFj0=OqFfea0dXdd9vqai=hGuQ8kuc9pgc9s8qqaq=dirpe0xb9q8qiLsFr0=vr0=vr0dc8meaabaqaciaacaGaaeqabaWaaeGaeaaakeaaimaacqWFaeFqaaa@3821@|^2 ^2-suffix-intervals, and so on. Consequently, up to depth *q*, there are a total of

Eq=∑i=1q|A|i=|A|q+1−|A||A|−1     (2)
 MathType@MTEF@5@5@+=feaafiart1ev1aaatCvAUfKttLearuWrP9MDH5MBPbIqV92AaeXatLxBI9gBamrtHrhAL1wy0L2yHvtyaeHbnfgDOvwBHrxAJfwnaebbnrfifHhDYfgasaacH8akY=wiFfYdH8Gipec8Eeeu0xXdbba9frFj0=OqFfea0dXdd9vqai=hGuQ8kuc9pgc9s8qqaq=dirpe0xb9q8qiLsFr0=vr0=vr0dc8meaabaqaciaacaGaaeqabaWaaeGaeaaakeaacqWGfbqrdaWgaaWcbaGaemyCaehabeaakiabg2da9maaqahabaWaaqWaaeaaimaacqWFaeFqaiaawEa7caGLiWoadaahaaWcbeqaaiabdMgaPbaaaeaacqWGPbqAcqGH9aqpcqaIXaqmaeaacqWGXbqCa0GaeyyeIuoakiabg2da9maalaaabaWaaqWaaeaacqWFaeFqaiaawEa7caGLiWoadaahaaWcbeqaaiabdghaXjabgUcaRiabigdaXaaakiabgkHiTmaaemaabaGae8haXheacaGLhWUaayjcSdaabaWaaqWaaeaacqWFaeFqaiaawEa7caGLiWoacqGHsislcqaIXaqmaaGaaCzcaiaaxMaadaqadaqaaiabikdaYaGaayjkaiaawMcaaaaa@6120@

ℓ-suffix-intervals (1 ≤ ℓ ≤ *q*). This corresponds to the number of internal nodes and leaves in a suffix tree, which is atomic up to at least depth *q *under our assumptions.

Since we are considering sequences that contain all possible substrings of length *q*, there are |A
 MathType@MTEF@5@5@+=feaafiart1ev1aaatCvAUfKttLearuWrP9MDH5MBPbIqV92AaeXatLxBI9gBamrtHrhAL1wy0L2yHvtyaeHbnfgDOvwBHrxAJfwnaebbnrfifHhDYfgasaacH8akY=wiFfYdH8Gipec8Eeeu0xXdbba9frFj0=OqFfea0dXdd9vqai=hGuQ8kuc9pgc9s8qqaq=dirpe0xb9q8qiLsFr0=vr0=vr0dc8meaabaqaciaacaGaaeqabaWaaeGaeaaakeaaimaacqWFaeFqaaa@3821@|^*d*^*d*-suffix-intervals at any depth *d*, 1 ≤ *d *≤ *q*. Let *d*-[*i*, *j*] be a *d*-suffix-interval. We know that *pfxsc*_*d *_(*v*_*i*_, *M*) is a partial sum of *pfxsc*_*q *_(*v*_*i*_, *M*), and because *v*_*i*_[0.. *d *- 1] = *v*_*i *+ 1 _[0..*d *- 1] = ... = *v*_*j *_[0.. *d *- 1], *pfxsc*_*d*_(*v*_*i*_, *M*) is also a partial sum of *pfxsc*_*q *_(*v*_*k*_, *M*) for *i *≤ *k *≤ *j*. That is, after *ESAsearch *has calculated *pfxsc*_*d*_(*v*_*i*_, *M*) at depth *d*, at any suffix-interval (*d *+ 1) - [*r*, *s*] embedded in *d*-[*i*, *j*] it suffices to only calculate the "rest" of *pfxsc*_*q *_(*v*_*k*_, *M*). At any depth *d*, the algorithm calculates *pfxsc*_*d*+1 _(*v*_*r*_, *M*) = *pfxsc*_*d *_(*v*_*i*_, *M*) + *M*(*d*, *v*_*r*_[*d*]), meaning that all prefix scores at depth *d *+ 1 in a *d*-suffix-interval can be computed from the prefix scores at depth *d *by |A
 MathType@MTEF@5@5@+=feaafiart1ev1aaatCvAUfKttLearuWrP9MDH5MBPbIqV92AaeXatLxBI9gBamrtHrhAL1wy0L2yHvtyaeHbnfgDOvwBHrxAJfwnaebbnrfifHhDYfgasaacH8akY=wiFfYdH8Gipec8Eeeu0xXdbba9frFj0=OqFfea0dXdd9vqai=hGuQ8kuc9pgc9s8qqaq=dirpe0xb9q8qiLsFr0=vr0=vr0dc8meaabaqaciaacaGaaeqabaWaaeGaeaaakeaaimaacqWFaeFqaaa@3821@| matrix look-ups and additions as there are |A
 MathType@MTEF@5@5@+=feaafiart1ev1aaatCvAUfKttLearuWrP9MDH5MBPbIqV92AaeXatLxBI9gBamrtHrhAL1wy0L2yHvtyaeHbnfgDOvwBHrxAJfwnaebbnrfifHhDYfgasaacH8akY=wiFfYdH8Gipec8Eeeu0xXdbba9frFj0=OqFfea0dXdd9vqai=hGuQ8kuc9pgc9s8qqaq=dirpe0xb9q8qiLsFr0=vr0=vr0dc8meaabaqaciaacaGaaeqabaWaaeGaeaaakeaaimaacqWFaeFqaaa@3821@| embedded (*d *+ 1)-suffix-intervals. There are |A
 MathType@MTEF@5@5@+=feaafiart1ev1aaatCvAUfKttLearuWrP9MDH5MBPbIqV92AaeXatLxBI9gBamrtHrhAL1wy0L2yHvtyaeHbnfgDOvwBHrxAJfwnaebbnrfifHhDYfgasaacH8akY=wiFfYdH8Gipec8Eeeu0xXdbba9frFj0=OqFfea0dXdd9vqai=hGuQ8kuc9pgc9s8qqaq=dirpe0xb9q8qiLsFr0=vr0=vr0dc8meaabaqaciaacaGaaeqabaWaaeGaeaaakeaaimaacqWFaeFqaaa@3821@|^*d*^* d*-suffix-intervals at depth *d*. Hence, it takes *ESAsearch *a total of |A
 MathType@MTEF@5@5@+=feaafiart1ev1aaatCvAUfKttLearuWrP9MDH5MBPbIqV92AaeXatLxBI9gBamrtHrhAL1wy0L2yHvtyaeHbnfgDOvwBHrxAJfwnaebbnrfifHhDYfgasaacH8akY=wiFfYdH8Gipec8Eeeu0xXdbba9frFj0=OqFfea0dXdd9vqai=hGuQ8kuc9pgc9s8qqaq=dirpe0xb9q8qiLsFr0=vr0=vr0dc8meaabaqaciaacaGaaeqabaWaaeGaeaaakeaaimaacqWFaeFqaaa@3821@|^*d*^·|A
 MathType@MTEF@5@5@+=feaafiart1ev1aaatCvAUfKttLearuWrP9MDH5MBPbIqV92AaeXatLxBI9gBamrtHrhAL1wy0L2yHvtyaeHbnfgDOvwBHrxAJfwnaebbnrfifHhDYfgasaacH8akY=wiFfYdH8Gipec8Eeeu0xXdbba9frFj0=OqFfea0dXdd9vqai=hGuQ8kuc9pgc9s8qqaq=dirpe0xb9q8qiLsFr0=vr0=vr0dc8meaabaqaciaacaGaaeqabaWaaeGaeaaakeaaimaacqWFaeFqaaa@3821@| matrix look-ups and additions to advance from depth *d *to *d *+ 1, and thus we conclude that the algorithm requires a total of O
 MathType@MTEF@5@5@+=feaafiart1ev1aaatCvAUfKttLearuWrP9MDH5MBPbIqV92AaeXatLxBI9gBamrtHrhAL1wy0L2yHvtyaeHbnfgDOvwBHrxAJfwnaebbnrfifHhDYfgasaacH8akY=wiFfYdH8Gipec8Eeeu0xXdbba9frFj0=OqFfea0dXdd9vqai=hGuQ8kuc9pgc9s8qqaq=dirpe0xb9q8qiLsFr0=vr0=vr0dc8meaabaqaciaacaGaaeqabaWaaeGaeaaakeaaimaacqWFoe=taaa@383D@(*E*_*q*_) operations to compute all scores for all substrings of length *q*.

Suppose that *ESAsearch *has read suffix *v*_*i *_in some step up to depth *q *- 1 such that character *v*_*i*_[*q *- 1] is the last one read. If lcp[*i *+ 1] ≥ *q *holds, then the algorithm has found a suffix-interval *q*-[*i*, *j*] with a yet unknown right boundary *j*, otherwise *j *= *i*. *ESAsearch *reports all suf[*k*] with *k *∈ [*i*, *j*] as matching positions by scanning over table lcp starting at position *i *until lcp[*k*] < lcp[*i*] (such that it finds *j *= *k *- 1), and continues with suffix *v*_*k *_at depth lcp[*k*]. Hence processing such a suffix-interval requires one matrix look-up and addition to compute the score, and *j *- *i *+ 1 steps to report all matches and find suffix *v*_*k*_. Since suffix-intervals do not overlap, the total length of all suffix-intervals at depth *q *can be at most *n*, so the total time spent on reporting matches is bounded by *n*.

There are three cases to consider when determining the time required for calculating the match scores for a PSSM of length *m*. Let *p *: = *m *- *q*.

1. If *p *= 0 (⇒ *m *= *q*), then the time required to calculate all match scores is in O
 MathType@MTEF@5@5@+=feaafiart1ev1aaatCvAUfKttLearuWrP9MDH5MBPbIqV92AaeXatLxBI9gBamrtHrhAL1wy0L2yHvtyaeHbnfgDOvwBHrxAJfwnaebbnrfifHhDYfgasaacH8akY=wiFfYdH8Gipec8Eeeu0xXdbba9frFj0=OqFfea0dXdd9vqai=hGuQ8kuc9pgc9s8qqaq=dirpe0xb9q8qiLsFr0=vr0=vr0dc8meaabaqaciaacaGaaeqabaWaaeGaeaaakeaaimaacqWFoe=taaa@383D@(*E*_*q*_) as discussed above.

2. If *p *< 0 (⇒ *m*<*q*), then none of the *m*-suffix-intervals are singletons since we assumed that the sequence under consideration contains all possible substrings of length *q*, i.e., there must be suffixes sharing a common prefix of length *m*, and the time required to calculate all match scores is in O
 MathType@MTEF@5@5@+=feaafiart1ev1aaatCvAUfKttLearuWrP9MDH5MBPbIqV92AaeXatLxBI9gBamrtHrhAL1wy0L2yHvtyaeHbnfgDOvwBHrxAJfwnaebbnrfifHhDYfgasaacH8akY=wiFfYdH8Gipec8Eeeu0xXdbba9frFj0=OqFfea0dXdd9vqai=hGuQ8kuc9pgc9s8qqaq=dirpe0xb9q8qiLsFr0=vr0=vr0dc8meaabaqaciaacaGaaeqabaWaaeGaeaaakeaaimaacqWFoe=taaa@383D@(*E*_*m*_).

3. If *p *> 0 (⇒ *m *> *q*), then every *m*-suffix-interval can be a singleton, and all prefix scores for the PSSM prefix of length *q *are calculated in O
 MathType@MTEF@5@5@+=feaafiart1ev1aaatCvAUfKttLearuWrP9MDH5MBPbIqV92AaeXatLxBI9gBamrtHrhAL1wy0L2yHvtyaeHbnfgDOvwBHrxAJfwnaebbnrfifHhDYfgasaacH8akY=wiFfYdH8Gipec8Eeeu0xXdbba9frFj0=OqFfea0dXdd9vqai=hGuQ8kuc9pgc9s8qqaq=dirpe0xb9q8qiLsFr0=vr0=vr0dc8meaabaqaciaacaGaaeqabaWaaeGaeaaakeaaimaacqWFoe=taaa@383D@(*E*_*q*_) time. However, the remaining scores for the pending substrings of length *p *must be computed for *every *suffix longer than *q*, taking O
 MathType@MTEF@5@5@+=feaafiart1ev1aaatCvAUfKttLearuWrP9MDH5MBPbIqV92AaeXatLxBI9gBamrtHrhAL1wy0L2yHvtyaeHbnfgDOvwBHrxAJfwnaebbnrfifHhDYfgasaacH8akY=wiFfYdH8Gipec8Eeeu0xXdbba9frFj0=OqFfea0dXdd9vqai=hGuQ8kuc9pgc9s8qqaq=dirpe0xb9q8qiLsFr0=vr0=vr0dc8meaabaqaciaacaGaaeqabaWaaeGaeaaakeaaimaacqWFoe=taaa@383D@(*np*) additional time, and leading to a total O
 MathType@MTEF@5@5@+=feaafiart1ev1aaatCvAUfKttLearuWrP9MDH5MBPbIqV92AaeXatLxBI9gBamrtHrhAL1wy0L2yHvtyaeHbnfgDOvwBHrxAJfwnaebbnrfifHhDYfgasaacH8akY=wiFfYdH8Gipec8Eeeu0xXdbba9frFj0=OqFfea0dXdd9vqai=hGuQ8kuc9pgc9s8qqaq=dirpe0xb9q8qiLsFr0=vr0=vr0dc8meaabaqaciaacaGaaeqabaWaaeGaeaaakeaaimaacqWFoe=taaa@383D@(*E*_*q *_+ *np*) worst case time complexity for computing all match scores.

Note that a text containing |A
 MathType@MTEF@5@5@+=feaafiart1ev1aaatCvAUfKttLearuWrP9MDH5MBPbIqV92AaeXatLxBI9gBamrtHrhAL1wy0L2yHvtyaeHbnfgDOvwBHrxAJfwnaebbnrfifHhDYfgasaacH8akY=wiFfYdH8Gipec8Eeeu0xXdbba9frFj0=OqFfea0dXdd9vqai=hGuQ8kuc9pgc9s8qqaq=dirpe0xb9q8qiLsFr0=vr0=vr0dc8meaabaqaciaacaGaaeqabaWaaeGaeaaakeaaimaacqWFaeFqaaa@3821@|^*q *^different substrings must have a certain length, which must be at least |A
 MathType@MTEF@5@5@+=feaafiart1ev1aaatCvAUfKttLearuWrP9MDH5MBPbIqV92AaeXatLxBI9gBamrtHrhAL1wy0L2yHvtyaeHbnfgDOvwBHrxAJfwnaebbnrfifHhDYfgasaacH8akY=wiFfYdH8Gipec8Eeeu0xXdbba9frFj0=OqFfea0dXdd9vqai=hGuQ8kuc9pgc9s8qqaq=dirpe0xb9q8qiLsFr0=vr0=vr0dc8meaabaqaciaacaGaaeqabaWaaeGaeaaakeaaimaacqWFaeFqaaa@3821@|^*q*^. In fact, a minimum length text that contains all strings of length *q *has length *n *= |A
 MathType@MTEF@5@5@+=feaafiart1ev1aaatCvAUfKttLearuWrP9MDH5MBPbIqV92AaeXatLxBI9gBamrtHrhAL1wy0L2yHvtyaeHbnfgDOvwBHrxAJfwnaebbnrfifHhDYfgasaacH8akY=wiFfYdH8Gipec8Eeeu0xXdbba9frFj0=OqFfea0dXdd9vqai=hGuQ8kuc9pgc9s8qqaq=dirpe0xb9q8qiLsFr0=vr0=vr0dc8meaabaqaciaacaGaaeqabaWaaeGaeaaakeaaimaacqWFaeFqaaa@3821@|^*q *^+ *q *- 1. It represents a *de Bruijn sequence*[28] without wrap-around, i.e., a *de Bruijn *sequence with its first *q *- 1 characters concatenated to its end. Since a *de Bruijn *sequence without wrap-around represents the minimum length worst case, we infer from Equation (2) that *E*_*q *_∈ O
 MathType@MTEF@5@5@+=feaafiart1ev1aaatCvAUfKttLearuWrP9MDH5MBPbIqV92AaeXatLxBI9gBamrtHrhAL1wy0L2yHvtyaeHbnfgDOvwBHrxAJfwnaebbnrfifHhDYfgasaacH8akY=wiFfYdH8Gipec8Eeeu0xXdbba9frFj0=OqFfea0dXdd9vqai=hGuQ8kuc9pgc9s8qqaq=dirpe0xb9q8qiLsFr0=vr0=vr0dc8meaabaqaciaacaGaaeqabaWaaeGaeaaakeaaimaacqWFoe=taaa@383D@(*n*). Hence, if *m *= *q*, then it takes O
 MathType@MTEF@5@5@+=feaafiart1ev1aaatCvAUfKttLearuWrP9MDH5MBPbIqV92AaeXatLxBI9gBamrtHrhAL1wy0L2yHvtyaeHbnfgDOvwBHrxAJfwnaebbnrfifHhDYfgasaacH8akY=wiFfYdH8Gipec8Eeeu0xXdbba9frFj0=OqFfea0dXdd9vqai=hGuQ8kuc9pgc9s8qqaq=dirpe0xb9q8qiLsFr0=vr0=vr0dc8meaabaqaciaacaGaaeqabaWaaeGaeaaakeaaimaacqWFoe=taaa@383D@(*n*) time to calculate all match scores. If *m *<*q*, then *E*_*m *_<*E*_*q *_and thus it takes sublinear time. If *m *> *q*, it takes O
 MathType@MTEF@5@5@+=feaafiart1ev1aaatCvAUfKttLearuWrP9MDH5MBPbIqV92AaeXatLxBI9gBamrtHrhAL1wy0L2yHvtyaeHbnfgDOvwBHrxAJfwnaebbnrfifHhDYfgasaacH8akY=wiFfYdH8Gipec8Eeeu0xXdbba9frFj0=OqFfea0dXdd9vqai=hGuQ8kuc9pgc9s8qqaq=dirpe0xb9q8qiLsFr0=vr0=vr0dc8meaabaqaciaacaGaaeqabaWaaeGaeaaakeaaimaacqWFoe=taaa@383D@(*n *+ *np*) time.

We summarize the worst case running time of *ESAsearch *for preprocessing a PSSM *M *of length *m*, searching with *M*, and reporting all matches with their match scores, as

O
 MathType@MTEF@5@5@+=feaafiart1ev1aaatCvAUfKttLearuWrP9MDH5MBPbIqV92AaeXatLxBI9gBamrtHrhAL1wy0L2yHvtyaeHbnfgDOvwBHrxAJfwnaebbnrfifHhDYfgasaacH8akY=wiFfYdH8Gipec8Eeeu0xXdbba9frFj0=OqFfea0dXdd9vqai=hGuQ8kuc9pgc9s8qqaq=dirpe0xb9q8qiLsFr0=vr0=vr0dc8meaabaqaciaacaGaaeqabaWaaeGaeaaakeaaimaacqWFoe=taaa@383D@(*n *+ *n*·max {0, *p*} + *m*).

Hence, the worst case running time is O
 MathType@MTEF@5@5@+=feaafiart1ev1aaatCvAUfKttLearuWrP9MDH5MBPbIqV92AaeXatLxBI9gBamrtHrhAL1wy0L2yHvtyaeHbnfgDOvwBHrxAJfwnaebbnrfifHhDYfgasaacH8akY=wiFfYdH8Gipec8Eeeu0xXdbba9frFj0=OqFfea0dXdd9vqai=hGuQ8kuc9pgc9s8qqaq=dirpe0xb9q8qiLsFr0=vr0=vr0dc8meaabaqaciaacaGaaeqabaWaaeGaeaaakeaaimaacqWFoe=taaa@383D@(*n *+ *m*) for *p *≤ 0, implying that this time complexity holds for any PSSM of length *m *and threshold on any text of length *n *≥ |A
 MathType@MTEF@5@5@+=feaafiart1ev1aaatCvAUfKttLearuWrP9MDH5MBPbIqV92AaeXatLxBI9gBamrtHrhAL1wy0L2yHvtyaeHbnfgDOvwBHrxAJfwnaebbnrfifHhDYfgasaacH8akY=wiFfYdH8Gipec8Eeeu0xXdbba9frFj0=OqFfea0dXdd9vqai=hGuQ8kuc9pgc9s8qqaq=dirpe0xb9q8qiLsFr0=vr0=vr0dc8meaabaqaciaacaGaaeqabaWaaeGaeaaakeaaimaacqWFaeFqaaa@3821@|^*m *^+ *m *- 1, as already stated in Inequality (1).

In practice, large numbers of suffixes can be skipped if the threshold is stringent enough, leading to a total running time *sublinear *in the size of the text, regardless of the relation between *n *and *m*. *ESAsearch *reads a suffix up to depth *m *unless an intermediate score falls short of an intermediate threshold, and skips intervals with the same or greater lcp if this happens. Right boundaries of skipped suffix-intervals are found quickly by following the chain of skip-values (see function skipchain in Figure 4). It are these jumps that make *ESAsearch *superior in terms of running time to *LAsearch *in practice. The best case is indeed O
 MathType@MTEF@5@5@+=feaafiart1ev1aaatCvAUfKttLearuWrP9MDH5MBPbIqV92AaeXatLxBI9gBamrtHrhAL1wy0L2yHvtyaeHbnfgDOvwBHrxAJfwnaebbnrfifHhDYfgasaacH8akY=wiFfYdH8Gipec8Eeeu0xXdbba9frFj0=OqFfea0dXdd9vqai=hGuQ8kuc9pgc9s8qqaq=dirpe0xb9q8qiLsFr0=vr0=vr0dc8meaabaqaciaacaGaaeqabaWaaeGaeaaakeaaimaacqWFoe=taaa@383D@(|A
 MathType@MTEF@5@5@+=feaafiart1ev1aaatCvAUfKttLearuWrP9MDH5MBPbIqV92AaeXatLxBI9gBamrtHrhAL1wy0L2yHvtyaeHbnfgDOvwBHrxAJfwnaebbnrfifHhDYfgasaacH8akY=wiFfYdH8Gipec8Eeeu0xXdbba9frFj0=OqFfea0dXdd9vqai=hGuQ8kuc9pgc9s8qqaq=dirpe0xb9q8qiLsFr0=vr0=vr0dc8meaabaqaciaacaGaaeqabaWaaeGaeaaakeaaimaacqWFaeFqaaa@3821@|) which occurs whenever there is no score in the first row of the PSSM that is greater than *th*_0_.

**Figure 5:**
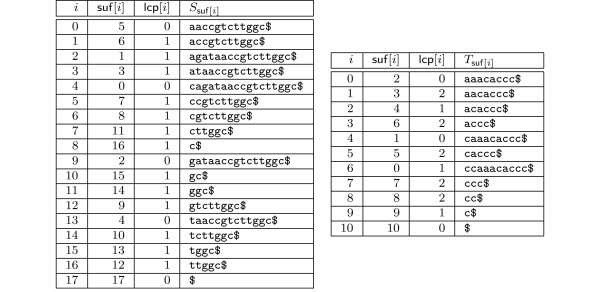
**Minimum size enhanced suffix arrays for worst case analysis**. Enhanced suffix arrays for text *S *= cagataaccgtcttggc, consisting of all strings of length *m *= 2 over an alphabet of size 4, and *T *= ccaaacaccc, consisting of all strings of length *m *= 3 over an alphabet of size 2.

See Figure 5 for examples of enhanced suffix arrays, constructed from texts *S *and *T *that consist of all strings of a certain length *m *over some alphabet. In these enhanced suffix arrays no suffix shares a prefix of length *m *with any other suffix, forcing *ESAsearch *to compute scores for each suffix. But with the intermediate scores available while processing the suffixes, it takes exactly *E*_*m *_steps to compute the scores, as can be figured out by manually applying *ESAsearch *to the depicted enhanced suffix arrays. For *S*, exactly 43−44−1=20
 MathType@MTEF@5@5@+=feaafiart1ev1aaatCvAUfKttLearuWrP9MDH5MBPbIqV92AaeXatLxBI9gBaebbnrfifHhDYfgasaacH8akY=wiFfYdH8Gipec8Eeeu0xXdbba9frFj0=OqFfea0dXdd9vqai=hGuQ8kuc9pgc9s8qqaq=dirpe0xb9q8qiLsFr0=vr0=vr0dc8meaabaqaciaacaGaaeqabaqabeGadaaakeaadaWcaaqaaiabisda0maaCaaaleqabaGaeG4mamdaaOGaeyOeI0IaeGinaqdabaGaeGinaqJaeyOeI0IaeGymaedaaiabg2da9iabikdaYiabicdaWaaa@3679@, for *T*, exactly 24−22−1=14
 MathType@MTEF@5@5@+=feaafiart1ev1aaatCvAUfKttLearuWrP9MDH5MBPbIqV92AaeXatLxBI9gBaebbnrfifHhDYfgasaacH8akY=wiFfYdH8Gipec8Eeeu0xXdbba9frFj0=OqFfea0dXdd9vqai=hGuQ8kuc9pgc9s8qqaq=dirpe0xb9q8qiLsFr0=vr0=vr0dc8meaabaqaciaacaGaaeqabaqabeGadaaakeaadaWcaaqaaiabikdaYmaaCaaaleqabaGaeGinaqdaaOGaeyOeI0IaeGOmaidabaGaeGOmaiJaeyOeI0IaeGymaedaaiabg2da9iabigdaXiabisda0aaa@3675@ operations are needed to compute *all *|A
 MathType@MTEF@5@5@+=feaafiart1ev1aaatCvAUfKttLearuWrP9MDH5MBPbIqV92AaeXatLxBI9gBamrtHrhAL1wy0L2yHvtyaeHbnfgDOvwBHrxAJfwnaebbnrfifHhDYfgasaacH8akY=wiFfYdH8Gipec8Eeeu0xXdbba9frFj0=OqFfea0dXdd9vqai=hGuQ8kuc9pgc9s8qqaq=dirpe0xb9q8qiLsFr0=vr0=vr0dc8meaabaqaciaacaGaaeqabaWaaeGaeaaakeaaimaacqWFaeFqaaa@3821@|^*m *^≤ *n *- *m *+ 1 possible scores (and to find all matches since *S *and *T *are both *de Bruijn *sequences without wrap-around). Only a single match is reported per matching substring, leading to *E*_*m *_∈ O
 MathType@MTEF@5@5@+=feaafiart1ev1aaatCvAUfKttLearuWrP9MDH5MBPbIqV92AaeXatLxBI9gBamrtHrhAL1wy0L2yHvtyaeHbnfgDOvwBHrxAJfwnaebbnrfifHhDYfgasaacH8akY=wiFfYdH8Gipec8Eeeu0xXdbba9frFj0=OqFfea0dXdd9vqai=hGuQ8kuc9pgc9s8qqaq=dirpe0xb9q8qiLsFr0=vr0=vr0dc8meaabaqaciaacaGaaeqabaWaaeGaeaaakeaaimaacqWFoe=taaa@383D@(*n*) operations to be performed during the search phase.

### Performance improvements via alphabet transformations

Inequality (1) provides the necessary condition for O
 MathType@MTEF@5@5@+=feaafiart1ev1aaatCvAUfKttLearuWrP9MDH5MBPbIqV92AaeXatLxBI9gBamrtHrhAL1wy0L2yHvtyaeHbnfgDOvwBHrxAJfwnaebbnrfifHhDYfgasaacH8akY=wiFfYdH8Gipec8Eeeu0xXdbba9frFj0=OqFfea0dXdd9vqai=hGuQ8kuc9pgc9s8qqaq=dirpe0xb9q8qiLsFr0=vr0=vr0dc8meaabaqaciaacaGaaeqabaWaaeGaeaaakeaaimaacqWFoe=taaa@383D@(*n *+ *m*) worst case running time. We now assume that *m *in Inequality (1) identifies not the length of a PSSM, but the threshold dependent *expected reading depth *for some PSSM. We denote this expected depth by m*(*th*) ≤ *m *and continue denoting the PSSM's length by *m*. As seen before, for PSSMs with length *m*, such that *p *= *m *- m*(*th*), the worst case running time is O
 MathType@MTEF@5@5@+=feaafiart1ev1aaatCvAUfKttLearuWrP9MDH5MBPbIqV92AaeXatLxBI9gBamrtHrhAL1wy0L2yHvtyaeHbnfgDOvwBHrxAJfwnaebbnrfifHhDYfgasaacH8akY=wiFfYdH8Gipec8Eeeu0xXdbba9frFj0=OqFfea0dXdd9vqai=hGuQ8kuc9pgc9s8qqaq=dirpe0xb9q8qiLsFr0=vr0=vr0dc8meaabaqaciaacaGaaeqabaWaaeGaeaaakeaaimaacqWFoe=taaa@383D@(*n *+ *n*·max {0, *p*} + *m*), but the expected running time is O
 MathType@MTEF@5@5@+=feaafiart1ev1aaatCvAUfKttLearuWrP9MDH5MBPbIqV92AaeXatLxBI9gBamrtHrhAL1wy0L2yHvtyaeHbnfgDOvwBHrxAJfwnaebbnrfifHhDYfgasaacH8akY=wiFfYdH8Gipec8Eeeu0xXdbba9frFj0=OqFfea0dXdd9vqai=hGuQ8kuc9pgc9s8qqaq=dirpe0xb9q8qiLsFr0=vr0=vr0dc8meaabaqaciaacaGaaeqabaWaaeGaeaaakeaaimaacqWFoe=taaa@383D@(*n *+ *m*), as on average we expect *p *≤ 0. Inequality (1) with *m *substituted by m*(*th*) implies log⁡|A|
 MathType@MTEF@5@5@+=feaafiart1ev1aaatCvAUfKttLearuWrP9MDH5MBPbIqV92AaeXatLxBI9gBamrtHrhAL1wy0L2yHvtyaeHbnfgDOvwBHrxAJfwnaebbnrfifHhDYfgasaacH8akY=wiFfYdH8Gipec8Eeeu0xXdbba9frFj0=OqFfea0dXdd9vqai=hGuQ8kuc9pgc9s8qqaq=dirpe0xb9q8qiLsFr0=vr0=vr0dc8meaabaqaciaacaGaaeqabaWaaeGaeaaakeaacyGGSbaBcqGGVbWBcqGGNbWzdaWgaaWcbaWaaqWaaeaaimaacqWFaeFqaiaawEa7caGLiWoaaeqaaaaa@3F8D@ (*n*) ≥ m*(*th*). That is, to achieve linear worst case running time for the amino acid alphabet, m*(*th*) needs to be very small. For instance, if *n *= 20^7^, then the search time is guaranteed to be linear in *n *only for PSSMs with a maximum length of 7, and expected to be linear for PSSMs with expected reading depth of 7. Observe that for |A
 MathType@MTEF@5@5@+=feaafiart1ev1aaatCvAUfKttLearuWrP9MDH5MBPbIqV92AaeXatLxBI9gBamrtHrhAL1wy0L2yHvtyaeHbnfgDOvwBHrxAJfwnaebbnrfifHhDYfgasaacH8akY=wiFfYdH8Gipec8Eeeu0xXdbba9frFj0=OqFfea0dXdd9vqai=hGuQ8kuc9pgc9s8qqaq=dirpe0xb9q8qiLsFr0=vr0=vr0dc8meaabaqaciaacaGaaeqabaWaaeGaeaaakeaaimaacqWFaeFqaaa@3821@| = 4, m*(*th*) needs to be smaller or equal to 15 to achieve linear or sublinear running times. This provides the motivation to reduce the alphabet size by transforming A
 MathType@MTEF@5@5@+=feaafiart1ev1aaatCvAUfKttLearuWrP9MDH5MBPbIqV92AaeXatLxBI9gBamrtHrhAL1wy0L2yHvtyaeHbnfgDOvwBHrxAJfwnaebbnrfifHhDYfgasaacH8akY=wiFfYdH8Gipec8Eeeu0xXdbba9frFj0=OqFfea0dXdd9vqai=hGuQ8kuc9pgc9s8qqaq=dirpe0xb9q8qiLsFr0=vr0=vr0dc8meaabaqaciaacaGaaeqabaWaaeGaeaaakeaaimaacqWFaeFqaaa@3821@ into a reduced size A_
 MathType@MTEF@5@5@+=feaafiart1ev1aaatCvAUfKttLearuWrP9MDH5MBPbIqV92AaeXatLxBI9gBamrtHrhAL1wy0L2yHvtyaeHbnfgDOvwBHrxAJfwnaebbnrfifHhDYfgasaacH8akY=wiFfYdH8Gipec8Eeeu0xXdbba9frFj0=OqFfea0dXdd9vqai=hGuQ8kuc9pgc9s8qqaq=dirpe0xb9q8qiLsFr0=vr0=vr0dc8meaabaqaciaacaGaaeqabaWaaeGaeaaakeaadaqiaaqaaGWaaiab=bq8bbGaayPadaaaaa@38E3@ such that |A_
 MathType@MTEF@5@5@+=feaafiart1ev1aaatCvAUfKttLearuWrP9MDH5MBPbIqV92AaeXatLxBI9gBamrtHrhAL1wy0L2yHvtyaeHbnfgDOvwBHrxAJfwnaebbnrfifHhDYfgasaacH8akY=wiFfYdH8Gipec8Eeeu0xXdbba9frFj0=OqFfea0dXdd9vqai=hGuQ8kuc9pgc9s8qqaq=dirpe0xb9q8qiLsFr0=vr0=vr0dc8meaabaqaciaacaGaaeqabaWaaeGaeaaakeaadaqiaaqaaGWaaiab=bq8bbGaayPadaaaaa@38E3@| < |A
 MathType@MTEF@5@5@+=feaafiart1ev1aaatCvAUfKttLearuWrP9MDH5MBPbIqV92AaeXatLxBI9gBamrtHrhAL1wy0L2yHvtyaeHbnfgDOvwBHrxAJfwnaebbnrfifHhDYfgasaacH8akY=wiFfYdH8Gipec8Eeeu0xXdbba9frFj0=OqFfea0dXdd9vqai=hGuQ8kuc9pgc9s8qqaq=dirpe0xb9q8qiLsFr0=vr0=vr0dc8meaabaqaciaacaGaaeqabaWaaeGaeaaakeaaimaacqWFaeFqaaa@3821@|.

**Figure 6:**
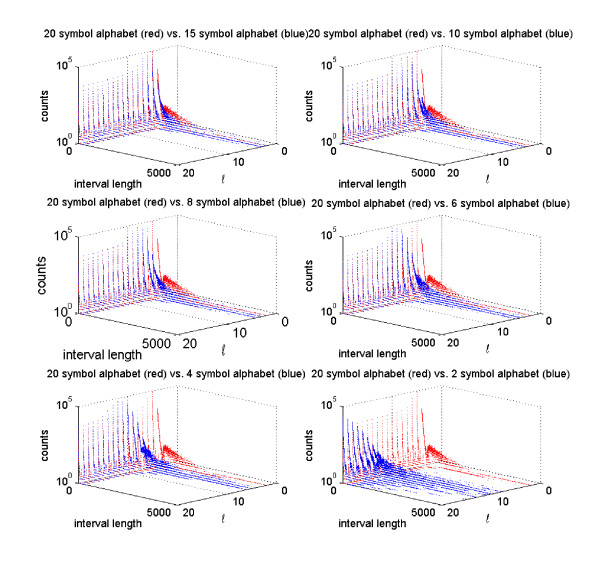
**Number of ℓ-intervals for various reduced alphabets**. Numbers of ℓ-intervals for ℓ ∈ [1, 20] of different length for various reduced alphabets. We built the enhanced suffix array with sequences from the RCSB protein data bank (PDB) (total sequence length 4,264,239 bytes). The used reduced amino acid alphabets are given in Figure 8. Note that we limited the interval lengths in the figures to 5,000 to prevent distortion.

In practice, for reasonably chosen thresholds *th*, the performance of *ESAsearch *mainly depends on the fact that often large ranges of suffixes in the enhanced suffix array can be skipped. This is always the case if we drop below an intermediate threshold while calculating a prefix' score, and if that prefix is a common prefix of other suffixes. In terms of lcp-intervals, this means that we can skip all ℓ-intervals with ℓ ≥ m*(*th*) on average. In contrast to *suffix-intervals*, whose total count is in O
 MathType@MTEF@5@5@+=feaafiart1ev1aaatCvAUfKttLearuWrP9MDH5MBPbIqV92AaeXatLxBI9gBamrtHrhAL1wy0L2yHvtyaeHbnfgDOvwBHrxAJfwnaebbnrfifHhDYfgasaacH8akY=wiFfYdH8Gipec8Eeeu0xXdbba9frFj0=OqFfea0dXdd9vqai=hGuQ8kuc9pgc9s8qqaq=dirpe0xb9q8qiLsFr0=vr0=vr0dc8meaabaqaciaacaGaaeqabaWaaeGaeaaakeaaimaacqWFoe=taaa@383D@(*n*^2^), size and number of *lcp-intervals *depend on |A
 MathType@MTEF@5@5@+=feaafiart1ev1aaatCvAUfKttLearuWrP9MDH5MBPbIqV92AaeXatLxBI9gBamrtHrhAL1wy0L2yHvtyaeHbnfgDOvwBHrxAJfwnaebbnrfifHhDYfgasaacH8akY=wiFfYdH8Gipec8Eeeu0xXdbba9frFj0=OqFfea0dXdd9vqai=hGuQ8kuc9pgc9s8qqaq=dirpe0xb9q8qiLsFr0=vr0=vr0dc8meaabaqaciaacaGaaeqabaWaaeGaeaaakeaaimaacqWFaeFqaaa@3821@|, as illustrated in Figure 6. We observe that smaller alphabet sizes imply (1) larger ℓ-intervals, and (2) an increasing number of ℓ-intervals for larger values of ℓ. Thus, by using reduced alphabets, we expect to skip larger and touch fewer lcp-intervals under the assumption that the average reading depth remains unchanged. Consequently, we expect to end up with an improved performance of *ESAsearch*. This raises the question for a proper reduction strategy for larger alphabets like the amino acid alphabet, and how this strategy can be incorporated into *ESAsearch*.

We now describe how to take advantage of reduced alphabets as fast filters in the *ESAsearch *algorithm. Let A
 MathType@MTEF@5@5@+=feaafiart1ev1aaatCvAUfKttLearuWrP9MDH5MBPbIqV92AaeXatLxBI9gBamrtHrhAL1wy0L2yHvtyaeHbnfgDOvwBHrxAJfwnaebbnrfifHhDYfgasaacH8akY=wiFfYdH8Gipec8Eeeu0xXdbba9frFj0=OqFfea0dXdd9vqai=hGuQ8kuc9pgc9s8qqaq=dirpe0xb9q8qiLsFr0=vr0=vr0dc8meaabaqaciaacaGaaeqabaWaaeGaeaaakeaaimaacqWFaeFqaaa@3821@ = {*a*_0_, *a*_1_,..., *a*_*k*_} and A_
 MathType@MTEF@5@5@+=feaafiart1ev1aaatCvAUfKttLearuWrP9MDH5MBPbIqV92AaeXatLxBI9gBamrtHrhAL1wy0L2yHvtyaeHbnfgDOvwBHrxAJfwnaebbnrfifHhDYfgasaacH8akY=wiFfYdH8Gipec8Eeeu0xXdbba9frFj0=OqFfea0dXdd9vqai=hGuQ8kuc9pgc9s8qqaq=dirpe0xb9q8qiLsFr0=vr0=vr0dc8meaabaqaciaacaGaaeqabaWaaeGaeaaakeaadaqiaaqaaGWaaiab=bq8bbGaayPadaaaaa@38E3@ = {*b*_0_, *b*_1_,..., *b*_*l*_} be two alphabets, and Φ : A
 MathType@MTEF@5@5@+=feaafiart1ev1aaatCvAUfKttLearuWrP9MDH5MBPbIqV92AaeXatLxBI9gBamrtHrhAL1wy0L2yHvtyaeHbnfgDOvwBHrxAJfwnaebbnrfifHhDYfgasaacH8akY=wiFfYdH8Gipec8Eeeu0xXdbba9frFj0=OqFfea0dXdd9vqai=hGuQ8kuc9pgc9s8qqaq=dirpe0xb9q8qiLsFr0=vr0=vr0dc8meaabaqaciaacaGaaeqabaWaaeGaeaaakeaaimaacqWFaeFqaaa@3821@ → A_
 MathType@MTEF@5@5@+=feaafiart1ev1aaatCvAUfKttLearuWrP9MDH5MBPbIqV92AaeXatLxBI9gBamrtHrhAL1wy0L2yHvtyaeHbnfgDOvwBHrxAJfwnaebbnrfifHhDYfgasaacH8akY=wiFfYdH8Gipec8Eeeu0xXdbba9frFj0=OqFfea0dXdd9vqai=hGuQ8kuc9pgc9s8qqaq=dirpe0xb9q8qiLsFr0=vr0=vr0dc8meaabaqaciaacaGaaeqabaWaaeGaeaaakeaadaqiaaqaaGWaaiab=bq8bbGaayPadaaaaa@38E3@ a surjective function that maps a character *a *∈ A
 MathType@MTEF@5@5@+=feaafiart1ev1aaatCvAUfKttLearuWrP9MDH5MBPbIqV92AaeXatLxBI9gBamrtHrhAL1wy0L2yHvtyaeHbnfgDOvwBHrxAJfwnaebbnrfifHhDYfgasaacH8akY=wiFfYdH8Gipec8Eeeu0xXdbba9frFj0=OqFfea0dXdd9vqai=hGuQ8kuc9pgc9s8qqaq=dirpe0xb9q8qiLsFr0=vr0=vr0dc8meaabaqaciaacaGaaeqabaWaaeGaeaaakeaaimaacqWFaeFqaaa@3821@ to a character *b *∈ A_
 MathType@MTEF@5@5@+=feaafiart1ev1aaatCvAUfKttLearuWrP9MDH5MBPbIqV92AaeXatLxBI9gBamrtHrhAL1wy0L2yHvtyaeHbnfgDOvwBHrxAJfwnaebbnrfifHhDYfgasaacH8akY=wiFfYdH8Gipec8Eeeu0xXdbba9frFj0=OqFfea0dXdd9vqai=hGuQ8kuc9pgc9s8qqaq=dirpe0xb9q8qiLsFr0=vr0=vr0dc8meaabaqaciaacaGaaeqabaWaaeGaeaaakeaadaqiaaqaaGWaaiab=bq8bbGaayPadaaaaa@38E3@. We call Φ^-1^(*b*) the character class corresponding to *b*. For a sequence *S *= *s*_1_*s*_2 _... *s*_*n *_∈ A
 MathType@MTEF@5@5@+=feaafiart1ev1aaatCvAUfKttLearuWrP9MDH5MBPbIqV92AaeXatLxBI9gBamrtHrhAL1wy0L2yHvtyaeHbnfgDOvwBHrxAJfwnaebbnrfifHhDYfgasaacH8akY=wiFfYdH8Gipec8Eeeu0xXdbba9frFj0=OqFfea0dXdd9vqai=hGuQ8kuc9pgc9s8qqaq=dirpe0xb9q8qiLsFr0=vr0=vr0dc8meaabaqaciaacaGaaeqabaWaaeGaeaaakeaaimaacqWFaeFqaaa@3821@^*n *^we denote the transformed sequence with S_
 MathType@MTEF@5@5@+=feaafiart1ev1aaatCvAUfKttLearuWrP9MDH5MBPbIqV92AaeXatLxBI9gBaebbnrfifHhDYfgasaacH8akY=wiFfYdH8Gipec8Eeeu0xXdbba9frFj0=OqFfea0dXdd9vqai=hGuQ8kuc9pgc9s8qqaq=dirpe0xb9q8qiLsFr0=vr0=vr0dc8meaabaqaciaacaGaaeqabaqabeGadaaakeaadaqiaaqaaiabdofatbGaayPadaaaaa@2E9D@ = Φ(*s*_1_) Φ(*s*_2_) ... Φ(*s*_*n*_) ∈ A_
 MathType@MTEF@5@5@+=feaafiart1ev1aaatCvAUfKttLearuWrP9MDH5MBPbIqV92AaeXatLxBI9gBamrtHrhAL1wy0L2yHvtyaeHbnfgDOvwBHrxAJfwnaebbnrfifHhDYfgasaacH8akY=wiFfYdH8Gipec8Eeeu0xXdbba9frFj0=OqFfea0dXdd9vqai=hGuQ8kuc9pgc9s8qqaq=dirpe0xb9q8qiLsFr0=vr0=vr0dc8meaabaqaciaacaGaaeqabaWaaeGaeaaakeaadaqiaaqaaGWaaiab=bq8bbGaayPadaaaaa@38E3@^*n*^. Along with the transformation of the sequence, we transform a PSSM such that we have a one to one relationship between the columns in the PSSM and the characters in A_
 MathType@MTEF@5@5@+=feaafiart1ev1aaatCvAUfKttLearuWrP9MDH5MBPbIqV92AaeXatLxBI9gBamrtHrhAL1wy0L2yHvtyaeHbnfgDOvwBHrxAJfwnaebbnrfifHhDYfgasaacH8akY=wiFfYdH8Gipec8Eeeu0xXdbba9frFj0=OqFfea0dXdd9vqai=hGuQ8kuc9pgc9s8qqaq=dirpe0xb9q8qiLsFr0=vr0=vr0dc8meaabaqaciaacaGaaeqabaWaaeGaeaaakeaadaqiaaqaaGWaaiab=bq8bbGaayPadaaaaa@38E3@. We define the transformed PSSM M_
 MathType@MTEF@5@5@+=feaafiart1ev1aaatCvAUfKttLearuWrP9MDH5MBPbIqV92AaeXatLxBI9gBaebbnrfifHhDYfgasaacH8akY=wiFfYdH8Gipec8Eeeu0xXdbba9frFj0=OqFfea0dXdd9vqai=hGuQ8kuc9pgc9s8qqaq=dirpe0xb9q8qiLsFr0=vr0=vr0dc8meaabaqaciaacaGaaeqabaqabeGadaaakeaadaqiaaqaaiabd2eanbGaayPadaaaaa@2E91@ of *M *as follows:

**Definition 2 **Let *M *be a PSSM of length *m *over alphabet A
 MathType@MTEF@5@5@+=feaafiart1ev1aaatCvAUfKttLearuWrP9MDH5MBPbIqV92AaeXatLxBI9gBamrtHrhAL1wy0L2yHvtyaeHbnfgDOvwBHrxAJfwnaebbnrfifHhDYfgasaacH8akY=wiFfYdH8Gipec8Eeeu0xXdbba9frFj0=OqFfea0dXdd9vqai=hGuQ8kuc9pgc9s8qqaq=dirpe0xb9q8qiLsFr0=vr0=vr0dc8meaabaqaciaacaGaaeqabaWaaeGaeaaakeaaimaacqWFaeFqaaa@3821@, and Φ : A
 MathType@MTEF@5@5@+=feaafiart1ev1aaatCvAUfKttLearuWrP9MDH5MBPbIqV92AaeXatLxBI9gBamrtHrhAL1wy0L2yHvtyaeHbnfgDOvwBHrxAJfwnaebbnrfifHhDYfgasaacH8akY=wiFfYdH8Gipec8Eeeu0xXdbba9frFj0=OqFfea0dXdd9vqai=hGuQ8kuc9pgc9s8qqaq=dirpe0xb9q8qiLsFr0=vr0=vr0dc8meaabaqaciaacaGaaeqabaWaaeGaeaaakeaaimaacqWFaeFqaaa@3821@ → A_
 MathType@MTEF@5@5@+=feaafiart1ev1aaatCvAUfKttLearuWrP9MDH5MBPbIqV92AaeXatLxBI9gBamrtHrhAL1wy0L2yHvtyaeHbnfgDOvwBHrxAJfwnaebbnrfifHhDYfgasaacH8akY=wiFfYdH8Gipec8Eeeu0xXdbba9frFj0=OqFfea0dXdd9vqai=hGuQ8kuc9pgc9s8qqaq=dirpe0xb9q8qiLsFr0=vr0=vr0dc8meaabaqaciaacaGaaeqabaWaaeGaeaaakeaadaqiaaqaaGWaaiab=bq8bbGaayPadaaaaa@38E3@ a surjective function. The transformed PSSM M_
 MathType@MTEF@5@5@+=feaafiart1ev1aaatCvAUfKttLearuWrP9MDH5MBPbIqV92AaeXatLxBI9gBaebbnrfifHhDYfgasaacH8akY=wiFfYdH8Gipec8Eeeu0xXdbba9frFj0=OqFfea0dXdd9vqai=hGuQ8kuc9pgc9s8qqaq=dirpe0xb9q8qiLsFr0=vr0=vr0dc8meaabaqaciaacaGaaeqabaqabeGadaaakeaadaqiaaqaaiabd2eanbGaayPadaaaaa@2E91@ is defined as a function M_
 MathType@MTEF@5@5@+=feaafiart1ev1aaatCvAUfKttLearuWrP9MDH5MBPbIqV92AaeXatLxBI9gBaebbnrfifHhDYfgasaacH8akY=wiFfYdH8Gipec8Eeeu0xXdbba9frFj0=OqFfea0dXdd9vqai=hGuQ8kuc9pgc9s8qqaq=dirpe0xb9q8qiLsFr0=vr0=vr0dc8meaabaqaciaacaGaaeqabaqabeGadaaakeaadaqiaaqaaiabd2eanbGaayPadaaaaa@2E91@: [0, *m *- 1] × A_
 MathType@MTEF@5@5@+=feaafiart1ev1aaatCvAUfKttLearuWrP9MDH5MBPbIqV92AaeXatLxBI9gBamrtHrhAL1wy0L2yHvtyaeHbnfgDOvwBHrxAJfwnaebbnrfifHhDYfgasaacH8akY=wiFfYdH8Gipec8Eeeu0xXdbba9frFj0=OqFfea0dXdd9vqai=hGuQ8kuc9pgc9s8qqaq=dirpe0xb9q8qiLsFr0=vr0=vr0dc8meaabaqaciaacaGaaeqabaWaaeGaeaaakeaadaqiaaqaaGWaaiab=bq8bbGaayPadaaaaa@38E3@ → ℝ with

M_
 MathType@MTEF@5@5@+=feaafiart1ev1aaatCvAUfKttLearuWrP9MDH5MBPbIqV92AaeXatLxBI9gBaebbnrfifHhDYfgasaacH8akY=wiFfYdH8Gipec8Eeeu0xXdbba9frFj0=OqFfea0dXdd9vqai=hGuQ8kuc9pgc9s8qqaq=dirpe0xb9q8qiLsFr0=vr0=vr0dc8meaabaqaciaacaGaaeqabaqabeGadaaakeaadaqiaaqaaiabd2eanbGaayPadaaaaa@2E91@ (*i*, *b*): = max {*M*(*i*, *a*) | *a *∈ Φ^-1 ^(*b*)}.     (3)

**Figure 7:**
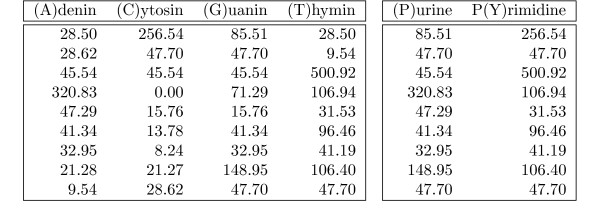
**PSSM alphabet transformation**. In the left PSSM *M *we used the normal four letter nucleotide alphabet A
 MathType@MTEF@5@5@+=feaafiart1ev1aaatCvAUfKttLearuWrP9MDH5MBPbIqV92AaeXatLxBI9gBamrtHrhAL1wy0L2yHvtyaeHbnfgDOvwBHrxAJfwnaebbnrfifHhDYfgasaacH8akY=wiFfYdH8Gipec8Eeeu0xXdbba9frFj0=OqFfea0dXdd9vqai=hGuQ8kuc9pgc9s8qqaq=dirpe0xb9q8qiLsFr0=vr0=vr0dc8meaabaqaciaacaGaaeqabaWaaeGaeaaakeaaimaacqWFaeFqaaa@3821@ = {*A*, *C*, *G*, *T*} to describe a transcription factor binding site found in Hox A3 gene promoters. In the right PSSM M_
 MathType@MTEF@5@5@+=feaafiart1ev1aaatCvAUfKttLearuWrP9MDH5MBPbIqV92AaeXatLxBI9gBaebbnrfifHhDYfgasaacH8akY=wiFfYdH8Gipec8Eeeu0xXdbba9frFj0=OqFfea0dXdd9vqai=hGuQ8kuc9pgc9s8qqaq=dirpe0xb9q8qiLsFr0=vr0=vr0dc8meaabaqaciaacaGaaeqabaqabeGadaaakeaadaqiaaqaaiabd2eanbGaayPadaaaaa@2E91@ we used a reduced two letter alphabet A_
 MathType@MTEF@5@5@+=feaafiart1ev1aaatCvAUfKttLearuWrP9MDH5MBPbIqV92AaeXatLxBI9gBamrtHrhAL1wy0L2yHvtyaeHbnfgDOvwBHrxAJfwnaebbnrfifHhDYfgasaacH8akY=wiFfYdH8Gipec8Eeeu0xXdbba9frFj0=OqFfea0dXdd9vqai=hGuQ8kuc9pgc9s8qqaq=dirpe0xb9q8qiLsFr0=vr0=vr0dc8meaabaqaciaacaGaaeqabaWaaeGaeaaakeaadaqiaaqaaGWaaiab=bq8bbGaayPadaaaaa@38E3@ = {*P*, *Y*} that differs only between purine (adenine or guanine) and pyrimidine (cytosine or thymine) nucleotides. Hence we have two character classes: Φ^-1^(*P*) = {*A*, *G*} and Φ^-1^(*Y*) = {*C*, *T*}. Consequently M_
 MathType@MTEF@5@5@+=feaafiart1ev1aaatCvAUfKttLearuWrP9MDH5MBPbIqV92AaeXatLxBI9gBaebbnrfifHhDYfgasaacH8akY=wiFfYdH8Gipec8Eeeu0xXdbba9frFj0=OqFfea0dXdd9vqai=hGuQ8kuc9pgc9s8qqaq=dirpe0xb9q8qiLsFr0=vr0=vr0dc8meaabaqaciaacaGaaeqabaqabeGadaaakeaadaqiaaqaaiabd2eanbGaayPadaaaaa@2E91@(*i*, *P*) = max{*M*(*i*, *a*) | *a *∈ {*A*, *G*}} and M_
 MathType@MTEF@5@5@+=feaafiart1ev1aaatCvAUfKttLearuWrP9MDH5MBPbIqV92AaeXatLxBI9gBaebbnrfifHhDYfgasaacH8akY=wiFfYdH8Gipec8Eeeu0xXdbba9frFj0=OqFfea0dXdd9vqai=hGuQ8kuc9pgc9s8qqaq=dirpe0xb9q8qiLsFr0=vr0=vr0dc8meaabaqaciaacaGaaeqabaqabeGadaaakeaadaqiaaqaaiabd2eanbGaayPadaaaaa@2E91@(*i*, *Y*) = max{*M*(*i*, *a*) | *a *∈ {*C*, *T*}} ∀*i *∈ [0, 8]

Figure 7 gives an example of the relationship between *M *and M_
 MathType@MTEF@5@5@+=feaafiart1ev1aaatCvAUfKttLearuWrP9MDH5MBPbIqV92AaeXatLxBI9gBaebbnrfifHhDYfgasaacH8akY=wiFfYdH8Gipec8Eeeu0xXdbba9frFj0=OqFfea0dXdd9vqai=hGuQ8kuc9pgc9s8qqaq=dirpe0xb9q8qiLsFr0=vr0=vr0dc8meaabaqaciaacaGaaeqabaqabeGadaaakeaadaqiaaqaaiabd2eanbGaayPadaaaaa@2E91@. S_
 MathType@MTEF@5@5@+=feaafiart1ev1aaatCvAUfKttLearuWrP9MDH5MBPbIqV92AaeXatLxBI9gBaebbnrfifHhDYfgasaacH8akY=wiFfYdH8Gipec8Eeeu0xXdbba9frFj0=OqFfea0dXdd9vqai=hGuQ8kuc9pgc9s8qqaq=dirpe0xb9q8qiLsFr0=vr0=vr0dc8meaabaqaciaacaGaaeqabaqabeGadaaakeaadaqiaaqaaiabdofatbGaayPadaaaaa@2E9D@ can be easily determined from *S *in O
 MathType@MTEF@5@5@+=feaafiart1ev1aaatCvAUfKttLearuWrP9MDH5MBPbIqV92AaeXatLxBI9gBamrtHrhAL1wy0L2yHvtyaeHbnfgDOvwBHrxAJfwnaebbnrfifHhDYfgasaacH8akY=wiFfYdH8Gipec8Eeeu0xXdbba9frFj0=OqFfea0dXdd9vqai=hGuQ8kuc9pgc9s8qqaq=dirpe0xb9q8qiLsFr0=vr0=vr0dc8meaabaqaciaacaGaaeqabaWaaeGaeaaakeaaimaacqWFoe=taaa@383D@(*n*) time, M_
 MathType@MTEF@5@5@+=feaafiart1ev1aaatCvAUfKttLearuWrP9MDH5MBPbIqV92AaeXatLxBI9gBaebbnrfifHhDYfgasaacH8akY=wiFfYdH8Gipec8Eeeu0xXdbba9frFj0=OqFfea0dXdd9vqai=hGuQ8kuc9pgc9s8qqaq=dirpe0xb9q8qiLsFr0=vr0=vr0dc8meaabaqaciaacaGaaeqabaqabeGadaaakeaadaqiaaqaaiabd2eanbGaayPadaaaaa@2E91@ in O
 MathType@MTEF@5@5@+=feaafiart1ev1aaatCvAUfKttLearuWrP9MDH5MBPbIqV92AaeXatLxBI9gBamrtHrhAL1wy0L2yHvtyaeHbnfgDOvwBHrxAJfwnaebbnrfifHhDYfgasaacH8akY=wiFfYdH8Gipec8Eeeu0xXdbba9frFj0=OqFfea0dXdd9vqai=hGuQ8kuc9pgc9s8qqaq=dirpe0xb9q8qiLsFr0=vr0=vr0dc8meaabaqaciaacaGaaeqabaWaaeGaeaaakeaaimaacqWFoe=taaa@383D@(|A
 MathType@MTEF@5@5@+=feaafiart1ev1aaatCvAUfKttLearuWrP9MDH5MBPbIqV92AaeXatLxBI9gBamrtHrhAL1wy0L2yHvtyaeHbnfgDOvwBHrxAJfwnaebbnrfifHhDYfgasaacH8akY=wiFfYdH8Gipec8Eeeu0xXdbba9frFj0=OqFfea0dXdd9vqai=hGuQ8kuc9pgc9s8qqaq=dirpe0xb9q8qiLsFr0=vr0=vr0dc8meaabaqaciaacaGaaeqabaWaaeGaeaaakeaaimaacqWFaeFqaaa@3821@|*m*) time, given *M*. We define the set of matches to *M *on *S *and M_
 MathType@MTEF@5@5@+=feaafiart1ev1aaatCvAUfKttLearuWrP9MDH5MBPbIqV92AaeXatLxBI9gBaebbnrfifHhDYfgasaacH8akY=wiFfYdH8Gipec8Eeeu0xXdbba9frFj0=OqFfea0dXdd9vqai=hGuQ8kuc9pgc9s8qqaq=dirpe0xb9q8qiLsFr0=vr0=vr0dc8meaabaqaciaacaGaaeqabaqabeGadaaakeaadaqiaaqaaiabd2eanbGaayPadaaaaa@2E91@ on S_
 MathType@MTEF@5@5@+=feaafiart1ev1aaatCvAUfKttLearuWrP9MDH5MBPbIqV92AaeXatLxBI9gBaebbnrfifHhDYfgasaacH8akY=wiFfYdH8Gipec8Eeeu0xXdbba9frFj0=OqFfea0dXdd9vqai=hGuQ8kuc9pgc9s8qqaq=dirpe0xb9q8qiLsFr0=vr0=vr0dc8meaabaqaciaacaGaaeqabaqabeGadaaakeaadaqiaaqaaiabdofatbGaayPadaaaaa@2E9D@, respectively, as

*MS *:= {*j *∈ [0, *n *- *m*] | *sc *(*S*[*j*..*j *+ *m *- 1], *M*) ≥ *th*}

MS_
 MathType@MTEF@5@5@+=feaafiart1ev1aaatCvAUfKttLearuWrP9MDH5MBPbIqV92AaeXatLxBI9gBaebbnrfifHhDYfgasaacH8akY=wiFfYdH8Gipec8Eeeu0xXdbba9frFj0=OqFfea0dXdd9vqai=hGuQ8kuc9pgc9s8qqaq=dirpe0xb9q8qiLsFr0=vr0=vr0dc8meaabaqaciaacaGaaeqabaqabeGadaaakeaadaqiaaqaaiabd2eanjabdofatbGaayPadaaaaa@2FC0@ := {*j *∈ [0, *n *- *m*] | *sc *(S_
 MathType@MTEF@5@5@+=feaafiart1ev1aaatCvAUfKttLearuWrP9MDH5MBPbIqV92AaeXatLxBI9gBaebbnrfifHhDYfgasaacH8akY=wiFfYdH8Gipec8Eeeu0xXdbba9frFj0=OqFfea0dXdd9vqai=hGuQ8kuc9pgc9s8qqaq=dirpe0xb9q8qiLsFr0=vr0=vr0dc8meaabaqaciaacaGaaeqabaqabeGadaaakeaadaqiaaqaaiabdofatbGaayPadaaaaa@2E9D@[*j*..*j *+ *m *- 1], M_
 MathType@MTEF@5@5@+=feaafiart1ev1aaatCvAUfKttLearuWrP9MDH5MBPbIqV92AaeXatLxBI9gBaebbnrfifHhDYfgasaacH8akY=wiFfYdH8Gipec8Eeeu0xXdbba9frFj0=OqFfea0dXdd9vqai=hGuQ8kuc9pgc9s8qqaq=dirpe0xb9q8qiLsFr0=vr0=vr0dc8meaabaqaciaacaGaaeqabaqabeGadaaakeaadaqiaaqaaiabd2eanbGaayPadaaaaa@2E91@) ≥ *th*}.

Now observe that we can use matches of M_
 MathType@MTEF@5@5@+=feaafiart1ev1aaatCvAUfKttLearuWrP9MDH5MBPbIqV92AaeXatLxBI9gBaebbnrfifHhDYfgasaacH8akY=wiFfYdH8Gipec8Eeeu0xXdbba9frFj0=OqFfea0dXdd9vqai=hGuQ8kuc9pgc9s8qqaq=dirpe0xb9q8qiLsFr0=vr0=vr0dc8meaabaqaciaacaGaaeqabaqabeGadaaakeaadaqiaaqaaiabd2eanbGaayPadaaaaa@2E91@ on S_
 MathType@MTEF@5@5@+=feaafiart1ev1aaatCvAUfKttLearuWrP9MDH5MBPbIqV92AaeXatLxBI9gBaebbnrfifHhDYfgasaacH8akY=wiFfYdH8Gipec8Eeeu0xXdbba9frFj0=OqFfea0dXdd9vqai=hGuQ8kuc9pgc9s8qqaq=dirpe0xb9q8qiLsFr0=vr0=vr0dc8meaabaqaciaacaGaaeqabaqabeGadaaakeaadaqiaaqaaiabdofatbGaayPadaaaaa@2E9D@, for the computation of matches of *M *on *S*, since *MS *⊆ MS_
 MathType@MTEF@5@5@+=feaafiart1ev1aaatCvAUfKttLearuWrP9MDH5MBPbIqV92AaeXatLxBI9gBaebbnrfifHhDYfgasaacH8akY=wiFfYdH8Gipec8Eeeu0xXdbba9frFj0=OqFfea0dXdd9vqai=hGuQ8kuc9pgc9s8qqaq=dirpe0xb9q8qiLsFr0=vr0=vr0dc8meaabaqaciaacaGaaeqabaqabeGadaaakeaadaqiaaqaaiabd2eanjabdofatbGaayPadaaaaa@2FC0@. We prove that *MS *⊆ MS_
 MathType@MTEF@5@5@+=feaafiart1ev1aaatCvAUfKttLearuWrP9MDH5MBPbIqV92AaeXatLxBI9gBaebbnrfifHhDYfgasaacH8akY=wiFfYdH8Gipec8Eeeu0xXdbba9frFj0=OqFfea0dXdd9vqai=hGuQ8kuc9pgc9s8qqaq=dirpe0xb9q8qiLsFr0=vr0=vr0dc8meaabaqaciaacaGaaeqabaqabeGadaaakeaadaqiaaqaaiabd2eanjabdofatbGaayPadaaaaa@2FC0@ holds for all *th *∈ [*sc*_min _(*M*), *sc*_max_(*M*)] by proving the more general statement given in the following Lemma.

**Lemma 2 ***sc *(*w*, *M*) ≤ *sc *(w_
 MathType@MTEF@5@5@+=feaafiart1ev1aaatCvAUfKttLearuWrP9MDH5MBPbIqV92AaeXatLxBI9gBaebbnrfifHhDYfgasaacH8akY=wiFfYdH8Gipec8Eeeu0xXdbba9frFj0=OqFfea0dXdd9vqai=hGuQ8kuc9pgc9s8qqaq=dirpe0xb9q8qiLsFr0=vr0=vr0dc8meaabaqaciaacaGaaeqabaqabeGadaaakeaadaqiaaqaaiabdEha3bGaayPadaaaaa@2EE5@, M_
 MathType@MTEF@5@5@+=feaafiart1ev1aaatCvAUfKttLearuWrP9MDH5MBPbIqV92AaeXatLxBI9gBaebbnrfifHhDYfgasaacH8akY=wiFfYdH8Gipec8Eeeu0xXdbba9frFj0=OqFfea0dXdd9vqai=hGuQ8kuc9pgc9s8qqaq=dirpe0xb9q8qiLsFr0=vr0=vr0dc8meaabaqaciaacaGaaeqabaqabeGadaaakeaadaqiaaqaaiabd2eanbGaayPadaaaaa@2E91@) holds for all *w *∈ A
 MathType@MTEF@5@5@+=feaafiart1ev1aaatCvAUfKttLearuWrP9MDH5MBPbIqV92AaeXatLxBI9gBamrtHrhAL1wy0L2yHvtyaeHbnfgDOvwBHrxAJfwnaebbnrfifHhDYfgasaacH8akY=wiFfYdH8Gipec8Eeeu0xXdbba9frFj0=OqFfea0dXdd9vqai=hGuQ8kuc9pgc9s8qqaq=dirpe0xb9q8qiLsFr0=vr0=vr0dc8meaabaqaciaacaGaaeqabaWaaeGaeaaakeaaimaacqWFaeFqaaa@3821@^*m*^.

**Proof**:

sc(w,M)=∑i=0m−1M(i,w[i])≤∑i=0m−1max⁡{M(i,a)|a∈Φ−1(Φ(w[i]))}     =∑i=0m−1M_(i,Φ(w[i]))=sc(w_,M_).
 MathType@MTEF@5@5@+=feaafiart1ev1aaatCvAUfKttLearuWrP9MDH5MBPbIqV92AaeXatLxBI9gBaebbnrfifHhDYfgasaacH8akY=wiFfYdH8Gipec8Eeeu0xXdbba9frFj0=OqFfea0dXdd9vqai=hGuQ8kuc9pgc9s8qqaq=dirpe0xb9q8qiLsFr0=vr0=vr0dc8meaabaqaciaacaGaaeqabaqabeGadaaakqGabeWaaGJcaqodaasdbaGaem4CamNaem4yam2aaeWaceaacqWG3bWDcqGGSaalcqWGnbqtaiaawIcacaGLPaaacqGH9aqpdaaeWbqaaiabd2eannaabmGabaGaemyAaKMaeiilaWIaem4DaCNaei4waSLaemyAaKMaeiyxa0facaGLOaGaayzkaaGaeyizImkaleaacqWGPbqAcqGH9aqpcqaIWaamaeaacqWGTbqBcqGHsislcqaIXaqma0GaeyyeIuoakmaaqahabaGagiyBa0MaeiyyaeMaeiiEaG3aaiWabeaacqWGnbqtdaqadiqaaiabdMgaPjabcYcaSiabdggaHbGaayjkaiaawMcaaiabcYha8jabdggaHHGaaiab=HGiolab=z6agnaaCaaaleqabaGaeyOeI0IaeGymaedaaOWaaeWaceaacqWFMoGrdaqadiqaaiabdEha3jabcUfaBjabdMgaPjabc2faDbGaayjkaiaawMcaaaGaayjkaiaawMcaaaGaay5Eaiaaw2haaaWcbaGaemyAaKMaeyypa0JaeGimaadabaGaemyBa0MaeyOeI0IaeGymaedaniabggHiLdaakeaacaWLjaGaaCzcaiaaxMaacqGH9aqpdaaeWbqaamaaHaaabaGaemyta0eacaGLcmaaaSqaaiabdMgaPjabg2da9iabicdaWaqaaiabd2gaTjabgkHiTiabigdaXaqdcqGHris5aOWaaeWaceaacqWGPbqAcqGGSaalcqWFMoGrdaqadiqaaGqaciab+Dha3jab=TfaBjab+LgaPjab=1faDbGaayjkaiaawMcaaaGaayjkaiaawMcaaiabg2da9iabdohaZjabdogaJnaabmGabaWaaecaaeaacqWG3bWDaiaawkWaaiabcYcaSmaaHaaabaGaemyta0eacaGLcmaaaiaawIcacaGLPaaacqGGUaGlaaaa@97B7@

Thus the following implications follow directly

• *sc *(*w*, *M*) ≥ *th *⇒ *sc *(w_
 MathType@MTEF@5@5@+=feaafiart1ev1aaatCvAUfKttLearuWrP9MDH5MBPbIqV92AaeXatLxBI9gBaebbnrfifHhDYfgasaacH8akY=wiFfYdH8Gipec8Eeeu0xXdbba9frFj0=OqFfea0dXdd9vqai=hGuQ8kuc9pgc9s8qqaq=dirpe0xb9q8qiLsFr0=vr0=vr0dc8meaabaqaciaacaGaaeqabaqabeGadaaakeaadaqiaaqaaiabdEha3bGaayPadaaaaa@2EE5@, M_
 MathType@MTEF@5@5@+=feaafiart1ev1aaatCvAUfKttLearuWrP9MDH5MBPbIqV92AaeXatLxBI9gBaebbnrfifHhDYfgasaacH8akY=wiFfYdH8Gipec8Eeeu0xXdbba9frFj0=OqFfea0dXdd9vqai=hGuQ8kuc9pgc9s8qqaq=dirpe0xb9q8qiLsFr0=vr0=vr0dc8meaabaqaciaacaGaaeqabaqabeGadaaakeaadaqiaaqaaiabd2eanbGaayPadaaaaa@2E91@) ≥ *th*

• *i *∈ *MS *⇒ *i *∈ MS_
 MathType@MTEF@5@5@+=feaafiart1ev1aaatCvAUfKttLearuWrP9MDH5MBPbIqV92AaeXatLxBI9gBaebbnrfifHhDYfgasaacH8akY=wiFfYdH8Gipec8Eeeu0xXdbba9frFj0=OqFfea0dXdd9vqai=hGuQ8kuc9pgc9s8qqaq=dirpe0xb9q8qiLsFr0=vr0=vr0dc8meaabaqaciaacaGaaeqabaqabeGadaaakeaadaqiaaqaaiabd2eanjabdofatbGaayPadaaaaa@2FC0@

and we conclude: *MS *⊆ MS_
 MathType@MTEF@5@5@+=feaafiart1ev1aaatCvAUfKttLearuWrP9MDH5MBPbIqV92AaeXatLxBI9gBaebbnrfifHhDYfgasaacH8akY=wiFfYdH8Gipec8Eeeu0xXdbba9frFj0=OqFfea0dXdd9vqai=hGuQ8kuc9pgc9s8qqaq=dirpe0xb9q8qiLsFr0=vr0=vr0dc8meaabaqaciaacaGaaeqabaqabeGadaaakeaadaqiaaqaaiabd2eanjabdofatbGaayPadaaaaa@2FC0@ holds for *th *∈ [*sc*_min _(*M*), *sc*_max _(*M*)].

Hence we can search with M_
 MathType@MTEF@5@5@+=feaafiart1ev1aaatCvAUfKttLearuWrP9MDH5MBPbIqV92AaeXatLxBI9gBaebbnrfifHhDYfgasaacH8akY=wiFfYdH8Gipec8Eeeu0xXdbba9frFj0=OqFfea0dXdd9vqai=hGuQ8kuc9pgc9s8qqaq=dirpe0xb9q8qiLsFr0=vr0=vr0dc8meaabaqaciaacaGaaeqabaqabeGadaaakeaadaqiaaqaaiabd2eanbGaayPadaaaaa@2E91@ in S_
 MathType@MTEF@5@5@+=feaafiart1ev1aaatCvAUfKttLearuWrP9MDH5MBPbIqV92AaeXatLxBI9gBaebbnrfifHhDYfgasaacH8akY=wiFfYdH8Gipec8Eeeu0xXdbba9frFj0=OqFfea0dXdd9vqai=hGuQ8kuc9pgc9s8qqaq=dirpe0xb9q8qiLsFr0=vr0=vr0dc8meaabaqaciaacaGaaeqabaqabeGadaaakeaadaqiaaqaaiabdofatbGaayPadaaaaa@2E9D@ prefiltering of matches to *M *in *S*, profiting of longer and larger ℓ-intervals in S_
 MathType@MTEF@5@5@+=feaafiart1ev1aaatCvAUfKttLearuWrP9MDH5MBPbIqV92AaeXatLxBI9gBaebbnrfifHhDYfgasaacH8akY=wiFfYdH8Gipec8Eeeu0xXdbba9frFj0=OqFfea0dXdd9vqai=hGuQ8kuc9pgc9s8qqaq=dirpe0xb9q8qiLsFr0=vr0=vr0dc8meaabaqaciaacaGaaeqabaqabeGadaaakeaadaqiaaqaaiabdofatbGaayPadaaaaa@2E9D@ by extending algorithm *ESAsearch *as follows:

(1) Transform *S *into S_
 MathType@MTEF@5@5@+=feaafiart1ev1aaatCvAUfKttLearuWrP9MDH5MBPbIqV92AaeXatLxBI9gBaebbnrfifHhDYfgasaacH8akY=wiFfYdH8Gipec8Eeeu0xXdbba9frFj0=OqFfea0dXdd9vqai=hGuQ8kuc9pgc9s8qqaq=dirpe0xb9q8qiLsFr0=vr0=vr0dc8meaabaqaciaacaGaaeqabaqabeGadaaakeaadaqiaaqaaiabdofatbGaayPadaaaaa@2E9D@ and build the enhanced suffix array for S_
 MathType@MTEF@5@5@+=feaafiart1ev1aaatCvAUfKttLearuWrP9MDH5MBPbIqV92AaeXatLxBI9gBaebbnrfifHhDYfgasaacH8akY=wiFfYdH8Gipec8Eeeu0xXdbba9frFj0=OqFfea0dXdd9vqai=hGuQ8kuc9pgc9s8qqaq=dirpe0xb9q8qiLsFr0=vr0=vr0dc8meaabaqaciaacaGaaeqabaqabeGadaaakeaadaqiaaqaaiabdofatbGaayPadaaaaa@2E9D@;

(2) Construct M_
 MathType@MTEF@5@5@+=feaafiart1ev1aaatCvAUfKttLearuWrP9MDH5MBPbIqV92AaeXatLxBI9gBaebbnrfifHhDYfgasaacH8akY=wiFfYdH8Gipec8Eeeu0xXdbba9frFj0=OqFfea0dXdd9vqai=hGuQ8kuc9pgc9s8qqaq=dirpe0xb9q8qiLsFr0=vr0=vr0dc8meaabaqaciaacaGaaeqabaqabeGadaaakeaadaqiaaqaaiabd2eanbGaayPadaaaaa@2E91@ from *M*;

(3) Compute MS_
 MathType@MTEF@5@5@+=feaafiart1ev1aaatCvAUfKttLearuWrP9MDH5MBPbIqV92AaeXatLxBI9gBaebbnrfifHhDYfgasaacH8akY=wiFfYdH8Gipec8Eeeu0xXdbba9frFj0=OqFfea0dXdd9vqai=hGuQ8kuc9pgc9s8qqaq=dirpe0xb9q8qiLsFr0=vr0=vr0dc8meaabaqaciaacaGaaeqabaqabeGadaaakeaadaqiaaqaaiabd2eanjabdofatbGaayPadaaaaa@2FC0@ by searching with M_
 MathType@MTEF@5@5@+=feaafiart1ev1aaatCvAUfKttLearuWrP9MDH5MBPbIqV92AaeXatLxBI9gBaebbnrfifHhDYfgasaacH8akY=wiFfYdH8Gipec8Eeeu0xXdbba9frFj0=OqFfea0dXdd9vqai=hGuQ8kuc9pgc9s8qqaq=dirpe0xb9q8qiLsFr0=vr0=vr0dc8meaabaqaciaacaGaaeqabaqabeGadaaakeaadaqiaaqaaiabd2eanbGaayPadaaaaa@2E91@ on the enhanced suffix array of S_
 MathType@MTEF@5@5@+=feaafiart1ev1aaatCvAUfKttLearuWrP9MDH5MBPbIqV92AaeXatLxBI9gBaebbnrfifHhDYfgasaacH8akY=wiFfYdH8Gipec8Eeeu0xXdbba9frFj0=OqFfea0dXdd9vqai=hGuQ8kuc9pgc9s8qqaq=dirpe0xb9q8qiLsFr0=vr0=vr0dc8meaabaqaciaacaGaaeqabaqabeGadaaakeaadaqiaaqaaiabdofatbGaayPadaaaaa@2E9D@ using algorithm *ESAsearch*;

(4) For each *i *∈ MS_
 MathType@MTEF@5@5@+=feaafiart1ev1aaatCvAUfKttLearuWrP9MDH5MBPbIqV92AaeXatLxBI9gBaebbnrfifHhDYfgasaacH8akY=wiFfYdH8Gipec8Eeeu0xXdbba9frFj0=OqFfea0dXdd9vqai=hGuQ8kuc9pgc9s8qqaq=dirpe0xb9q8qiLsFr0=vr0=vr0dc8meaabaqaciaacaGaaeqabaqabeGadaaakeaadaqiaaqaaiabd2eanjabdofatbGaayPadaaaaa@2FC0@ re-score match with *σ *= *sc *(*S*[*i*..*i *+ *m *- 1], *M*), and report *i *and *σ *if and only if *σ *≥ *th*.

As a further consequence of Definition 2 the maximum score values in each row of *M *and M_
 MathType@MTEF@5@5@+=feaafiart1ev1aaatCvAUfKttLearuWrP9MDH5MBPbIqV92AaeXatLxBI9gBaebbnrfifHhDYfgasaacH8akY=wiFfYdH8Gipec8Eeeu0xXdbba9frFj0=OqFfea0dXdd9vqai=hGuQ8kuc9pgc9s8qqaq=dirpe0xb9q8qiLsFr0=vr0=vr0dc8meaabaqaciaacaGaaeqabaqabeGadaaakeaadaqiaaqaaiabd2eanbGaayPadaaaaa@2E91@ and thus the intermediate thresholds remain unchanged in the transformation process. Unfortunately the necessary PSSM transformation accompanying alphabet size reduction affects the expected reading depth m*(*th*) in such a way that it increases with more degraded alphabets, and therefore reduces the expected performance improvement. Due to maximization according to Equation (3) the matrix values in M_
 MathType@MTEF@5@5@+=feaafiart1ev1aaatCvAUfKttLearuWrP9MDH5MBPbIqV92AaeXatLxBI9gBaebbnrfifHhDYfgasaacH8akY=wiFfYdH8Gipec8Eeeu0xXdbba9frFj0=OqFfea0dXdd9vqai=hGuQ8kuc9pgc9s8qqaq=dirpe0xb9q8qiLsFr0=vr0=vr0dc8meaabaqaciaacaGaaeqabaqabeGadaaakeaadaqiaaqaaiabd2eanbGaayPadaaaaa@2E91@ increase and we expect a decreased probability of falling short of an intermediate threshold early. Observe that there is a trade-off between increased expected reading depth and increased lcp-interval sizes at low reading depths. Therefore it is desirable to minimize the effect of maximization by grouping PSSM columns with similar score values, i.e., highly correlated columns. Since PSSMs reflect the properties of the underlying multiple alignment, we expect correlations of PSSM columns according to biologically motivated symbol similarities. Hence character correlation is the motivation for our alphabet reduction strategy.

#### Reduced amino acid alphabets

**Figure 8:**
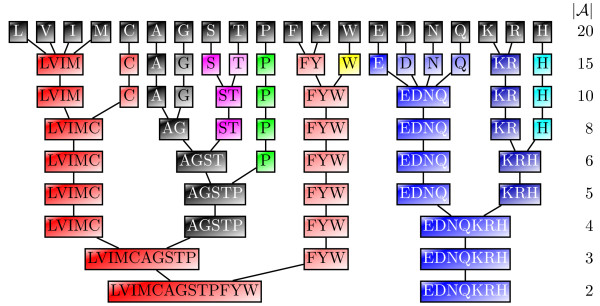
**Schemes for amino acid alphabet reduction**. Reduction of the amino acid alphabet into smaller groups. Amino acid pairs are iteratively grouped together based on ther correlations *c*_*a*,*b *_(see text for the definition of *c*_*a*,*b*_), starting with the most correlated pairs, until al amino acids are divided into the desired number of groups. Here we used BLOSUM50 similarities for the determination of *c*_*a*,*b*_. Observe that, hydrophobic amino acids, especially (LVIM) and (FYW) are conserved in many reduced alphabets. The same is true for the polar (ST), (EDNQ), and (KR) groups. The smallest alphabet contains two groups that can be categorized broadly as hydrophobic/small (LVIMCAGSTPFYW) and hydrophilic (EDNQKRH).

It is well known that various of the naturally occurring amino acids share certain similarities, like similar physiochemical properties. Accordingly, the complexity of protein sequences can be reduced by sorting these amino acids with similarities into groups and deriving a transformed, reduced alphabet [29]. These reduced alphabets contain symbols that represent a specific character class of the original alphabet. Since PSSMs and the sequences to be searched have to be encoded over the same alphabet, we are more interested in a single reduced alphabet suitable for all PSSMs under consideration, than in PSSM-specific reduced alphabets. The latter implies an unacceptable overhead of index generation for sequences over PSSM-specific alphabets, even though it may result in a lower expected reading depth. The basis for our reduction of the 20-letter amino acid alphabet to smaller alphabets are correlations indicated by the BLOSUM similarity matrix as described in [30]. That is, amino acid pairs with high similarity scores are grouped together (see Figure 8 for an example). Let *a *and *b *be two amino acids and *Y *a 20 × 20 score matrix, then a measure of amino acid correlation *c*_*a*,*b *_between *a *and *b *can be defined as

ca,b:=∑i=120Ya,iYb,i(∑i=120Ya,i2)(∑i=120Yb,i2)
 MathType@MTEF@5@5@+=feaafiart1ev1aaatCvAUfKttLearuWrP9MDH5MBPbIqV92AaeXatLxBI9gBaebbnrfifHhDYfgasaacH8akY=wiFfYdH8Gipec8Eeeu0xXdbba9frFj0=OqFfea0dXdd9vqai=hGuQ8kuc9pgc9s8qqaq=dirpe0xb9q8qiLsFr0=vr0=vr0dc8meaabaqaciaacaGaaeqabaqabeGadaaakeaacqWGJbWydaWgaaWcbaGaemyyaeMaeiilaWIaemOyaigabeaakiabcQda6iabg2da9maalaaabaWaaabmaeaacqWGzbqwdaWgaaWcbaGaemyyaeMaeiilaWIaemyAaKgabeaakiabdMfaznaaBaaaleaacqWGIbGycqGGSaalcqWGPbqAaeqaaaqaaiabdMgaPjabg2da9iabigdaXaqaaiabikdaYiabicdaWaqdcqGHris5aaGcbaWaaeWaceaadaaeWaqaaiabdMfaznaaDaaaleaacqWGHbqycqGGSaalcqWGPbqAaeaacqaIYaGmaaaabaGaemyAaKMaeyypa0JaeGymaedabaGaeGOmaiJaeGimaadaniabggHiLdaakiaawIcacaGLPaaadaqadiqaamaaqadabaGaemywaK1aa0baaSqaaiabdkgaIjabcYcaSiabdMgaPbqaaiabikdaYaaaaeaacqWGPbqAcqGH9aqpcqaIXaqmaeaacqaIYaGmcqaIWaama0GaeyyeIuoaaOGaayjkaiaawMcaaaaaaaa@620F@

and amino acid pairs can be iteratively grouped together according to their correlations, starting with the most correlated pairs, until all the amino acids are divided into the desired number of groups.

### Finding an appropriate threshold for PSSM searching: LazyDistrib

#### Probabilities and expectation values

The results of PSSM searches strongly depend on the choice of an appropriate threshold value *th*. A small threshold may produce a large number of false positive matches without any biological meaning, whereas meaningful matches may not be found if the threshold is too stringent. PSSM-scores are not equally distributed and thus scores of two different PSSMs are not comparable. It is therefore desirable to let the user define a significance threshold instead. The expected number of matches in a given random sequence database (E-value) is a widely accepted measure of the significance. We can compute the E-value for a known background distribution and length of the database by exhaustive enumeration of all substrings. However, the time complexity of such a computation is O
 MathType@MTEF@5@5@+=feaafiart1ev1aaatCvAUfKttLearuWrP9MDH5MBPbIqV92AaeXatLxBI9gBamrtHrhAL1wy0L2yHvtyaeHbnfgDOvwBHrxAJfwnaebbnrfifHhDYfgasaacH8akY=wiFfYdH8Gipec8Eeeu0xXdbba9frFj0=OqFfea0dXdd9vqai=hGuQ8kuc9pgc9s8qqaq=dirpe0xb9q8qiLsFr0=vr0=vr0dc8meaabaqaciaacaGaaeqabaWaaeGaeaaakeaaimaacqWFoe=taaa@383D@(|A
 MathType@MTEF@5@5@+=feaafiart1ev1aaatCvAUfKttLearuWrP9MDH5MBPbIqV92AaeXatLxBI9gBamrtHrhAL1wy0L2yHvtyaeHbnfgDOvwBHrxAJfwnaebbnrfifHhDYfgasaacH8akY=wiFfYdH8Gipec8Eeeu0xXdbba9frFj0=OqFfea0dXdd9vqai=hGuQ8kuc9pgc9s8qqaq=dirpe0xb9q8qiLsFr0=vr0=vr0dc8meaabaqaciaacaGaaeqabaWaaeGaeaaakeaaimaacqWFaeFqaaa@3821@|^*m*^*m*) for a PSSM of length *m*. If the values in *M *are integers within a certain range [*r*_*min*_, *r*_*max*_] of size *R *= *r*_*max *_- *r*_*min *_+ 1, then dynamic programming (DP) methods (cf. [12,21,22]) allow to compute the probability distribution (and hence the E-value) in O
 MathType@MTEF@5@5@+=feaafiart1ev1aaatCvAUfKttLearuWrP9MDH5MBPbIqV92AaeXatLxBI9gBamrtHrhAL1wy0L2yHvtyaeHbnfgDOvwBHrxAJfwnaebbnrfifHhDYfgasaacH8akY=wiFfYdH8Gipec8Eeeu0xXdbba9frFj0=OqFfea0dXdd9vqai=hGuQ8kuc9pgc9s8qqaq=dirpe0xb9q8qiLsFr0=vr0=vr0dc8meaabaqaciaacaGaaeqabaWaaeGaeaaakeaaimaacqWFoe=taaa@383D@(*m*^2^*R*|A
 MathType@MTEF@5@5@+=feaafiart1ev1aaatCvAUfKttLearuWrP9MDH5MBPbIqV92AaeXatLxBI9gBamrtHrhAL1wy0L2yHvtyaeHbnfgDOvwBHrxAJfwnaebbnrfifHhDYfgasaacH8akY=wiFfYdH8Gipec8Eeeu0xXdbba9frFj0=OqFfea0dXdd9vqai=hGuQ8kuc9pgc9s8qqaq=dirpe0xb9q8qiLsFr0=vr0=vr0dc8meaabaqaciaacaGaaeqabaWaaeGaeaaakeaaimaacqWFaeFqaaa@3821@|) time.

In practice the probability distribution is often not exactly, or completely calculated due to concerns of speed. E.g., in the *EMATRIX *system [12] score thresholds are calculated and stored for probability values in the interval *π *= 10^-1^, 10^-2^,..., 10^-40 ^only. Consequently, the user can only specify one of these p-value cutoffs. For the calculation of the p-value from a determined match score, *EMATRIX *uses log-linear interpolation on the stored thresholds. A different, commonly used strategy to derive a continuous distribution function uses the extreme value distribution as an approximation [31-33] of high scoring matches.

Even though it is widely accepted that high-scoring local alignment score distributions of the popular position independent scoring systems PAM and BLOSUM can be well approximated by an extreme value distribution, this cannot be generalized for arbitrary PSSMs.

**Figure 9:**
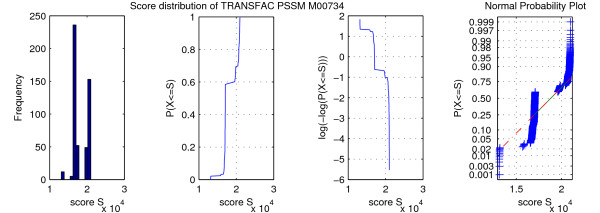
**Score distribution of TRANSFAC PSSM M00734**. Histogram, cumulative score distribution function, X-Y plot, and normal probability plot of TRANSFAC PSSM M00734 (PSSM length *m *= 9).

**Figure 10:**
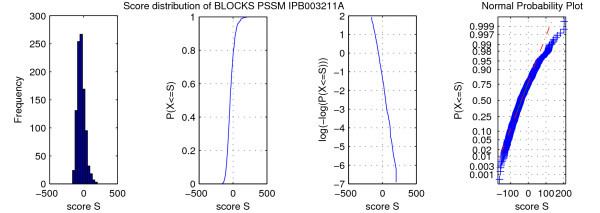
**Score distribution of BLOCKS PSSM IPB003211A**. Histogram, cumulative score distribution, X-Y plot, and normal probability plot of a PSSM taken from the BLOCKS database (Accession: IPB003211A; PSSM length *m *= 40), describing the UreI protein of *Helicobacter pylori*, a proton gated urea channel [36].

To check whether an extreme value distribution is a suitable approximation for the distribution of PSSM match scores, we sampled the match scores of PSSMs arbitrarily chosen from the TRANSFAC and BLOCKS database. We randomly shuffled 1000 human promotor sequences of length 1200, taken from the database of transcriptional start sites (DBTSS) and 1000 protein sequences of length 365 (= average sequence length in Uniprot-Swissprot), respectively, preserving their mono-symbol composition. From the derived random PSSM match scores we took the best score for each sequence and calculated the empirical cumulative distribution function. If the match scores *S *are extreme value distributed, a X-Y plot with *X *= *S *and *Y *= log(- log(*S*)) should appear linear, since log(−log⁡(e−e−λ(x−u)))=−λ(x−u)
 MathType@MTEF@5@5@+=feaafiart1ev1aaatCvAUfKttLearuWrP9MDH5MBPbIqV92AaeXatLxBI9gBaebbnrfifHhDYfgasaacH8akY=wiFfYdH8Gipec8Eeeu0xXdbba9frFj0=OqFfea0dXdd9vqai=hGuQ8kuc9pgc9s8qqaq=dirpe0xb9q8qiLsFr0=vr0=vr0dc8meaabaqaciaacaGaaeqabaqabeGadaaakeaadaqadiqaaiabgkHiTiGbcYgaSjabc+gaVjabcEgaNnaabmGabaGaemyzau2aaWbaaSqabeaacqGHsislcqWGLbqzdaahaaadbeqaaGGaciab=jHiTiab=T7aSnaabmGabaGaemiEaGNaeyOeI0IaemyDauhacaGLOaGaayzkaaaaaaaaaOGaayjkaiaawMcaaaGaayjkaiaawMcaaiabg2da9iabgkHiTiab=T7aSnaabmGabaGaemiEaGNaeyOeI0IaemyDauhacaGLOaGaayzkaaaaaa@49D0@ holds. For the TRANSFAC PSSM shown in Figure 9, the X-Y plot clearly indicates that an extreme value distribution is not an appropriate approximation. For PSSM IPB003211A (see Figure 10) from the BLOCKS database, it seems as if the score distribution can be approximated quite well with an extreme value distribution. However, we then still have the problem of adequate parameter estimation for the distribution function. Since we do not make any assumptions about the used PSSMs in our algorithm, neither about the type of scores, nor the score range, a proper approximation of the score distribution of arbitrary PSSMs is not possible, without time consuming simulations. That is why we are more interested in an exact solution and thus we focus on the efficient computation of an exact discrete score distribution.

#### Calculation of exact PSSM score distributions

While recent publications focus on the computation of the complete probability distribution, what is required specifically for PSSM matching, is computing a partial cumulative distribution corresponding to an E-value resp. p-value specified by the user. Therefore, we have developed a new "lazy" method to efficiently compute only a small fraction of the complete distribution.

We formulate the problem we solve w.r.t. E-values and p-values: Given a user specified E-value *η*, find the minimum threshold *Tmin*_*E*_(η, *M*), such that the expected number of matches of *M *in a random sequence of given length is at most *η*. Given a user specified p-value *π*, find the minimum threshold TminP
 MathType@MTEF@5@5@+=feaafiart1ev1aaatCvAUfKttLearuWrP9MDH5MBPbIqV92AaeXatLxBI9gBamrtHrhAL1wy0L2yHvtyaeHbnfgDOvwBHrxAJfwnaebbnrfifHhDYfgasaacH8akY=wiFfYdH8Gipec8Eeeu0xXdbba9frFj0=OqFfea0dXdd9vqai=hGuQ8kuc9pgc9s8qqaq=dirpe0xb9q8qiLsFr0=vr0=vr0dc8meaabaqaciaacaGaaeqabaWaaeGaeaaakeaacqWGubavieGacqWFTbqBcqWFPbqAcqWFUbGBdaWgaaWcbaGaemiuaafabeaaaaa@3D07@(*π*, *M*), such that the probability that *M *matches a random string of length *m *is at most *π*. The threshold *Tmin*_*E *_(*η*, *M*) can be computed from TminP
 MathType@MTEF@5@5@+=feaafiart1ev1aaatCvAUfKttLearuWrP9MDH5MBPbIqV92AaeXatLxBI9gBamrtHrhAL1wy0L2yHvtyaeHbnfgDOvwBHrxAJfwnaebbnrfifHhDYfgasaacH8akY=wiFfYdH8Gipec8Eeeu0xXdbba9frFj0=OqFfea0dXdd9vqai=hGuQ8kuc9pgc9s8qqaq=dirpe0xb9q8qiLsFr0=vr0=vr0dc8meaabaqaciaacaGaaeqabaWaaeGaeaaakeaacqWGubavieGacqWFTbqBcqWFPbqAcqWFUbGBdaWgaaWcbaGaemiuaafabeaaaaa@3D07@(*π*, *M*) according to the equation *Tmin*_*E*_(*π*·(*n *- *m *+ 1), *M*) = TminP
 MathType@MTEF@5@5@+=feaafiart1ev1aaatCvAUfKttLearuWrP9MDH5MBPbIqV92AaeXatLxBI9gBamrtHrhAL1wy0L2yHvtyaeHbnfgDOvwBHrxAJfwnaebbnrfifHhDYfgasaacH8akY=wiFfYdH8Gipec8Eeeu0xXdbba9frFj0=OqFfea0dXdd9vqai=hGuQ8kuc9pgc9s8qqaq=dirpe0xb9q8qiLsFr0=vr0=vr0dc8meaabaqaciaacaGaaeqabaWaaeGaeaaakeaacqWGubavieGacqWFTbqBcqWFPbqAcqWFUbGBdaWgaaWcbaGaemiuaafabeaaaaa@3D07@(*π*, *M*). Hence we restrict on computing TminP
 MathType@MTEF@5@5@+=feaafiart1ev1aaatCvAUfKttLearuWrP9MDH5MBPbIqV92AaeXatLxBI9gBamrtHrhAL1wy0L2yHvtyaeHbnfgDOvwBHrxAJfwnaebbnrfifHhDYfgasaacH8akY=wiFfYdH8Gipec8Eeeu0xXdbba9frFj0=OqFfea0dXdd9vqai=hGuQ8kuc9pgc9s8qqaq=dirpe0xb9q8qiLsFr0=vr0=vr0dc8meaabaqaciaacaGaaeqabaWaaeGaeaaakeaacqWGubavieGacqWFTbqBcqWFPbqAcqWFUbGBdaWgaaWcbaGaemiuaafabeaaaaa@3D07@(*π*, *M*).

Since all strings of length *m *have a score between *sc*_min _(*M*) and *sc*_max_(*M*), we conclude TminP
 MathType@MTEF@5@5@+=feaafiart1ev1aaatCvAUfKttLearuWrP9MDH5MBPbIqV92AaeXatLxBI9gBamrtHrhAL1wy0L2yHvtyaeHbnfgDOvwBHrxAJfwnaebbnrfifHhDYfgasaacH8akY=wiFfYdH8Gipec8Eeeu0xXdbba9frFj0=OqFfea0dXdd9vqai=hGuQ8kuc9pgc9s8qqaq=dirpe0xb9q8qiLsFr0=vr0=vr0dc8meaabaqaciaacaGaaeqabaWaaeGaeaaakeaacqWGubavieGacqWFTbqBcqWFPbqAcqWFUbGBdaWgaaWcbaGaemiuaafabeaaaaa@3D07@(1, *M*) = *sc*_min _(*M*) and TminP
 MathType@MTEF@5@5@+=feaafiart1ev1aaatCvAUfKttLearuWrP9MDH5MBPbIqV92AaeXatLxBI9gBamrtHrhAL1wy0L2yHvtyaeHbnfgDOvwBHrxAJfwnaebbnrfifHhDYfgasaacH8akY=wiFfYdH8Gipec8Eeeu0xXdbba9frFj0=OqFfea0dXdd9vqai=hGuQ8kuc9pgc9s8qqaq=dirpe0xb9q8qiLsFr0=vr0=vr0dc8meaabaqaciaacaGaaeqabaWaaeGaeaaakeaacqWGubavieGacqWFTbqBcqWFPbqAcqWFUbGBdaWgaaWcbaGaemiuaafabeaaaaa@3D07@(0, *M*) > *sc*_max_(*M*). To explain our lazy evaluation method, we first consider existing methods based on DP.

#### Evaluation with dynamic programming

We assume that at each position in sequence *S*, the symbols occur independently, with probability *f *(*a*) = (1/*n*)·|{*i *∈ [0, *n *- 1] |*S*[*i*] = *a*}|. Thus a substring *w *of length *m *in *S *occurs with probability ∏i=0m−1f(w[i])
MathType@MTEF@5@5@+=feaafiart1ev1aaatCvAUfKttLearuWrP9MDH5MBPbIqV92AaeXatLxBI9gBaebbnrfifHhDYfgasaacH8akY=wiFfYdH8Gipec8Eeeu0xXdbba9frFj0=OqFfea0dXdd9vqai=hGuQ8kuc9pgc9s8qqaq=dirpe0xb9q8qiLsFr0=vr0=vr0dc8meaabaqaciaacaGaaeqabaqabeGadaaakeaadaqeWaqaaiabdAgaMnaabmGabaGaem4DaCNaei4waSLaemyAaKMaeiyxa0facaGLOaGaayzkaaaaleaacqWGPbqAcqGH9aqpcqaIWaamaeaacqWGTbqBcqGHsislcqaIXaqma0Gaey4dIunaaaa@3D5E@ and the probability of observing the event *sc *(*w*, *M*) = *t *is ℙ[sc(w,M)=t]=∑w∈Am:sc(w,M)=t∏i=0m−1f(w[i])
MathType@MTEF@5@5@+=feaafiart1ev1aaatCvAUfKttLearuWrP9MDH5MBPbIqV92AaeXatLxBI9gBamrtHrhAL1wy0L2yHvtyaeHbnfgDOvwBHrxAJfwnaebbnrfifHhDYfgasaacH8akY=wiFfYdH8Gipec8Eeeu0xXdbba9frFj0=OqFfea0dXdd9vqai=hGuQ8kuc9pgc9s8qqaq=dirpe0xb9q8qiLsFr0=vr0=vr0dc8meaabaqaciaacaGaaeqabaWaaeGaeaaakeaatuuDJXwAK1uy0HMmaeXbfv3ySLgzG0uy0HgiuD3BaGqbaiab=LriqnaadmaabaGaem4CamNaem4yamMaeiikaGIaem4DaCNaeiilaWIaemyta0KaeiykaKIaeyypa0JaemiDaqhacaGLBbGaayzxaaGaeyypa0ZaaabeaeaadaqeWaqaaiabdAgaMjabcIcaOiabdEha3jabcUfaBjabdMgaPjabc2faDjabcMcaPaWcbaGaemyAaKMaeyypa0JaeGimaadabaGaemyBa0MaeyOeI0IaeGymaedaniabg+GivdaaleaacqWG3bWDcqGHiiIZimaacqGFaeFqdaahaaadbeqaaiabd2gaTbaaliabcQda6iabdohaZjabdogaJjabcIcaOiabdEha3jabcYcaSiabd2eanjabcMcaPiabg2da9iabdsha0bqab0GaeyyeIuoaaaa@72DC@. We obtain TminP
 MathType@MTEF@5@5@+=feaafiart1ev1aaatCvAUfKttLearuWrP9MDH5MBPbIqV92AaeXatLxBI9gBamrtHrhAL1wy0L2yHvtyaeHbnfgDOvwBHrxAJfwnaebbnrfifHhDYfgasaacH8akY=wiFfYdH8Gipec8Eeeu0xXdbba9frFj0=OqFfea0dXdd9vqai=hGuQ8kuc9pgc9s8qqaq=dirpe0xb9q8qiLsFr0=vr0=vr0dc8meaabaqaciaacaGaaeqabaWaaeGaeaaakeaacqWGubavieGacqWFTbqBcqWFPbqAcqWFUbGBdaWgaaWcbaGaemiuaafabeaaaaa@3D07@(*π*, *M*) by a look-up in the distribution:

TminP
 MathType@MTEF@5@5@+=feaafiart1ev1aaatCvAUfKttLearuWrP9MDH5MBPbIqV92AaeXatLxBI9gBamrtHrhAL1wy0L2yHvtyaeHbnfgDOvwBHrxAJfwnaebbnrfifHhDYfgasaacH8akY=wiFfYdH8Gipec8Eeeu0xXdbba9frFj0=OqFfea0dXdd9vqai=hGuQ8kuc9pgc9s8qqaq=dirpe0xb9q8qiLsFr0=vr0=vr0dc8meaabaqaciaacaGaaeqabaWaaeGaeaaakeaacqWGubavieGacqWFTbqBcqWFPbqAcqWFUbGBdaWgaaWcbaGaemiuaafabeaaaaa@3D07@(*π*, *M*) = min{*t *|*sc*_min _(*M*) ≤ *t *≤ *sc*_max _(*M*), ℙ [*sc *(*w*, *M*) ≥ *t*] ≤ *π*}.

If the values in the PSSM *M *are integers in a range of width *R*, dynamic programming allows to efficiently compute the probability distribution. The dynamic programming aspect becomes more obvious by introducing for each *k *∈ [0, *m *- 1] the *prefix *PSSM *M*_*k *_: [0, *k*] × A
 MathType@MTEF@5@5@+=feaafiart1ev1aaatCvAUfKttLearuWrP9MDH5MBPbIqV92AaeXatLxBI9gBamrtHrhAL1wy0L2yHvtyaeHbnfgDOvwBHrxAJfwnaebbnrfifHhDYfgasaacH8akY=wiFfYdH8Gipec8Eeeu0xXdbba9frFj0=OqFfea0dXdd9vqai=hGuQ8kuc9pgc9s8qqaq=dirpe0xb9q8qiLsFr0=vr0=vr0dc8meaabaqaciaacaGaaeqabaWaaeGaeaaakeaaimaacqWFaeFqaaa@3821@ → ℕ defined by *M*_*k *_(*j*, *a*) = *M *(*j*, *a*) for *j *∈ [0, *k*] and *a *∈ A
 MathType@MTEF@5@5@+=feaafiart1ev1aaatCvAUfKttLearuWrP9MDH5MBPbIqV92AaeXatLxBI9gBamrtHrhAL1wy0L2yHvtyaeHbnfgDOvwBHrxAJfwnaebbnrfifHhDYfgasaacH8akY=wiFfYdH8Gipec8Eeeu0xXdbba9frFj0=OqFfea0dXdd9vqai=hGuQ8kuc9pgc9s8qqaq=dirpe0xb9q8qiLsFr0=vr0=vr0dc8meaabaqaciaacaGaaeqabaWaaeGaeaaakeaaimaacqWFaeFqaaa@3821@.

Corresponding distributions *Q*_*k *_(*t*) for *k *∈ [0, *m *- 1] and *t *∈ [*sc*_min _(*M*_*k*_), *sc*_max _(*M*_*k*_)], and *Q*_-1_(*t*), are defined by

Q−1(t):={1if t=00otherwiseQk(t):=∑a∈AQk−1(t−M(k,a))f(a)
 MathType@MTEF@5@5@+=feaafiart1ev1aaatCvAUfKttLearuWrP9MDH5MBPbIqV92AaeXatLxBI9gBamrtHrhAL1wy0L2yHvtyaeHbnfgDOvwBHrxAJfwnaebbnrfifHhDYfgasaacH8akY=wiFfYdH8Gipec8Eeeu0xXdbba9frFj0=OqFfea0dXdd9vqai=hGuQ8kuc9pgc9s8qqaq=dirpe0xb9q8qiLsFr0=vr0=vr0dc8meaabaqaciaacaGaaeqabaWaaeGaeaaakeaafaqadeGabaaabaGaemyuae1aaSbaaSqaaiabgkHiTiabigdaXaqabaGccqGGOaakcqWG0baDcqGGPaqkcqGG6aGocqGH9aqpdaGabaqaauaabaqaciaaaeaacqaIXaqmaeaacqqGPbqAcqqGMbGzcqqGGaaicqWG0baDcqGH9aqpcqaIWaamaeaacqaIWaamaeaacqqGVbWBcqqG0baDcqqGObaAcqqGLbqzcqqGYbGCcqqG3bWDcqqGPbqAcqqGZbWCcqqGLbqzaaaacaGL7baaaeaacqWGrbqudaWgaaWcbaGaem4AaSgabeaakiabcIcaOiabdsha0jabcMcaPiabcQda6iabg2da9maaqafabaGaemyuae1aaSbaaSqaaiabdUgaRjabgkHiTiabigdaXaqabaaabaGaemyyaeMaeyicI4mcdaGae8haXheabeqdcqGHris5aOGaeiikaGIaemiDaqNaeyOeI0Iaemyta0KaeiikaGIaem4AaSMaeiilaWIaemyyaeMaeiykaKIaeiykaKIaemOzayMaeiikaGIaemyyaeMaeiykaKcaaaaa@770F@

**Figure 11:**
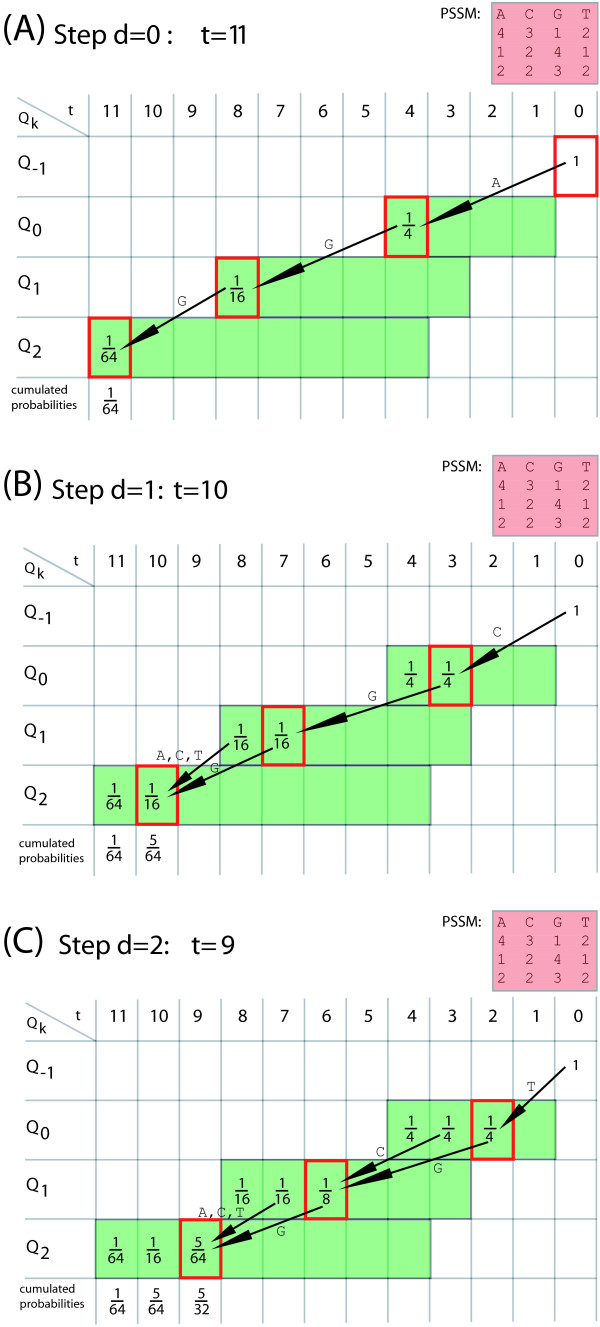
**Evaluation with dynamic programming**. The simple DP scheme computes all probability vectors *Q*_0_, *Q*_1_, *Q*_2 _completely within the green marked area, corresponding to score ranges of prefix PSSMs *M*_*k*_. In contrast to the simple scheme, the restricted probability computation method computes only the upper end of the probability distribution until the given p-value threshold is exceeded, omitting parts of the green area. In this example we show how to compute the score threshold TminP
 MathType@MTEF@5@5@+=feaafiart1ev1aaatCvAUfKttLearuWrP9MDH5MBPbIqV92AaeXatLxBI9gBamrtHrhAL1wy0L2yHvtyaeHbnfgDOvwBHrxAJfwnaebbnrfifHhDYfgasaacH8akY=wiFfYdH8Gipec8Eeeu0xXdbba9frFj0=OqFfea0dXdd9vqai=hGuQ8kuc9pgc9s8qqaq=dirpe0xb9q8qiLsFr0=vr0=vr0dc8meaabaqaciaacaGaaeqabaWaaeGaeaaakeaacqWGubavieGacqWFTbqBcqWFPbqAcqWFUbGBdaWgaaWcbaGaemiuaafabeaaaaa@3D07@(*π*, *M*) for PSSM *M *of length *m *= 3 and a score range of [4,11] corresponding to a given p-value threshold of *π *= 18
 MathType@MTEF@5@5@+=feaafiart1ev1aaatCvAUfKttLearuWrP9MDH5MBPbIqV92AaeXatLxBI9gBaebbnrfifHhDYfgasaacH8akY=wiFfYdH8Gipec8Eeeu0xXdbba9frFj0=OqFfea0dXdd9vqai=hGuQ8kuc9pgc9s8qqaq=dirpe0xb9q8qiLsFr0=vr0=vr0dc8meaabaqaciaacaGaaeqabaqabeGadaaakeaadaWcaaqaaiabigdaXaqaaiabiIda4aaaaaa@2EAA@. For simplicity we assume a uniform character distribution of *f*(*A*) = *f*(*C*) = *f*(*G*) = *f*(*T*) = 14
 MathType@MTEF@5@5@+=feaafiart1ev1aaatCvAUfKttLearuWrP9MDH5MBPbIqV92AaeXatLxBI9gBaebbnrfifHhDYfgasaacH8akY=wiFfYdH8Gipec8Eeeu0xXdbba9frFj0=OqFfea0dXdd9vqai=hGuQ8kuc9pgc9s8qqaq=dirpe0xb9q8qiLsFr0=vr0=vr0dc8meaabaqaciaacaGaaeqabaqabeGadaaakeaadaWcaaqaaiabigdaXaqaaiabisda0aaaaaa@2EA2@. Cells of the matrix that are computed in the step actually under consideration are marked red. In step *d *= 0, see (A), the algorithm computes *Q*_2_(11) recursively for all paths through *M *that achieve a score of 11, i.e. *Q*_2_(11) = *Q*_1_(8)·*f*(*G*), *Q*_1_(8) = *Q*_0_(4)·*f*(*G*), *Q*_0_(4) = *Q*_-1_(0)·*f*(*A*) = 1·14
 MathType@MTEF@5@5@+=feaafiart1ev1aaatCvAUfKttLearuWrP9MDH5MBPbIqV92AaeXatLxBI9gBaebbnrfifHhDYfgasaacH8akY=wiFfYdH8Gipec8Eeeu0xXdbba9frFj0=OqFfea0dXdd9vqai=hGuQ8kuc9pgc9s8qqaq=dirpe0xb9q8qiLsFr0=vr0=vr0dc8meaabaqaciaacaGaaeqabaqabeGadaaakeaadaWcaaqaaiabigdaXaqaaiabisda0aaaaaa@2EA2@, since AGG is the only path achieving score 11. It follows *Q*_2_(11) = 164
 MathType@MTEF@5@5@+=feaafiart1ev1aaatCvAUfKttLearuWrP9MDH5MBPbIqV92AaeXatLxBI9gBaebbnrfifHhDYfgasaacH8akY=wiFfYdH8Gipec8Eeeu0xXdbba9frFj0=OqFfea0dXdd9vqai=hGuQ8kuc9pgc9s8qqaq=dirpe0xb9q8qiLsFr0=vr0=vr0dc8meaabaqaciaacaGaaeqabaqabeGadaaakeaadaWcaaqaaiabigdaXaqaaiabiAda2iabisda0aaaaaa@2F9C@. In step *d *= 1 all paths achieving a score of 11 - *d *= 10 to determine *Q*_2_(10) are computed, see (B). We conclude *Q*_2_(10) = 116
 MathType@MTEF@5@5@+=feaafiart1ev1aaatCvAUfKttLearuWrP9MDH5MBPbIqV92AaeXatLxBI9gBaebbnrfifHhDYfgasaacH8akY=wiFfYdH8Gipec8Eeeu0xXdbba9frFj0=OqFfea0dXdd9vqai=hGuQ8kuc9pgc9s8qqaq=dirpe0xb9q8qiLsFr0=vr0=vr0dc8meaabaqaciaacaGaaeqabaqabeGadaaakeaadaWcaaqaaiabigdaXaqaaiabigdaXiabiAda2aaaaaa@2F96@. In this step, DP allows to reuse value *Q*_1_(8) without recomputation. In step *d *= 2, see (C) values *Q*_1_(7) and *Q*_0_(3) can be reused to compute *Q*_2_(9) = 564
 MathType@MTEF@5@5@+=feaafiart1ev1aaatCvAUfKttLearuWrP9MDH5MBPbIqV92AaeXatLxBI9gBaebbnrfifHhDYfgasaacH8akY=wiFfYdH8Gipec8Eeeu0xXdbba9frFj0=OqFfea0dXdd9vqai=hGuQ8kuc9pgc9s8qqaq=dirpe0xb9q8qiLsFr0=vr0=vr0dc8meaabaqaciaacaGaaeqabaqabeGadaaakeaadaWcaaqaaiabiwda1aqaaiabiAda2iabisda0aaaaaa@2FA4@. In step *d *= 2 the cumulated probability *Q*_2_(11) + *Q*_2_(10) + *Q*_2_(9) = 532
 MathType@MTEF@5@5@+=feaafiart1ev1aaatCvAUfKttLearuWrP9MDH5MBPbIqV92AaeXatLxBI9gBaebbnrfifHhDYfgasaacH8akY=wiFfYdH8Gipec8Eeeu0xXdbba9frFj0=OqFfea0dXdd9vqai=hGuQ8kuc9pgc9s8qqaq=dirpe0xb9q8qiLsFr0=vr0=vr0dc8meaabaqaciaacaGaaeqabaqabeGadaaakeaadaWcaaqaaiabiwda1aqaaiabiodaZiabikdaYaaaaaa@2F9A@ exceeds the given p-value threshold of *π *= 18
 MathType@MTEF@5@5@+=feaafiart1ev1aaatCvAUfKttLearuWrP9MDH5MBPbIqV92AaeXatLxBI9gBaebbnrfifHhDYfgasaacH8akY=wiFfYdH8Gipec8Eeeu0xXdbba9frFj0=OqFfea0dXdd9vqai=hGuQ8kuc9pgc9s8qqaq=dirpe0xb9q8qiLsFr0=vr0=vr0dc8meaabaqaciaacaGaaeqabaqabeGadaaakeaadaWcaaqaaiabigdaXaqaaiabiIda4aaaaaa@2EAA@, and the restricted probability computation method skips the rest of the computation. We obtain a score threshold of *th *= 10 correponding to *π*.

We have ℙ[*sc *(*w*, *M*) = *t*] = *Q*_*m*-1 _(*t*). The algorithm computing *Q*_*k *_determines a set of probability distributions for *M*_0_, ..., *M*_*k*_. *Q*_*k *_is evaluated in O
 MathType@MTEF@5@5@+=feaafiart1ev1aaatCvAUfKttLearuWrP9MDH5MBPbIqV92AaeXatLxBI9gBamrtHrhAL1wy0L2yHvtyaeHbnfgDOvwBHrxAJfwnaebbnrfifHhDYfgasaacH8akY=wiFfYdH8Gipec8Eeeu0xXdbba9frFj0=OqFfea0dXdd9vqai=hGuQ8kuc9pgc9s8qqaq=dirpe0xb9q8qiLsFr0=vr0=vr0dc8meaabaqaciaacaGaaeqabaWaaeGaeaaakeaaimaacqWFoe=taaa@383D@(*sc*_max _(*M*)|A
 MathType@MTEF@5@5@+=feaafiart1ev1aaatCvAUfKttLearuWrP9MDH5MBPbIqV92AaeXatLxBI9gBamrtHrhAL1wy0L2yHvtyaeHbnfgDOvwBHrxAJfwnaebbnrfifHhDYfgasaacH8akY=wiFfYdH8Gipec8Eeeu0xXdbba9frFj0=OqFfea0dXdd9vqai=hGuQ8kuc9pgc9s8qqaq=dirpe0xb9q8qiLsFr0=vr0=vr0dc8meaabaqaciaacaGaaeqabaWaaeGaeaaakeaaimaacqWFaeFqaaa@3821@|) time from *Q*_*k*-1_, summing up to O
 MathType@MTEF@5@5@+=feaafiart1ev1aaatCvAUfKttLearuWrP9MDH5MBPbIqV92AaeXatLxBI9gBamrtHrhAL1wy0L2yHvtyaeHbnfgDOvwBHrxAJfwnaebbnrfifHhDYfgasaacH8akY=wiFfYdH8Gipec8Eeeu0xXdbba9frFj0=OqFfea0dXdd9vqai=hGuQ8kuc9pgc9s8qqaq=dirpe0xb9q8qiLsFr0=vr0=vr0dc8meaabaqaciaacaGaaeqabaWaaeGaeaaakeaaimaacqWFoe=taaa@383D@(*sc*_max _(*M*) | A
 MathType@MTEF@5@5@+=feaafiart1ev1aaatCvAUfKttLearuWrP9MDH5MBPbIqV92AaeXatLxBI9gBamrtHrhAL1wy0L2yHvtyaeHbnfgDOvwBHrxAJfwnaebbnrfifHhDYfgasaacH8akY=wiFfYdH8Gipec8Eeeu0xXdbba9frFj0=OqFfea0dXdd9vqai=hGuQ8kuc9pgc9s8qqaq=dirpe0xb9q8qiLsFr0=vr0=vr0dc8meaabaqaciaacaGaaeqabaWaaeGaeaaakeaaimaacqWFaeFqaaa@3821@| *m*) total time. See Figure 11 for an example.

If we allow for floating point scores that are rounded to *ε *decimal places, the time and space requirement increases by a factor of 10^*ε*^. Conversely, if all integer scores share a greatest common divisor *z*, the matrix should be canceled down by *z*.

#### Restricted probability computation

**Figure 12:**
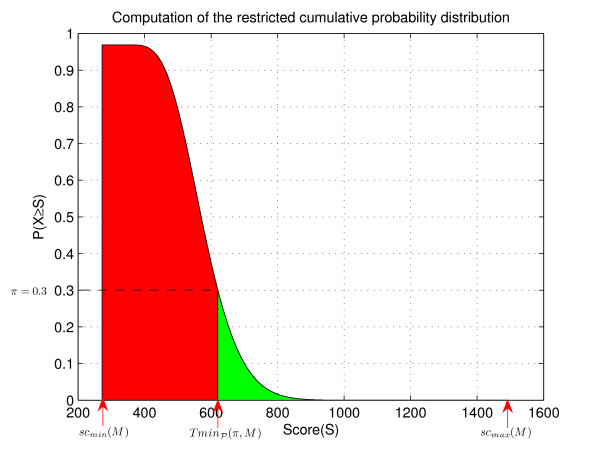
**Restricted probability computation**. Computation of the partial cumulative distribution function. Observe that in order to determine TminP
 MathType@MTEF@5@5@+=feaafiart1ev1aaatCvAUfKttLearuWrP9MDH5MBPbIqV92AaeXatLxBI9gBamrtHrhAL1wy0L2yHvtyaeHbnfgDOvwBHrxAJfwnaebbnrfifHhDYfgasaacH8akY=wiFfYdH8Gipec8Eeeu0xXdbba9frFj0=OqFfea0dXdd9vqai=hGuQ8kuc9pgc9s8qqaq=dirpe0xb9q8qiLsFr0=vr0=vr0dc8meaabaqaciaacaGaaeqabaWaaeGaeaaakeaacqWGubavieGacqWFTbqBcqWFPbqAcqWFUbGBdaWgaaWcbaGaemiuaafabeaaaaa@3D07@(*π*, *M*) for *π *= 0.3 we do not have to calculate the complete distribution in the score range [*sc*_min_(*M*), *sc*_max_(*M*)]. It is sufficient to calculate only the upper end (green area) starting with *sc*_max_(*M*) until ℙ[*X *≥ *S*] ≥ *π*.

In order to find TminP
 MathType@MTEF@5@5@+=feaafiart1ev1aaatCvAUfKttLearuWrP9MDH5MBPbIqV92AaeXatLxBI9gBamrtHrhAL1wy0L2yHvtyaeHbnfgDOvwBHrxAJfwnaebbnrfifHhDYfgasaacH8akY=wiFfYdH8Gipec8Eeeu0xXdbba9frFj0=OqFfea0dXdd9vqai=hGuQ8kuc9pgc9s8qqaq=dirpe0xb9q8qiLsFr0=vr0=vr0dc8meaabaqaciaacaGaaeqabaWaaeGaeaaakeaacqWGubavieGacqWFTbqBcqWFPbqAcqWFUbGBdaWgaaWcbaGaemiuaafabeaaaaa@3D07@(*π*, *M*) it is not necessary to compute the whole codomain of the distribution function *Q *= *Q*_*m*-1_. We propose a new method only computing a partial distribution by summing over the probabilities for decreasing threshold values *sc*_max _(*M*), *sc*_max _(*M*) - 1,..., until the given p-value *π *is exceeded (see Figures 11, 12).

In step *d *we compute *Q *(*sc*_max _(*M*) - *d*) where all intermediate scores contributing to *sc*_max _(*M*) - *d *have to be considered. In analogy to lookahead scoring, in each row *j *of *M *we avoid all intermediate scores below the intermediate threshold *th*_*j *_because they do not contribute to *Q*(*sc*_max _(*M*) - *d*). The algorithm stops if the cumulated probability for threshold *sc*_max _(*M*) - *d *exceeds the given p-value *π *and we obtain TminP
 MathType@MTEF@5@5@+=feaafiart1ev1aaatCvAUfKttLearuWrP9MDH5MBPbIqV92AaeXatLxBI9gBamrtHrhAL1wy0L2yHvtyaeHbnfgDOvwBHrxAJfwnaebbnrfifHhDYfgasaacH8akY=wiFfYdH8Gipec8Eeeu0xXdbba9frFj0=OqFfea0dXdd9vqai=hGuQ8kuc9pgc9s8qqaq=dirpe0xb9q8qiLsFr0=vr0=vr0dc8meaabaqaciaacaGaaeqabaWaaeGaeaaakeaacqWGubavieGacqWFTbqBcqWFPbqAcqWFUbGBdaWgaaWcbaGaemiuaafabeaaaaa@3D07@(*π*, *M*) = *sc*_max _(*M*) - *d *+ 1.

#### Lazy evaluation of the permuted matrix

The restricted computation strategy performs best if there are only few iterations (i.e., TminP
 MathType@MTEF@5@5@+=feaafiart1ev1aaatCvAUfKttLearuWrP9MDH5MBPbIqV92AaeXatLxBI9gBamrtHrhAL1wy0L2yHvtyaeHbnfgDOvwBHrxAJfwnaebbnrfifHhDYfgasaacH8akY=wiFfYdH8Gipec8Eeeu0xXdbba9frFj0=OqFfea0dXdd9vqai=hGuQ8kuc9pgc9s8qqaq=dirpe0xb9q8qiLsFr0=vr0=vr0dc8meaabaqaciaacaGaaeqabaWaaeGaeaaakeaacqWGubavieGacqWFTbqBcqWFPbqAcqWFUbGBdaWgaaWcbaGaemiuaafabeaaaaa@3D07@(*π*, *M*) is close to *sc*_max_(*M*)) and in each iteration step the computation of *Q*_*k*_(*t*) can be skipped in an early stage, i.e., for small values of *k*. The latter occurs to be more likely if the first rows of *M *contain strongly discriminative values leading to the exclusion of the small values by comparison with the intermediate thresholds. An example of this situation is given in Figure 1. Since *Q*_*k*_(*t*) is invariant to the permutation of the rows of *M*, we can sort the rows of *M *such that the most discriminative rows come first. We found that the difference between the largest two values of a row is a suitable measure for the level of discrimination since a larger difference increases the probability to remain below the intermediate threshold. Since the rows of *M *are scanned several times, we save time by initially sorting each row in order of descending score. We divide the computation steps where the step *d *computes *Q*(*sc*_max_(*M*) - *d*): In step *d *= 0 only the maximal scores max_*i*_, *i *∈ [0, *m *- 1] in each row have to be evaluated.

**Figure 13:**
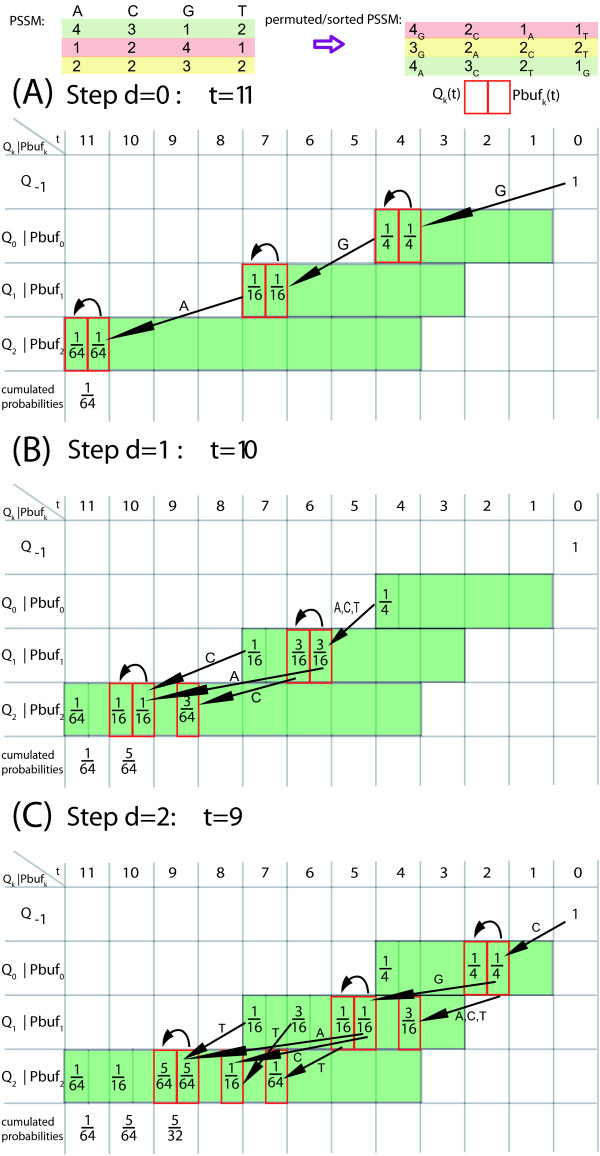
**Probability computation using lazy evaluation ofthe DP matrix**. In this example we use the same PSSM *M*, character distribution, and p-value threshold *π *= 18
 MathType@MTEF@5@5@+=feaafiart1ev1aaatCvAUfKttLearuWrP9MDH5MBPbIqV92AaeXatLxBI9gBaebbnrfifHhDYfgasaacH8akY=wiFfYdH8Gipec8Eeeu0xXdbba9frFj0=OqFfea0dXdd9vqai=hGuQ8kuc9pgc9s8qqaq=dirpe0xb9q8qiLsFr0=vr0=vr0dc8meaabaqaciaacaGaaeqabaqabeGadaaakeaadaWcaaqaaiabigdaXaqaaiabiIda4aaaaaa@2EAA@ as in Figure 11. However, in each row of the PSSM the scores are sorted in descending order, and the rows are sorted with the most discriminant row coming first (see coloured PSSMs for this relationship). Observe that the *LazyDistrib *algorithm evaluates the DP vectors non-recursively top-down. Cells computed in the actual step are marked red. In step *d *= 0 the algorithm computes *Q*_2_(11) by evaluating paths through the PSSM contributing to *Q*_2_(ll), which is in this example only the high scoring path GGA. Intermediate results of *Q*_0_(4), *Q*_1_(7), and *Q*_2_(11) are collected in buffers *Pbuf*_0_(4), *Pbuf*_1_(7), and *Pbuf*_2_(11) first, and finally copied to the correponding cells in *Q*. See (A) for the situation after step *d *= 0 has been completed. In step *d *= 1, see (B), the algorithm computes *Q*_2_(10), starting in row *k *= 1 with the determination of *Pbuf*_1_(6) and *Q*_1_(6). That is, *Q*_1_(6) = *Pbuf*_1_(6) = *Q*_0_(4)·*f*(*A*) + *Q*_0_(4)·*f*(*C*) + *Q*_0_(4)·*f*(*T*) = 316
 MathType@MTEF@5@5@+=feaafiart1ev1aaatCvAUfKttLearuWrP9MDH5MBPbIqV92AaeXatLxBI9gBaebbnrfifHhDYfgasaacH8akY=wiFfYdH8Gipec8Eeeu0xXdbba9frFj0=OqFfea0dXdd9vqai=hGuQ8kuc9pgc9s8qqaq=dirpe0xb9q8qiLsFr0=vr0=vr0dc8meaabaqaciaacaGaaeqabaqabeGadaaakeaadaWcaaqaaiabiodaZaqaaiabigdaXiabiAda2aaaaaa@2F9A@. Analogously *Q*_2_(10) and *Pbuf*_2_(10) are computed based on *Q*_1_(7) and *Q*_1_(6). Additionally *Pbuf*_2_(9) is filled for further reuse in subsequent steps *d *+ 1, *d *+ 2,.... We compute *Pbuf*_2_(9) = *Q*_1_(6)·*f*(*C*) = 364
 MathType@MTEF@5@5@+=feaafiart1ev1aaatCvAUfKttLearuWrP9MDH5MBPbIqV92AaeXatLxBI9gBaebbnrfifHhDYfgasaacH8akY=wiFfYdH8Gipec8Eeeu0xXdbba9frFj0=OqFfea0dXdd9vqai=hGuQ8kuc9pgc9s8qqaq=dirpe0xb9q8qiLsFr0=vr0=vr0dc8meaabaqaciaacaGaaeqabaqabeGadaaakeaadaWcaaqaaiabiodaZaqaaiabiAda2iabisda0aaaaaa@2FA0@. The algorithm can directly start in row *k *= 1 with the computation of *Q*_1_(6) instead of *Q*_0_(3) since a score of 3 cannot be achieved by the first prefix PSSM *M*_0_. Only score 4 of *M*_0 _contributes to *Q*_2_(10), scores 2 and 1 do not. In step *d *= 2, see (C), the algorithm computes *Q*_2_(9), starting in row *k *= 0. *Pbuf*_2_(9) is computed reusing the partial sum calculated in previous steps, such that *Pbuf*_2_(9) = 364
 MathType@MTEF@5@5@+=feaafiart1ev1aaatCvAUfKttLearuWrP9MDH5MBPbIqV92AaeXatLxBI9gBaebbnrfifHhDYfgasaacH8akY=wiFfYdH8Gipec8Eeeu0xXdbba9frFj0=OqFfea0dXdd9vqai=hGuQ8kuc9pgc9s8qqaq=dirpe0xb9q8qiLsFr0=vr0=vr0dc8meaabaqaciaacaGaaeqabaqabeGadaaakeaadaWcaaqaaiabiodaZaqaaiabiAda2iabisda0aaaaaa@2FA0@ + *Q*_1_(7)·*f*(*T*) + *Pbuf*_1_(5)·*f*(*A*) = 564
 MathType@MTEF@5@5@+=feaafiart1ev1aaatCvAUfKttLearuWrP9MDH5MBPbIqV92AaeXatLxBI9gBaebbnrfifHhDYfgasaacH8akY=wiFfYdH8Gipec8Eeeu0xXdbba9frFj0=OqFfea0dXdd9vqai=hGuQ8kuc9pgc9s8qqaq=dirpe0xb9q8qiLsFr0=vr0=vr0dc8meaabaqaciaacaGaaeqabaqabeGadaaakeaadaWcaaqaaiabiwda1aqaaiabiAda2iabisda0aaaaaa@2FA4@, and then copied to *Q*_2_(9). *Pbuf*_1_(4), *Pbuf*_2_(8), and *Pbuf*_2_(7) are filled based on *Pbuf*_0_(2), *Q*_1_(6), *Pbuf*_1_(5), and *Q*_1_(5) for further reuse. After step *d *= 2 the rest of the computation can be skipped since the cumulated probability *Q*_2_(11) + *Q*_2_(10) + *Q*_2_(9) = 532
 MathType@MTEF@5@5@+=feaafiart1ev1aaatCvAUfKttLearuWrP9MDH5MBPbIqV92AaeXatLxBI9gBaebbnrfifHhDYfgasaacH8akY=wiFfYdH8Gipec8Eeeu0xXdbba9frFj0=OqFfea0dXdd9vqai=hGuQ8kuc9pgc9s8qqaq=dirpe0xb9q8qiLsFr0=vr0=vr0dc8meaabaqaciaacaGaaeqabaqabeGadaaakeaadaWcaaqaaiabiwda1aqaaiabiodaZiabikdaYaaaaaa@2F9A@ exceeds the given p-value *π *= 18
 MathType@MTEF@5@5@+=feaafiart1ev1aaatCvAUfKttLearuWrP9MDH5MBPbIqV92AaeXatLxBI9gBaebbnrfifHhDYfgasaacH8akY=wiFfYdH8Gipec8Eeeu0xXdbba9frFj0=OqFfea0dXdd9vqai=hGuQ8kuc9pgc9s8qqaq=dirpe0xb9q8qiLsFr0=vr0=vr0dc8meaabaqaciaacaGaaeqabaqabeGadaaakeaadaWcaaqaaiabigdaXaqaaiabiIda4aaaaaa@2EAA@ and we obtain a score threshold of *th *= 10 corresponding to *π*.

In step *d *> 0 all scores *M*(*i*, *a*) ≥ max_*i *_- *d *may contribute to *Q*(*sc*_max_(*M*) - *d*). Since in general a score value *M*(*i*, *a*) ≥ max_*i *_- *d *also gives contribution to *Q*(*sc*_max_(*M*) - *l*) for *l *> *d*, we can save time by storing *Q*_*i*_(max_*i *_- *l*) for *l *> *d*, in step *d *in a buffer and reusing the buffer in steps *d *+ 1, *d *+ 2,.... This allows for the computation of *Q*_*k*_(*sc*_max_(*M*) - *d*) only based on the buffer and scores *M*(*i*, *a*) = max_*i *_- *d *while scores *M*(*i*, *a*) > max_*i *_- *d*, *i *∈ [0, *m *- 1], can be omitted. We therefore have developed an algorithm *LazyDistrib *employing lazy evaluation of the distribution. That is, given a threshold *th*, the algorithm only evaluates parts of the DP vectors necessary to determine *Q*_*k*_(*th*) and simultaneously saves sub-results concerned with score *th *in an additional buffer matrix *Pbuf *(instead of recomputing them later, see Figure 13 for an example). This is described by the following recurrence:

Qk(th−d)=Pbufk(th−d)+∑a∈A:M(k,a)≥max⁡k−dQk−1(th−d−M(k,a))f(a)Pbufk(th−d):=∑a∈A:M(k,a)<max⁡k−dQk−1(th−d−M(k,a))f(a)
 MathType@MTEF@5@5@+=feaafiart1ev1aaatCvAUfKttLearuWrP9MDH5MBPbIqV92AaeXatLxBI9gBamrtHrhAL1wy0L2yHvtyaeHbnfgDOvwBHrxAJfwnaebbnrfifHhDYfgasaacH8akY=wiFfYdH8Gipec8Eeeu0xXdbba9frFj0=OqFfea0dXdd9vqai=hGuQ8kuc9pgc9s8qqaq=dirpe0xb9q8qiLsFr0=vr0=vr0dc8meaabaqaciaacaGaaeqabaWaaeGaeaaakeaafaqadeWadaaabaGaemyuae1aaSbaaSqaaiabdUgaRbqabaGccqGGOaakcqWG0baDcqWGObaAcqGHsislcqWGKbazcqGGPaqkaeaacqGH9aqpaeaacqWGqbaucqWGIbGycqWG1bqDcqWGMbGzdaWgaaWcbaGaem4AaSgabeaakiabcIcaOiabdsha0jabdIgaOjabgkHiTiabdsgaKjabcMcaPiabgUcaRaqaaaqaaaqaamaaqafabaGaemyuae1aaSbaaSqaaiabdUgaRjabgkHiTiabigdaXaqabaGccqGGOaakcqWG0baDcqWGObaAcqGHsislcqWGKbazcqGHsislcqWGnbqtcqGGOaakcqWGRbWAcqGGSaalcqWGHbqycqGGPaqkcqGGPaqkcqWGMbGzcqGGOaakcqWGHbqycqGGPaqkaSqaaiabdggaHjabgIGioJWaaiab=bq8bjabcQda6iabd2eanjabcIcaOiabdUgaRjabcYcaSiabdggaHjabcMcaPiabgwMiZkGbc2gaTjabcggaHjabcIha4naaBaaameaacqWGRbWAaeqaaSGaeyOeI0IaemizaqgabeqdcqGHris5aaGcbaGaemiuaaLaemOyaiMaemyDauNaemOzay2aaSbaaSqaaiabdUgaRbqabaGccqGGOaakcqWG0baDcqWGObaAcqGHsislcqWGKbazcqGGPaqkaeaacqGG6aGocqGH9aqpaeaadaaeqbqaaiabdgfarnaaBaaaleaacqWGRbWAcqGHsislcqaIXaqmaeqaaOGaeiikaGIaemiDaqNaemiAaGMaeyOeI0IaemizaqMaeyOeI0Iaemyta0KaeiikaGIaem4AaSMaeiilaWIaemyyaeMaeiykaKIaeiykaKIaemOzayMaeiikaGIaemyyaeMaeiykaKcaleaacqWGHbqycqGHiiIZcqWFaeFqcqGG6aGocqWGnbqtcqGGOaakcqWGRbWAcqGGSaalcqWGHbqycqGGPaqkcqGH8aapcyGGTbqBcqGGHbqycqGG4baEdaWgaaadbaGaem4AaSgabeaaliabgkHiTiabdsgaKbqab0GaeyyeIuoaaaaaaa@BBBD@

In the present implementation, the algorithm assumes independently distributed symbols. The algorithm can be extended to an order *d*-Markov model (w.r.t. the background alphabet distribution). This increases the computation time by a factor of |A
 MathType@MTEF@5@5@+=feaafiart1ev1aaatCvAUfKttLearuWrP9MDH5MBPbIqV92AaeXatLxBI9gBamrtHrhAL1wy0L2yHvtyaeHbnfgDOvwBHrxAJfwnaebbnrfifHhDYfgasaacH8akY=wiFfYdH8Gipec8Eeeu0xXdbba9frFj0=OqFfea0dXdd9vqai=hGuQ8kuc9pgc9s8qqaq=dirpe0xb9q8qiLsFr0=vr0=vr0dc8meaabaqaciaacaGaaeqabaWaaeGaeaaakeaaimaacqWFaeFqaaa@3821@|^*d*^.

### Implementation and computational results

We implemented *LAsearch*, *ESAsearch*, both capable to handle reduced alphabets, and *LazyDistrib *in C. The program was compiled with the GNU C compiler (version 3.1, optimization option -03). All measurements were performed on a 8 CPU Sun UltraSparc III computer running at 900 MHz, with 64 GB main memory (using only one CPU and a small fraction of the memory). Enhanced suffix arrays were constructed with the program mkvtree, see [34].

**Figure 14:**
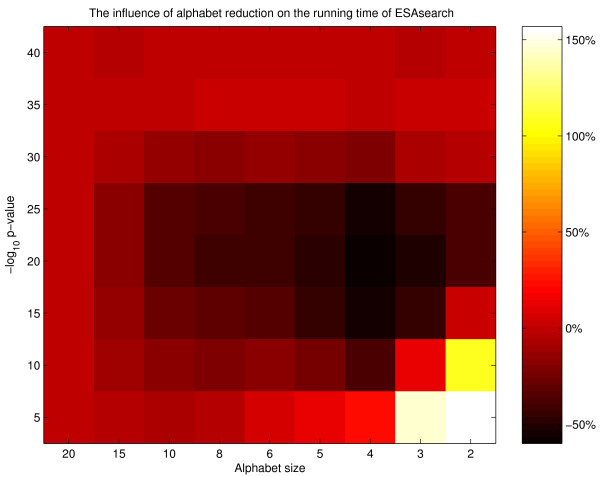
**Effect of alphabet reduction on the running time of ESAsearch**. Experiment 6: Relative deviations of running time of *ESAsearch *when using reduced alphabets at different levels of stringency. We measured the relative percentage deviation with respect to the running time when using the standard 20 letter amino acid alphabet (= 0%). We searched with 11,411 PSSMs from the PRINTS database (Rel. 38) in the RCSB Protein Data Bank (PDB) with a total sequence length of 4.3 MB. In this example, the maximum performance improvement is achieved for an alphabet of size 4 and a p-value cutoff of *π *= 10^-20^.

**Figure 15:**
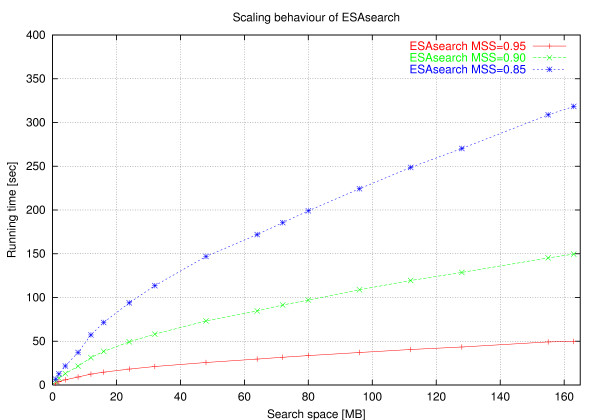
**Scaling behaviour of ESAsearch**. Experiment 7: Scaling behavior of *ESAsearch *when searching with 576 TRANSFAC PSSMs on subsets of human chromosome 6 of different sizes and with different matrix similarity cutoff values (MSS). The subsets are prefixes of human chromosome 6 of length 2^*k *^for *k *= 0, 1, 2,..., 7.

**Figure 16:**
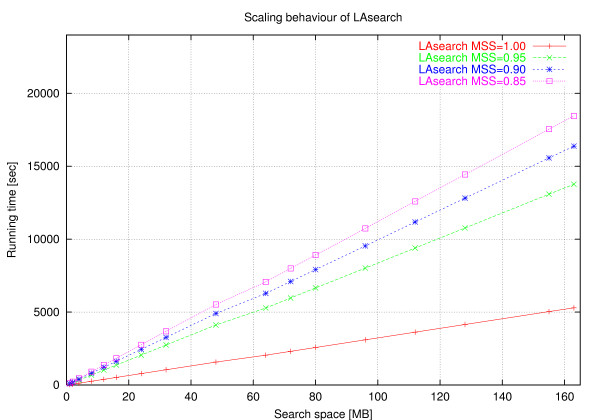
**Scaling behaviour of LAsearch**. Experiment 7: Scaling behavior of *LAsearch *when searching with 576 TRANSFAC PSSMs on subsets of human chromosome 6 of different sizes and with different matrix similarity cutoff values (MSS). The subsets are prefixes of human chromosome 6 of length 2^*k *^for *k *= 0, 1, 2,..., 7.

We performed seven experiments comparing different programs for searching PSSMs. Table 1 gives more details on the experimental input for Experiments 1–6. Results are given in Table 2 (Exp. 1–5) and Figure 14 (Exp. 6). For Experiment 7, see Figures 15 and 16. In these experiments *ESAsearch *performed very well, especially on nucleotide PSSMs, see Experiments 2 and 4. It is faster than *MatInspector *by a factor between 63 and 1,037, depending on the stringency of the given thresholds. The commercial advancement of *MatInspector*, called *MATCH*, was not available for our comparisons, but based on [7] we presume a running time comparable to *MatInspector*. Compared to *LAsearch*, *ESAsearch *is faster by a factor between 17 (MSS = 0.80) and 196 (MSS = 0.95) (see Experiment 2). On larger nucleotide sequences (see Experiment 4) the speedup factors increase, ranging from 58 (MSS = 0.85) to 275 (MSS = 0.95). See Table 1 for the definition of MSS. In the experiments using protein PSSMs, *ESAsearch *is faster than the method of [13] by a factor between 1.5 and 1.8 (see Experiment 1). This is due to the better locality behavior of the enhanced suffix array compared to a suffix tree. For larger p-values *LAsearch *performs slightly better than *ESAsearch*. Increasing the stringency, the performance of *ESAsearch *increases, resulting in a speedup of factor 1.5 for a p-value of 10^-40^. We explain this behavior by the larger alphabet size, resulting in shorter common prefixes and therefore smaller skipped areas of the enhanced suffix array. With increasing stringency of the threshold, the expected reading depth decreases, resulting in larger skipped areas of the enhanced suffix array. Compared to the *FingerPrintScan *program, *ESAsearch *achieves a speedup factor between 3.8 and 470, see Experiment 3. In comparison to *Blimps*, the PSSM-searching program of the BLOCKS database, *ESAsearch *is faster by a factor of 23 (see Experiment 5) for the chosen threshold. In Experiment 6 (see Figure 14), we measured the influence of alphabet reductions on the running time of *ESAsearch *when using protein PSSMs. Compared to the performance of *ESAsearch *operating on the normal 20 letter amino acid alphabet a speedup up to factor 2 can be achieved when using a 4 letter alphabet and a p-value cutoff of 10^-20^. Experiment 7 (see Figures 15 and 16) shows that the expected running time of *ESAsearch *is sublinear, whereas *LAsearch *runs in linear time. In a final experiment, we compared algorithm *LazyDistrib *with the DP-algorithm computing the complete distribution. *LazyDistrib *shows a speedup factor between 3 and 330 on our test set, depending on the stringency of the threshold (see Table 3).

**Table 1:** Performed experiments and experimental input.

	Exp. 1	Exp. 2	Exp. 3	Exp. 4	Exp. 5	Exp. 6
# searched sequences	59,021	30,964	19,111	1 (*H.s*. Chr. 6)	19,111	19,111
total length	20.2 MB	37.2 MB	4.3 MB	162.9 MB	4.3 MB	4.3 MB
sequence source	see [13]	DBTSS 5.1	RCSB PDB	Sanger V1. 4	RCSB PDB	RCSB PDB
sequence type/PSSM type	protein	DNA	protein	DNA	protein	protein
# PSSMs	4,034	220	11,411	576	28,337	10,931
PSSM source	see [13]	MatInspector	PRINTS 38	TRANSFAC Prof. 6.2	BLOCKS 14.1	PRINTS 38
avg. length of PSSMs	29.74	14.21	17.32	13.33	26.3	17.37
index construction (sec)	41	146	10.2	586	10.2	10.2
*mdc *(sec)	1960	-	1486	-	11871	1486

*MatInspector*		x				
*FingerPrintScan*			x			
*Blimps*					x	
*DN00*	x					
*LAsearch*	x	x	x	x	x	
*ESAsearch*	x	x	x	x	x	x
*ESAsearch *(reduced A MathType@MTEF@5@5@+=feaafiart1ev1aaatCvAUfKttLearuWrP9MDH5MBPbIqV92AaeXatLxBI9gBamrtHrhAL1wy0L2yHvtyaeHbnfgDOvwBHrxAJfwnaebbnrfifHhDYfgasaacH8akY=wiFfYdH8Gipec8Eeeu0xXdbba9frFj0=OqFfea0dXdd9vqai=hGuQ8kuc9pgc9s8qqaq=dirpe0xb9q8qiLsFr0=vr0=vr0dc8meaabaqaciaacaGaaeqabaWaaeGaeaaakeaaimaacqWFaeFqaaa@3821@)						x

Overview of the sequences and PSSMs used in the performed experiments. For the experiments that use p-value or E-value cutoffs, we precomputed the cumulative score distributions and stored them on file. *mdc *is the time needed for this task. In Experiment 1 we measured the running time of the Java-program from [13], referred to by *DN00*. We ran *DN00 *with a maximum of 2 GB memory assigned to the Java virtual machine. *DN00 *constructs the suffix tree in main memory and then performs the searches. For a fair comparison, we therefore measured the total running time, and the time for matching the PSSMs (without suffix tree construction). For Experiment 2, we implemented the matrix similarity scoring scheme (MSS) of *MatInspector *and matched the PSSMs against both strands of the DNA sequences with different MSS cutoff values. The MSS of PSSM *M *of length *m *and a sequence *w *∈ A
 MathType@MTEF@5@5@+=feaafiart1ev1aaatCvAUfKttLearuWrP9MDH5MBPbIqV92AaeXatLxBI9gBamrtHrhAL1wy0L2yHvtyaeHbnfgDOvwBHrxAJfwnaebbnrfifHhDYfgasaacH8akY=wiFfYdH8Gipec8Eeeu0xXdbba9frFj0=OqFfea0dXdd9vqai=hGuQ8kuc9pgc9s8qqaq=dirpe0xb9q8qiLsFr0=vr0=vr0dc8meaabaqaciaacaGaaeqabaWaaeGaeaaakeaaimaacqWFaeFqaaa@3821@^*m *^is defined as MSS=sc(w,M)−scmin⁡(M)scmax⁡(M)−scmin⁡(M)
 MathType@MTEF@5@5@+=feaafiart1ev1aaatCvAUfKttLearuWrP9MDH5MBPbIqV92AaeXatLxBI9gBaebbnrfifHhDYfgasaacH8akY=wiFfYdH8Gipec8Eeeu0xXdbba9frFj0=OqFfea0dXdd9vqai=hGuQ8kuc9pgc9s8qqaq=dirpe0xb9q8qiLsFr0=vr0=vr0dc8meaabaqaciaacaGaaeqabaqabeGadaaakeaacqqGnbqtcqqGtbWucqqGtbWucqGH9aqpdaWcaaqaaiabdohaZjabdogaJnaabmGabaGaem4DaCNaeiilaWIaemyta0eacaGLOaGaayzkaaGaeyOeI0Iaem4CamNaem4yam2aaSbaaSqaaiGbc2gaTjabcMgaPjabc6gaUbqabaGcdaqadiqaaiabd2eanbGaayjkaiaawMcaaaqaaiabdohaZjabdogaJnaaBaaaleaacyGGTbqBcqGGHbqycqGG4baEaeqaaOWaaeWaceaacqWGnbqtaiaawIcacaGLPaaacqGHsislcqWGZbWCcqWGJbWydaWgaaWcbaGagiyBa0MaeiyAaKMaeiOBa4gabeaakmaabmGabaGaemyta0eacaGLOaGaayzkaaaaaaaa@582A@ and hence given an MSS cutoff value, the threshold *th *is determined as *th *= MSS·(*sc*_max_(*M*) - *sc*_min_(*M*)) + *sc*_min_(*M*). Instead of using the reverse strand we use the reverse complement M¯
 MathType@MTEF@5@5@+=feaafiart1ev1aaatCvAUfKttLearuWrP9MDH5MBPbIqV92AaeXatLxBI9gBaebbnrfifHhDYfgasaacH8akY=wiFfYdH8Gipec8Eeeu0xXdbba9frFj0=OqFfea0dXdd9vqai=hGuQ8kuc9pgc9s8qqaq=dirpe0xb9q8qiLsFr0=vr0=vr0dc8meaabaqaciaacaGaaeqabaqabeGadaaakeaadaqdaaqaaiabd2eanbaaaaa@2DE0@ of the PSSM *M*, defined by M¯
 MathType@MTEF@5@5@+=feaafiart1ev1aaatCvAUfKttLearuWrP9MDH5MBPbIqV92AaeXatLxBI9gBaebbnrfifHhDYfgasaacH8akY=wiFfYdH8Gipec8Eeeu0xXdbba9frFj0=OqFfea0dXdd9vqai=hGuQ8kuc9pgc9s8qqaq=dirpe0xb9q8qiLsFr0=vr0=vr0dc8meaabaqaciaacaGaaeqabaqabeGadaaakeaadaqdaaqaaiabd2eanbaaaaa@2DE0@(*i*, *a*) = *M*(*m *- 1 - *i*, a¯
 MathType@MTEF@5@5@+=feaafiart1ev1aaatCvAUfKttLearuWrP9MDH5MBPbIqV92AaeXatLxBI9gBaebbnrfifHhDYfgasaacH8akY=wiFfYdH8Gipec8Eeeu0xXdbba9frFj0=OqFfea0dXdd9vqai=hGuQ8kuc9pgc9s8qqaq=dirpe0xb9q8qiLsFr0=vr0=vr0dc8meaabaqaciaacaGaaeqabaqabeGadaaakeaadaqdaaqaaiabdggaHbaaaaa@2E08@) for all *i *∈ [0, *m *- 1] and *a *∈ A
 MathType@MTEF@5@5@+=feaafiart1ev1aaatCvAUfKttLearuWrP9MDH5MBPbIqV92AaeXatLxBI9gBamrtHrhAL1wy0L2yHvtyaeHbnfgDOvwBHrxAJfwnaebbnrfifHhDYfgasaacH8akY=wiFfYdH8Gipec8Eeeu0xXdbba9frFj0=OqFfea0dXdd9vqai=hGuQ8kuc9pgc9s8qqaq=dirpe0xb9q8qiLsFr0=vr0=vr0dc8meaabaqaciaacaGaaeqabaWaaeGaeaaakeaaimaacqWFaeFqaaa@3821@, where a¯
 MathType@MTEF@5@5@+=feaafiart1ev1aaatCvAUfKttLearuWrP9MDH5MBPbIqV92AaeXatLxBI9gBaebbnrfifHhDYfgasaacH8akY=wiFfYdH8Gipec8Eeeu0xXdbba9frFj0=OqFfea0dXdd9vqai=hGuQ8kuc9pgc9s8qqaq=dirpe0xb9q8qiLsFr0=vr0=vr0dc8meaabaqaciaacaGaaeqabaqabeGadaaakeaadaqdaaqaaiabdggaHbaaaaa@2E08@ is the Watson Crick complement of nucleotide *a*. This allows to use the same enhanced suffix array for both strands. In Experiment 5 we used a PERL-based wrapper for the *Blimps *program shipped with the BLIMPS distribution to do bulk sequence searches. The overhead for the PERL interpreter call was found to be negligible. For Experiment 6 we used the reduced alphabets given in Figure 8. The last seven rows show which programs were used in which experiment.

**Table 2:** Results of Experiments 1–5.

Experiment 1: 4,034 PSSMs in 20.2 MB protein sequences				
p-value	*DN00 *(total time)	*DN00 *(search)	*LAsearch*	*ESAsearch *+41 sec.

10^-10^	65,808	64,939	39,839	41,813
10^-20^	38,773	37,706	23,786	24,378
10^-30^	21,449	20,362	14,111	13,084
10^-40^	9,606	8,533	8,067	5,374

Experiment 2: 220 PSSMs in 37.2 MB DNA				

MSS	*MatInspector*		*LAsearch*	*ESAsearch *+32 sec.

0.80	12,773		3,605	202
0.85	12,567		3,189	108
0.90	12,487		2,818	53
0.95	12,445		2,356	12
1.00	12,429		885	1

Experiment 3: 11,411 PSSMs in 4.3 MB protein sequences				

E-value	*FingerPrintScan*		*LAsearch*	*ESAsearch *+10.2 sec.

10^-10^	4,733		3,423	1.244
10^-20^	4,710		486	52
10^-30^	4,706		27	10

Experiment 4: 576 PSSMs in 162.9 MB DNA				

MSS			*LAsearch*	*ESAsearch *+586 sec.

0.85			18,446	318
0.90			16,376	150
0.95			13,764	50
1.00			5,294	1

Experiment 5: 28,337 PSSMs in 4.3 MB protein sequences				

raw-*th*	*Blimps*		*LAsearch*	*ESAsearch *+10.2 sec.

945	271:30:16		16:03:12	11:35:58

Experiment 1: Running times in seconds of the different PSSM searching methods at different levels of stringency, when searching for 4,034 amino acid PSSMs in 59,021 sequences (21.2 MB) from SwissProt. These are the same PSSMs and sequences used in the experiments of [13]. Experiment 2: Running times in seconds of *MatInspector*, *LAsearch*, and *ESAsearch*, when searching 220 PSSMs on both strands of 37.2 MB DNA sequence data at different matrix similarity score (MSS) cutoffs. Experiment 3: Running times in seconds of *FingerPrintScan*, *LAsearch*, and *ESAsearch *when searching all 11,411 PSSMs from the PRINTS database in the RCSB protein data bank (PDB) for different E-values. Experiment 4: Running times in seconds of *LAsearch *and *ESAsearch *when searching 576 PSSMs in H. sapiens chr. 6 at different matrix similarity score (MSS) cutoffs. Experiment 5: Running times in hh:mm:ss of *Blimps*, *LAsearch*, and *ESAsearch *when searching all 28,337 PSSMs from the BLOCKS database in PDB. We used a raw score threshold of 945 as suggested in the *Blimps *documentation for searching large databases. For each experiment, the additional time needed for the construction of the enhanced suffix array is shown in the head of the *ESAsearch *column.

**Table 3:** Running times of the LazyDistrib algorithm.

p-value	simple DP	*LazyDistrib*	speedup factor
10^-10^	1,486	485.8	3
10^-20^	1,486	92.5	95
10^-30^	1,486	8.9	166
10^-40^	1,486	4.5	330

Running times in seconds when computing score thresholds for all 11,411 PSSMs from the PRINTS database (Rel. 38), given different p-values. Running times given in this table are measurements performed with improved versions of the simple DP and *LazyDistrib *algorithms and thus are much lower than the times given in [25].

#### PoSSuM software distribution

Our software tool *PoSSuMsearch *implements all algorithms and ideas presented in this work, namely *Simplesearch*, *LAsearch*, *ESAsearch *and *LazyDistrib*. A user can search for PSSMs in enhanced suffix arrays built by mkvtree from the *Vmatch *package, as well as on plain sequence data in FASTA, GENBANK, EMBL, or SWISSPROT format. The search algorithm can be chosen from the command line.

PSSMs are specified in a simple plain text format, where one file may contain multiple PSSMs. The alphabet a PSSM refers to, and alphabet character to PSSM column assignments can be specified on a per-PSSM basis for most flexible alphabet support. All implemented algorithms support alphabet transformations. PSSMs can contain integer as well as floating point scores. To prevent rounding errors for integer based PSSMs, *PoSSuMsearch *uses integer arithmetics for these, resulting in an additional speedup on most CPU architectures. Searching on the reverse strand of nucleotide sequences is implemented by PSSM transformation according to Watson-Crick base pairing. Hence it is sufficient to build the enhanced suffix array for one strand only. This can then be used to search both strands.

The cutoff can be specified as p-value, E-value, MSS (matrix similarity score), or raw score threshold. If only the best matches with the highest scores need to be known, then *PoSSuMsearch *can be asked to report only the *k *highest scoring matches without even specifying an explicit cutoff. To do so, the search algorithms dynamically adapt the threshold during the search. When using p- or E-values, the score threshold is determined by either the lazy dynamic programming algorithm introduced in this contribution, or read from file that stores the complete precalculated probability distribution. Background distributions can be specified arbitrarily by the user, or determined from a given sequence database. We provide a tool, *PoSSuMdist*, to generate a compressed file containing the complete precalculated probability distribution for a set of PSSMs.

PSSM matches can be sorted by specifying a list of sort keys, like p-value, match score, sequence number, and so on. The output formats of *PoSSuMsearch *print out all available information about a match, either in a human readable format, tab delimited, or in machine readable, XML-based CisML [35]. *PoSSuMsearch *as well as *PoSSuMdist *support multi-threading for a further reduction of running time on multi CPU machines.

The *PoSSuM *software distribution includes the searching tool *PoSSuMsearch *itself, and additional tools to determine character frequencies from sequence data, for probability distribution calculation, and PSSM format converters for *TRANSFAC*, *BLOCKS*, *PRINTS*, and *EMATRIX *style PSSMs.

## Discussion and conclusion

We have presented a new non-heuristic algorithm for searching with PSSMs, achieving expected sublinear running time. Our analysis of *ESAsearch *shows that for sequences not shorter than |A
 MathType@MTEF@5@5@+=feaafiart1ev1aaatCvAUfKttLearuWrP9MDH5MBPbIqV92AaeXatLxBI9gBamrtHrhAL1wy0L2yHvtyaeHbnfgDOvwBHrxAJfwnaebbnrfifHhDYfgasaacH8akY=wiFfYdH8Gipec8Eeeu0xXdbba9frFj0=OqFfea0dXdd9vqai=hGuQ8kuc9pgc9s8qqaq=dirpe0xb9q8qiLsFr0=vr0=vr0dc8meaabaqaciaacaGaaeqabaWaaeGaeaaakeaaimaacqWFaeFqaaa@3821@|^*m *^+ *m *- 1 a linear runtime in the worst case is achieved. It shows superior performance over the most widely used programs, especially for DNA sequences. The enhanced suffix array, on which the method is based, requires only 9*n *bytes. This is a space reduction of more than 45 percent compared to the 17*n *bytes implementation of [13]. Further on, we developed a systematic concept for alphabet reduction, especially useful on amino acid sequences and PSSMs for gaining additional speedup. Our third main contribution is a new algorithm for the efficient calculation of score thresholds from user defined E-values and p-values. The algorithm allows for accurate on-the-fly calculations of thresholds, and has the potential to replace formerly used approximation approaches. Beyond the algorithmic contributions, we provide a robust, well documented, and easy to use software package, implementing the ideas and algorithms presented in this manuscript.

## Availability

The *PoSSuM *software distribution and its documentation is available precompiled for different operating systems and architectures on [24]. A version of mkvtree is included. A web based version of *PoSSuMsearch *is available under the same URL.

## Authors' contributions

M.B. developed the algorithms presented in this manuscript, and wrote significant parts of the manuscript. R.H. implemented the algorithms, created the software distribution, and contributed to the manuscript. M.B. and R.H. wrote the documentation for the software package. R.G. provided supervision and guidance on the project and provided essential infrastructure. S.K. provided supervision, and contributed to the manuscript. All authors read and approved the final manuscript.
